# Human microbiota research in Africa: a systematic review reveals gaps and priorities for future research

**DOI:** 10.1186/s40168-021-01195-7

**Published:** 2021-12-15

**Authors:** Imane Allali, Regina E. Abotsi, Lemese Ah. Tow, Lehana Thabane, Heather J. Zar, Nicola M. Mulder, Mark P. Nicol

**Affiliations:** 1 Computational Biology Division, Department of Integrative Biomedical Sciences, University of Cape Town, Cape Town, South Africa; 2 Laboratory of Human Pathologies Biology, Department of Biology, Faculty of Sciences, and Genomic Centre of Human Pathologies, Faculty of Medicine and Pharmacy, Mohammed V University in Rabat, Rabat, Morocco; 3 Institute of Infectious Disease and Molecular Medicine, Faculty of Health Sciences, University of Cape Town, Cape Town, South Africa; 4 Department of Molecular and Cell Biology, Faculty of Science, University of Cape Town, Cape Town, South Africa; 5 Department of Pharmaceutical Microbiology, School of Pharmacy, University of Health and Allied Sciences, Ho, Ghana; 6 Division of Medical Microbiology, Department of Pathology, Faculty of Health Sciences, University of Cape Town, Cape Town, South Africa; 7 Department of Health Research Methods, Evidence and Impact, McMaster University, Hamilton, Ontario Canada; 8 Biostatistics Unit, Father Sean O’Sullivan Research Centre, St Joseph’s Healthcare, Hamilton, Ontario Canada; 9 Departments of Paediatrics and Anaesthesia, McMaster University, Hamilton, Ontario Canada; 10 Centre for Evaluation of Medicine, St Joseph’s Healthcare, Hamilton, Ontario Canada; 11 Population Health Research Institute, Hamilton Health Sciences, Hamilton, Ontario Canada; 12 Centre for Evidence-based Health Care, Faculty of Health Sciences, Stellenbosch University, Tygerberg, South Africa; 13 Department of Medicine, Faculty of Health Sciences, University of Cape Town, Cape Town, South Africa; 14 Department of Paediatrics and Child Health, Red Cross War Memorial Children’s Hospital, Cape Town, South Africa; 15 MRC Unit on Child & Adolescent Health, University of Cape Town, Cape Town, South Africa; 16 School of Biomedical Sciences, University of Western Australia, M504, Perth, WA 6009 Australia

**Keywords:** Microbiome, Next-generation sequencing, Systematic review, 16S rRNA sequencing, Metagenomics, Public health

## Abstract

**Background:**

The role of the human microbiome in health and disease is an emerging and important area of research; however, there is a concern that African populations are under-represented in human microbiome studies. We, therefore, conducted a systematic survey of African human microbiome studies to provide an overview and identify research gaps. Our secondary objectives were: (i) to determine the number of peer-reviewed publications; (ii) to identify the extent to which the researches focused on diseases identified by the World Health Organization [WHO] State of Health in the African Region Report as being the leading causes of morbidity and mortality in 2018; (iii) to describe the extent and pattern of collaborations between researchers in Africa and the rest of the world; and (iv) to identify leadership and funders of the studies.

**Methodology:**

We systematically searched Medline via PubMed, Scopus, CINAHL, Academic Search Premier, Africa-Wide Information through EBSCOhost, and Web of Science from inception through to 1st April 2020. We included studies that characterized samples from African populations using next-generation sequencing approaches. Two reviewers independently conducted the literature search, title and abstract, and full-text screening, as well as data extraction.

**Results:**

We included 168 studies out of 5515 records retrieved. Most studies were published in *PLoS One* (13%; 22/168), and samples were collected from 33 of the 54 African countries. The country where most studies were conducted was South Africa (27/168), followed by Kenya (23/168) and Uganda (18/168). 26.8% (45/168) focused on diseases of significant public health concern in Africa. Collaboration between scientists from the United States of America and Africa was most common (96/168). The first and/or last authors of 79.8% of studies were not affiliated with institutions in Africa. Major funders were the United States of America National Institutes of Health (45.2%; 76/168), Bill and Melinda Gates Foundation (17.8%; 30/168), and the European Union (11.9%; 20/168).

**Conclusions:**

There are significant gaps in microbiome research in Africa, especially those focusing on diseases of public health importance. There is a need for local leadership, capacity building, intra-continental collaboration, and national government investment in microbiome research within Africa.

Video Abstract

**Supplementary Information:**

The online version contains supplementary material available at 10.1186/s40168-021-01195-7.

## Microbiome research in Africa

**Table:** 

**What is known about this topic?**	**What are the gaps?**	**What does this study add to our knowledge?**
There is an exponential growth of microbiome studies in North America and Europe.	The number of African countries where microbiome studies were conducted is unknown.	Microbiome studies were conducted in 61% of the countries in Africa, with the top three being South Africa, Kenya, and Uganda.
Most of these microbiome studies are dedicated to understanding diseases of public health importance (e.g. cancers, irritable bowel disorder, diabetes, etc.) in these countries.	The extent to which these studies focused on diseases of public health significance in Africa remains uninvestigated.	Only 26.8% (45/168) of the studies focused on diseases of the highest public health importance in Africa, with HIV accounting for 64.4% (29/45).
	The leadership and pattern of collaboration in African human microbiome studies are unknown.	Non-Africans led 79.8% of all the studies, and the most collaborative efforts were between the United States of America and African scientists. There is the need for local leadership, capacity building, intra-continental collaboration, and national government investment in microbiome research within Africa.

## Introduction

The human microbiome plays pivotal roles in immune and brain development, nutrition, and metabolism [1, 2]. Imbalances in the gut microbiome have been associated with impairment and diseases of many organ systems [1] including cancers [3, 4], obesity [5], asthma [6, 7], allergy, inflammatory bowel disease, and metabolic diseases [1]. More recent reports have added sickle cell disease [8], brain disorders, and behaviors to the growing list of diseases [9]. Although the causal basis for many microbiome associations is unknown, the microbiome is likely to be key to precision medicine approaches [10].

In order for the microbiome field to contribute effectively to personalized medicine, it is imperative to draw an accurate picture of the human microbiome in health and disease. Almost all research into human health is dependent on context. This is particularly true for microbiome research as gut microbiomes, for example, vary extensively based on geography, age, diet, ethnicity, genetics, disease, medication, climate, and other environmental factors [1]. Consequently, there is an urgent need to characterize the microbiome of as many unique populations as possible.

The microbiomes of western populations have been extensively characterized; however, information regarding the microbiome of residents of Africa is considerably sparser. Microbiome studies extending our understanding of important diseases must be replicated in Africa due to context-specific factors [11]. In particular, environmental determinants may vary [12–15], and genomic heterogeneity [16] within the human population is more marked compared to other continents. Important environmental exposures include diet, geography, climate, infectious diseases, urbanization, living conditions, and pollution [11–14]. These variabilities preclude the generalization of microbiome studies conducted in one specific population in Africa to the entire continent. Therefore, the representation of diverse African participants in microbiome studies is a priority.

Although non-communicable diseases, including cancers, diabetes, and cardiovascular diseases, have emerged as public health threats in both developed and developing countries, Africa has an additional burden of infectious diseases [17]. Infections account for at least 70% of all deaths on the continent [18], including malaria, tuberculosis, HIV/AIDS, and neglected tropical diseases (Buruli ulcer, trypanosomiasis, schistosomiasis, and guinea worm) [17]. Lower respiratory infections, HIV/AIDS, diarrheal diseases, malaria, preterm birth complications, tuberculosis, neonatal sepsis/infections, stroke, and ischaemic heart diseases are responsible for the highest morbidity and mortality in Africa [19]. Health-related research in Africa, including microbiome-based research, must address the diseases that are of foremost public health importance.

A number of human microbiome studies have been conducted in Africa. Although Brewster and colleagues [14] have provided a survey of microbiome research conducted in Africa, this addressed only gut microbiome studies. Currently, no study has summarized all human microbiome research conducted in Africa in order to identify knowledge gaps and areas for further research. We, therefore, undertook a systematic survey of human microbiome studies involving African participants to provide an overview of and to identify research gaps in the field. Our secondary objectives were: (i) to determine the overall number of peer-reviewed publications; (ii) to identify the extent to which the researches focused on diseases identified by the WHO State of Health in the African Region Report 2018 as being the leading causes of morbidity and mortality [19]; (iii) to provide information on the extent and pattern of collaboration between researchers in Africa and the rest of the world; and  (iv) to identify leadership and the main funders of these studies.

## Materials and methods

### Search terms and strategy

This review followed the Preferred Reporting Items for Systematic Reviews and Meta-analyses (PRISMA) guidelines [20]. A comprehensive literature search was undertaken from inception through to 1st April 2020 using the following databases: Medline via PubMed, Scopus, ISI Web of Science (Web of Knowledge), and Academic Search Premier, Africa-Wide Information, and CINAHL via EBSCOhost according to the search strategy outlined in (Supplementary Table S1). No filters were applied to any of the searches. All citations were exported into ENDNOTE (X9; Thomson Reuters). The search was independently conducted by two reviewers IA and REA. The reference lists of reviews were searched for eligible papers that were not recovered by the search terms.

### Study selection criteria

Studies were included only if they meet all of the following criteria: (i) human studies involving residents of Africa only or as part of a multinational study regardless of age, sex, health status, study design, or care setting; (ii) published in English or French; and (iii) described either bacteria, archaea, fungi, viruses, or parasites identified from any human samples using next-generation sequencing (NGS) including both shotgun metagenomics and targeted amplicon sequencing. Our exclusion criteria were: (i) studies that did not include any human participants from African; (ii) those that utilized publicly available data on African participants; (iii) studies that did not characterize the microbiome; and (iii) studies that did not utilize NGS to characterize the microbiome or those that targeted only specific microorganisms in their analysis.

### Screening of studies

Records retrieved from the literature search of the six databases were independently downloaded into ENDNOTE (X9; Thomson Reuters) by two reviewers (IA and REA). These reviewers independently removed duplicates, reviews, commentaries, editorials, notes, news, and opinions. They then screened the title and abstract of residual articles against the inclusion and exclusion criteria. The full texts of the studies that passed this stage were retrieved. The reviewers proceeded to independently review these full texts based on the eligibility criteria. At each stage of the process, the two reviewers compared their results and disagreements were resolved by mutual discussion.

### Data extraction and synthesis

Once consensus was reached on which articles to include in the study, IA and REA independently extracted data into a predesigned data extraction table in Microsoft Excel^TM^. The data extracted included the country of origin of the samples; techniques used to analyze the microbiome, disease of focus, type of sample, participants metadata (number, age, gender, ethnicity, geographic region of the participants), aims and conclusions of the studies, whether the participants were from rural or urban settings, source of funding for the studies, country location of institutions to which the participating scientists were affiliated, name of the journal, first and last author’s information, and information on data availability. The extracted data were compared for accuracy and merged. IA and REA analyzed the merged data separately, and the results were compared for accuracy.

Under funding, any institute under the National Institute of Health (NIH) and European Union (EU) were captured as NIH and EU, respectively during the analysis. Furthermore, only agencies that directly funded the studies via project-specific grants were captured. Those that indirectly supported the research by providing training grants, scholarships, or fellowships to specific authors were not reported as funders. This is because our objective was to highlight organizations that directly funded human microbiome studies in Africa and we were not able to directly determine whether the funds from these sources were directly invested in the microbiome project reported.

Where studies were multinational, we captured only the number of participants from the African cohort. In this situation as well, we listed all the countries involved but highlighted the African countries in bold typeface. Information not specified in the full-text article or its supplementary data were captured as “NA.” Rural/urban designation of the sample’s origin was only indicated when specified in the article using words such as “rural” (rural), “village” (rural), “city” (urban), and “town” (semi-urban or peri-urban or semi-rural). The Human Microbiome Project (HMP) classification of body sites was used to categorize the sample types. The age range was divided into four categories; young children (0 to 5 years), older children (6 to 12 years), adolescents (13 to 17 years), and adults (18 years and above). We determined the article’s accessibility to African researchers by checking if the paper is designated open-access at the journal website or if the journal itself is open-access or if the paper can be obtained from PubMed Central.

To determine the extent to which the studies focused on diseases of high public health importance in Africa, we analyzed the number of studies that focus on any of the following conditions identified in the World Health Organization [WHO] State of Health in the African Region 2018 Report as being in the top 10 causes of morbidity and mortality in Africa: lower respiratory infections, HIV/AIDS, diarrheal diseases, malaria, preterm birth complications, tuberculosis, neonatal sepsis/infections, stroke, and ischemic heart diseases.

## Results

### Results of the search

**Fig. 1:**
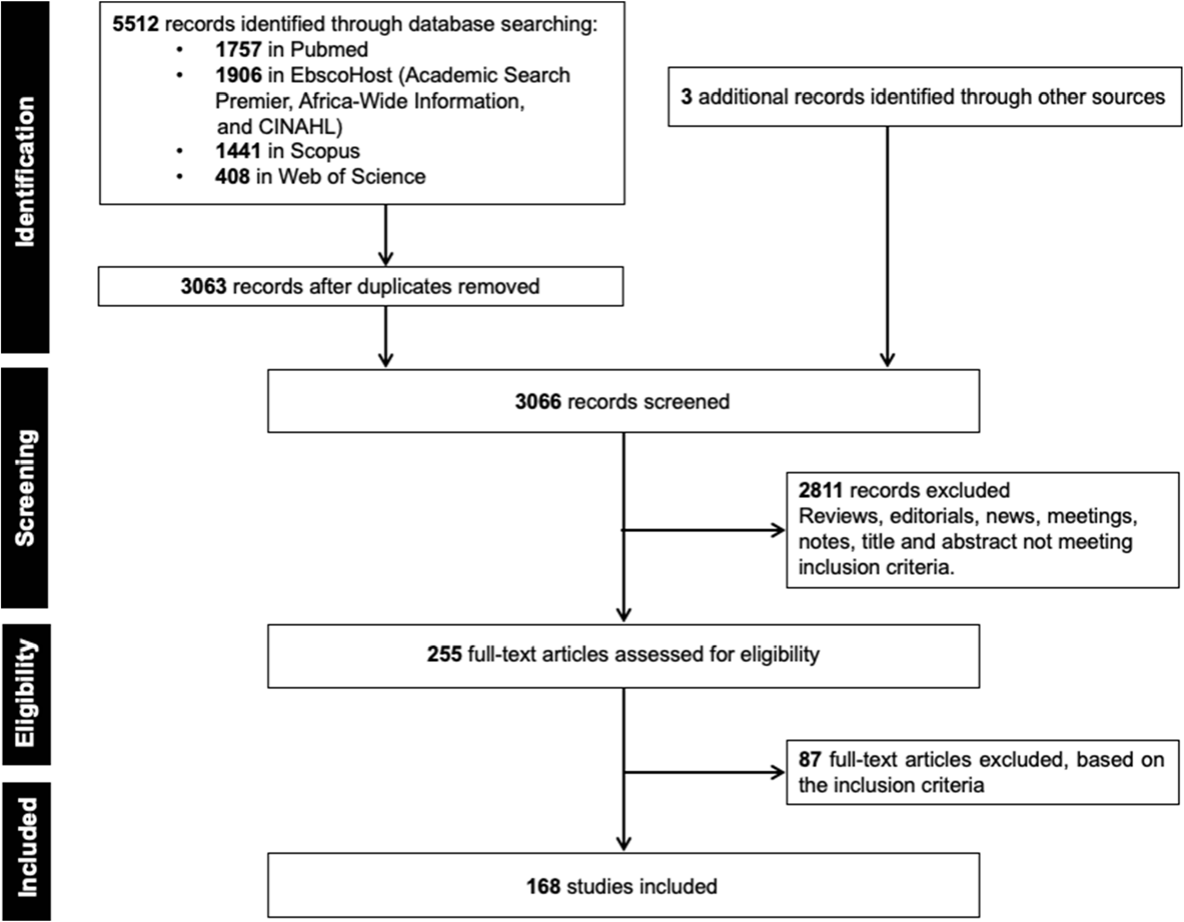
Flow diagram showing the selection of studies according to preferred reporting items for systematic reviews and meta-analysis (PRISMA) guidelines

The search yielded 5515 records (including three articles from additional sources [hand-searching]) with 3066 remaining after removing duplicates. From these records, 2811 were excluded because of ineligibility, and 255 full-text articles were further assessed for eligibility. After a full-text review, a total of 168 eligible human microbiome studies were obtained. Figure 1 shows the PRISMA flowchart summarizing the steps followed in the selection of the final subset of papers used in the analysis.

### Human microbiome research publications in Africa

**Fig. 2:**
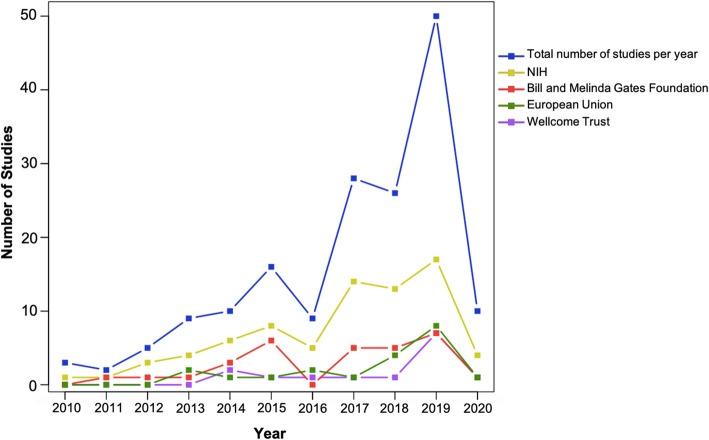
A line plot showing the number of African Human Microbiome research funded by the top four funding agencies over the past 10 years

We found 168 published articles that utilized NGS technology to characterize the human microbiome among African participants. Five broad study designs were used, with cross-sectional studies being the most common (46.4%, 78/168) (Tables 1, 2, and 3). Other designs utilized in the studies were case-control (20.8%, 35/168), randomized control trial (14.3%, 24/168), longitudinal (8.9%, 15/168), and cohort design (8.9%, 15/168). One study involved both longitudinal and cross-sectional designs. The majority of the studies (73.2%, 123/168) involved only one sampling time point. The studies were published in 86 different peer-reviewed journals. The most frequent journal of publication was *PLoS One* (13.1%, 22/168) followed by *Scientific Reports* (4.8%, 8/168), *mBio* (3.6%, 6/168), Microbiome (3.0%, 5/168), and *PLOS Neglected Tropical Diseases* (3.0%, 5/168). More than half of all the studies (67.8%, 114/168) were only published between January 2017 and March 2020 (Fig. 2). A total of 140/168 (83.3%) studies were published as open-access in subscription-based journals or open-access journals or available via PubMed Central and are therefore accessible to researchers based in Africa.

**Table 1:** Summary of the African Human Gut Microbiome studies characteristics

Sample origin (country)	Disease of focus	Sample type	Methods and platform	Scientists involved (affiliation)	Funding source for the study	Study type	Number of participants	Conclusion	Reference
**Gut**									
**Algeria**, **Mali**, **Senegal**, Amazonian French Guiana, France, French Polynesia, Saudi Arabia	Obesity	Stool	16S rRNA, V3–V4 regions, Illumina MiSeq	France, French Polynesia, Mali, Saudi Arabia, Senegal	French Government through the Agence Nationale pour la Recherche (ANR), including the “Programme d’Investissement d’Avenir” under the reference Méditerranée Infection, Région Provence Alpes Côte d’Azur and European funding FEDER PRIMMI (Fonds Européen de Développement Régional - Plateforme de Recherche et d’Innovation Mutualisées Méditerranée Infection)	Case-control	92	High salt levels are associated with alteration of the gut microbial ecosystem and halophilic microbiota, as discovered during this study. Further studies should clarify if the gut microbiota alterations associated with high salt levels and the human halophilic microbiota could be causally related to human disease, such as obesity.	[60]
**Botswana**, **Tanzania**, USA	None	Stool	16S rRNA, V1–V2 regions, Illumina MiSeq	Botswana, Finland, Tanzania, UK, USA	Lewis and Clark Fund, University of Pennsylvania, Leakey Foundation, NIH, National Science Foundation	Cross-sectional	114	Across the cohort, similarity in bacterial presence/absence compositions between people increases with both geographic proximity and genetic relatedness, while abundance weighted bacterial composition varies more significantly with geographic proximity than with genetic relatedness.	[48]
**Burkina Faso**	Diarrhea	Stool	Viral shotgun metagenomic sequencing, 454 pyrosequencing	Burkina Faso, Chile, USA, Vietnam	NIH, Blood Systems Research Institute (USA)	Cross-sectional	98	A potential new genus in the Parvoviridae family was genetically characterized, and a PCR survey showed a prevalence of 4% among the rotavirus antigen-negative cases of childhood diarrhea.	[61]
**Burkina Faso**	None	Rectal swabs	16S rRNA, V3–V4 regions, Illumina	Burkina Faso, Germany, Niger, South Africa, USA	Research to Prevent Blindness, NIH	Randomized clinical trial	62	We found no evidence of an indirect effect of antibiotic use in cohabiting children.	[62]
**Burkina Faso**, Italy	None	Stool	16S rRNA, V5–V6 regions, 454 pyrosequencing	Belgium, Italy	Ministero dell'Universita, e della Ricerca (Italy), Ente Cassa di Risparmio di Firenze, Meyer's Children Hospital	Cross-sectional	15	It is important to sample and preserve microbial biodiversity from regions where effects of globalization on diet are less profound.	[63]
**Burkina Faso**	None	Rectal swabs	16S rRNA, V3–V4 regions, Illumina	Burkina Faso, Germany, South Africa, USA	Research to Prevent Blindness, NIH	Randomized clinical trial	115	Azithromycin affects the composition of the pediatric intestinal microbiome. The effect of amoxicillin and cotrimoxazole on microbiome composition was less clear.	[64]
**Cameroon**	None	Stool	Shotgun metagenomics, Illumina HiSeq	France	ANR MICROREGAL, Centre National de la Recherche Scientifique (LS), Institut Pasteur of Lille	Cross-sectional	57	Our study corroborates and expands prevalence results previously obtained for Blastocystis sp. and provides novel data for Entamoeba spp. and several other protozoan genera. Furthermore, it indicates that multiple protozoa are common residents of the healthy human gut worldwide.	[65]
**Cameroon**	None	Stool	16S rRNA, V5–V6 regions, Illumina MiSeq	France, USA	Howard Hughes Medical Institute, French National Agency for Research	Cross-sectional	64	Results show that gut communities vary significantly with subsistence mode, notably with some taxa previously shown to be enriched in other hunter-gatherer groups (Tanzania and Peru) also discriminating hunter-gatherers from neighboring farming or fishing populations in Cameroon.	[66]
**Cameroon**	Diarrhea	Stool	Viral shotgun metagenomics, Illumina NextSeq	Belgium, Cameroon	KU Leuven grant	Case-control	221	This study showed a huge diversity of highly divergent novel phages, thereby expanding the existing phageome considerably. Further screening of bat viruses in humans or vice versa will elucidate the epidemiological potential threats of animal viruses to human health.	[67]
**Central African Republic**, **Madagascar**	Stunted childhood growth	Gastric, duodenal, and stool	16S rRNA, V4 region, Illumina	Canada, Central African Republic, France, Madagascar	Total Foundation, Institut Pasteur, Pasteur Foundation Switzerland, Nutricia Research Foundation	Case-control	404	Our data suggest that stunting is associated with a microbiome “decompartmentalization” of the gastrointestinal tract characterized by an increased presence of oropharyngeal bacteria from the stomach to the colon, hence challenging the current view of stunting arising solely as a consequence of small intestine overstimulation through recurrent infections by enteric pathogens.	[68]
**Central African Republic**	None	Stool	16S rRNA, V1–V3 regions, 454 pyrosequencing	Central African Republic, USA, Czech Republic	NSF grant, Czech Science Foundation, University of Minnesota College of Biological Sciences, European Social Fund, Czech Republic Government, Central European Institute of Technology, the European Regional Development Fund, the Institute of Vertebrate Biology, Academy of Sciences of the Czech Republic	Cross-sectional	57	The results demonstrate gradients of traditional subsistence patterns in two neighboring African groups and highlight the adaptability of the microbiome in response to host ecology.	[69]
**Central African Republic**	None	Stool	16S rRNA, V1–V3 regions, 454 pyrosequencing	Austria, Central African Republic, Czech Republic, USA	European Social Fund and state budget of the Czech Republic, the U.S. National Science Foundation, Ministry of Education, Youth and Sports of The Czech Republic	Cross-sectional	57	The expanded comparative approach presented here indicates that subsistence patterns, such as those exhibited by contemporary hunter-gatherers or traditional agriculturalists, are associated with gut microbiome composition and diversity characterizing distantly related primates that exploit a broad-based diet.	[70]
**Republic of the Congo**	None	Stool	16S rRNA, V4 region, Shotgun metagenomics, Illumina MiSeq	Republic of the Congo, USA	NIH	Cross-sectional	81	The microbiome of closely related host species may be molded by changes in diet and the degree of antibiotic exposure despite their geographic location.	[71]
**Republic of the Congo, Gabon**	None	Stool and meconium	16S rRNA, V3 region, Ion Torrent PGM	Gabon, France, Republic of the Congo	Centre National de la Recherche Scientifique, Centre International de Recherche Médicales de Franceville, Institut de Recherche pour le Développement, Laboratoire mixte international ZOFAC	Longitudinal	29	Improve our knowledge on the gut bacterial and viral communities of infants from two Sub-Saharan countries during their first month of life.	[72]
**Côte d'Ivoire**	Diarrhea	Stool	Shotgun metagenomic sequencing, Illumina MiSeq	Côte d'Ivoire, Germany, Switzerland	Armasuisse project ARAMIS, the European Union's Seventh Framework Programme	Cross-sectional	4	A metagenomic approach provides detailed information on the presence and diversity of pathogenic organisms in human stool samples.	[73]
**Côte d’Ivoire**	Schistosomiasis	Stool	16S rRNA, V3–V4 regions, Illumina MiSeq	Côte d’Ivoire, Switzerland	European Research Council	Case-control	34	Our study suggests that neither a *S. mansoni* infection nor praziquantel administration triggers a significant effect on the microbial composition and that a higher abundance of Fusobacterium spp., before treatment, is associated with higher efficacy of praziquantel in the treatment of *S. mansoni* infections.	[74]
**Egypt**	Hepatitis C virus	Stool	16S rRNA, V4 region, Illumina MiSeq	Egypt	Not funded by any public or private institution	Case-control	15	This study provides a first overview of major phyla and genera differentiating stage 4-HCV patients from healthy individuals and suggests possible microbiome remodeling in chronic hepatitis C, possibly shaped by bacterial translocation as well as the liver’s impaired role in digestion and protein synthesis.	[75]
**Egypt**	Pediatric cancer	Stool	16S rRNA, V3–V5 regions, Illumina MiSeq	Egypt	Zewail City for Science and Technology	Case-control	5	The study is a start to offer a different angle for personalized treatment progress for pediatric cancer patients, based on the microbial profile rather than following a constant roadmap for the treatment protocol.	[76]
**Egypt**	Obesity and diabetes	Stool	16S rRNA, V3–V4 regions, Illumina MiSeq	Egypt	Not funded by any funding agencies	Case-control	60	The health state of the adults in our study defined the composition of the gut microbiota. Moreover, obesity and diabetes were associated with remarkably enriched populations of Firmicutes and Bacteroidetes.	[77]
**Egypt**, USA	None	Stool	16S rRNA, V4 region and Shotgun metagenomic sequencing, Illumina MiSeq	Egypt, USA	NA	Cross-sectional	28	The differences in fecal microbiota structure and functions and metabolite profiles between Egyptian and US teenagers are consistent with the nutrient variation between Mediterranean and Western diets.	[78]
**Ethiopia**	None	Stool	Viral shotgun metagenomics, Illumina MiSeq	Ethiopia, USA	NIH, That Man May See and The Sara & Evan Williams Foundation, Research to Prevent Blindness, National Heart, Lung, and Blood Institute grant, Blood Systems Research Institute	Cluster randomized trial	269	We documented a difference in pediatric enteric viromes according to mBSFS-C stool consistency category, both in species richness and composition.	[79]
**Ethiopia**	None	Stool	Viral shotgun metagenomics, Illumina MiSeq	Ethiopia, USA	Blood Systems Research Institute, NIH, Sara & Evan Williams Foundation, Bernard Osher Foundation, That Man May See, the Harper Inglis Trust, Bodri Foundation, South Asia Research Fund, Research to Prevent Blindness, Carter Center Ethiopia	Cluster randomized trial	269	Mammalian enteric virome diversity was not reduced in children from villages with a new water well. This population-based sampling also provides a baseline of the enteric viruses present in Northern Ethiopia against which to compare future viromes.	[80]
**Ethiopia**, **Kenya**, **The Gambia**, **Ghana**, Peru, Spain, Sweden, USA	None	Stool	16S rRNA, V1–V3 regions, Illumina MiSeq	Canada, Ethiopia, The Gambia, Ghana, Kenya, Peru, Spain, Sweden, UK, USA	National Science Foundation, NIH	Cross-sectional	217	Our results indicated that household composition (represented by the number of cohabitating siblings and other household members) did not have a measurable impact on the bacterial diversity, evenness, or richness of the IFM. However, we observed that variation in household composition categories did correspond to differential relative abundances of specific taxa, namely Lactobacillus, Clostridium, Enterobacter, and Klebsiella.	[81]
**The Gambia**	None	Stool	16S rRNA, V4 region, Illumina MiSeq	UK, USA	NIH, the Peter J. Shields Endowed Chair in Dairy Food Science	Longitudinal	33	These results suggest that specific types and structures of human milk oligosaccharides (HMOs) are sensitive to environmental conditions, protective of morbidity, predictive of growth, and correlated with specific microbiota.	[82]
**Ghana**, USA	Obesity	Stool	16S rRNA, V4 region, Illumina HiSeq	Ghana, USA	NIH	Case-control	50	We demonstrate that the association between obesity resistance and increased predicted ecological connectivity and stability of the lean Ghanaian microbiota, as well as increased local SCFA receptor level, provides evidence of the importance of a robust gut ecologic network in obesity.	[83]
**Kenya**	Worm infestation	Stool	16S rRNA, V4 region, Illumina MiSeq	Kenya, UK, USA	NIH, Bill and Melinda Gates Foundation	Longitudinal, cross-sectional (case-control)	100	This study contributes to our understanding of how microbial communities differ between soil-transmitted helminths STH-infected and uninfected individuals; the next step will be to understand the impact of the identified differences on human health.	[84]
**Kenya**	None	Stool	16S rRNA, V3–V6 regions, 454 pyrosequencing	Kenya, South Africa, Switzerland, The Netherlands	Eunice Kennedy Shriver National Institute Of Child Health and Human Development, European Union’s Seventh Framework Programme	Double-blind randomized controlled trial	115	In this setting, provision of iron-containing MNPs to weaning infants adversely affects the gut microbiome, increasing pathogen abundance and causing intestinal inflammation.	[85]
**Kenya**	Acute febrile malaria	Stool	16S rRNA, V3–V4 regions, Illumina	Kenya, UK, USA	Wellcome Trust, University of Louisville, NIH	Longitudinal	10	In-depth bioinformatics analysis of stool bacteria has revealed for the first time that human malaria episode/artemether-lumefantrine treatment has minimal effects on gut microbiota in Kenyan infants.	[86]
**Kenya**	None	Stool	16S rRNA, V1, V2, and V3 regions, Illumina MiSeq	Kenya, USA	Bill and Melinda Gates Foundation	Cross-sectional	143	These results suggest that the household should be considered a unit. Livestock activities, health, and microbiome perturbations among an individual child may have implications for other children in the household.	[87]
**Kenya**	None	Vaginal swabs	cpn60 UT-based sequencing, 454 pyrosequencing	Canada, Kenya	NIH, Bill and Melinda Gates Foundation	Cross-sectional	44	Cpn60 UT is ideally suited to next-generation sequencing technologies for further investigation of microbial community dynamics and mucosal community underlying HIV resistance in this cohort.	[88]
**Kenya**	Anemia	Stool	16S rRNA, V3–V4 regions, Illumina MiSeq	Kenya, Switzerland, The Netherlands	ETH Global, the Sawiris Foundation for Social Development, ETH Zurich, DSM Nutritional Products	Double-blind randomized controlled trial	155	A micronutrient powder containing a low dose of highly bioavailable iron reduces anemia, and the addition of galacto-oligosaccharides mitigates most of the adverse effects of iron on the gut microbiome and morbidity in African infants.	[89]
**Kenya**	None	Stool	16S rRNA, V3–V4 regions, Illumina MiSeq	Kenya, The Netherlands, Switzerland	ETH global and the Sawiris Foundation for Social Development, DSM nutritional Products, Sight and life	Double-blind randomized controlled intervention trial	150	Human milk oligosaccharides profile may modulate the infant gut microbiota response to fortificant iron; compared to infants of secretor mothers, infants of non-secretor mothers may be more vulnerable to the adverse effect of iron but also benefit more from the co-provision of GOS.	[9]
**Kenya**	Diarrhea	Stool	16S rRNA, V3–V4 regions, Illumina MiSeq	Kenya, The Netherlands, Switzerland	ETH global and the Sawiris Foundation for Social Development, ETH Zurich, DSM nutritional Products, Sight and life	Double-blind randomized controlled intervention trial	28	Our findings need confirmation in a larger study but suggest that, in African infants, iron fortification modifies the response to broad-spectrum antibiotics: iron may reduce their efficacy against potential enteropathogens, particularly pathogenic *E. coli*, and may increase the risk for diarrhea.	[90]
**Kenya**, **Mali**, **The Gambia**, Bangladesh	Diarrhea	Stool	16S rRNA, V1-V2 region, 454 pyrosequencing	Bangladesh, Kenya, Mali, The Gambia, UK, USA	Bill and Melinda Gates Foundation, NIH, Wellcome Trust	Cross-sectional	786	The study demonstrates that the major differences in the microbiota between diarrheal and normal stools are quantitative differences in the proportions of the most prevalent taxa.	[91]
**Kenya**	None	Stool	16S rRNA, V4 region, Illumina MiSeq	Kenya, USA	International Atomic Energy Agency research, NIH, Colorado Clinical and Translational Sciences Institute	Double-blind, individually-randomized, controlled trial	33	Micronutrient powder fortification over three months in non- or mildly anemic Kenyan infants can potentially alter the gut microbiome. Consistent with previous research, the addition of iron to the MNP may adversely affect the colonization of potential beneficial microbes and attenuate the decrease of potential pathogens.	[92]
**Liberia**, Indonesia	Helminth infections	Stool	16S rRNA, V1-V3 region, Illumina MiSeq (Liberia), 454 pyrosequencing (Indonesia), Shotgun metagenomics, Illumina HiSeq	Indonesia, Liberia, The Netherlands, USA	NIH, Bill and Melinda Gates Foundation	Cross-sectional	98	These results provide a novel insight into the cross-kingdom interactions in the human gut ecosystem by unlocking the microbiome assemblages at taxonomic, genetic, and functional levels so that advances toward key mechanistic studies can be made.	[93]
**Malawi**	None	Stool	16S rRNA, V4 region, Illumina MiSeq	Finland, Malawi, Singapore, USA	Academy of Finland, Bill and Melinda Gates Foundation	Randomized control trial	213	Nutritional supplementation by lipid-based nutrient supplements or corn-soya blend for 12 months did not affect the gut microbiota in the rural Malawian context.	[94]
**Malawi**	Malnutrition	Stool	16S rRNA, V4 region	Finland, Malawi, Singapore, USA	Bill and Melinda Gates Foundation	Randomized, controlled, and partly blinded clinical trial	631	The results do not support the hypothesis that adverse environmental exposures are broadly associated with lower microbiota maturity and diversity but suggest that environmental exposures influence the abundance of several bacterial OTUs and genera and that low maternal education is associated with higher microbiota maturity and diversity.	[95]
**Malawi**	Childhood infections	Stool	16S rRNA, V4 region, Illumina MiSeq	Finland, Malawi, Singapore, USA	Bill and Melinda Gates Foundation	Prospective cohort	631	Our findings generally do not support the hypothesis that morbidity prevalence predicts a subsequent decrease in gut microbiota maturity or diversity in rural Malawian children. Certain morbidity symptoms may be predictive of microbiota maturity and diversity and relative abundances of several bacterial taxa. Furthermore, microbiota diversity and maturity may be associated with the subsequent incidence of fever.	[96]
**Malawi**	Gut inflammation	Stool	16S rRNA, V1–V3, V3–V5 regions, 454 pyrosequencing and Shotgun metagenomic sequencing, Illumina HiSeq	Australia, Malawi, USA	Flinders University, Bill and Melinda Gates Foundation, NIH	Longitudinal	18	The findings do not support the hypothesis that resistant starch reduced gut inflammation in rural Malawian children.	[97]
**Malawi**	Environmental enteric dysfunction	Stool	16S rRNA, V1–V2 regions, Illumina MiSeq	Malawi, USA	The Feed the Future Program, USAID, the Children’s Discovery Institute of Washington University, St. Louis Children’s Hospital	Cross-sectional	81	Bacterial diversity did not vary with the extent of environmental enteric dysfunction.	[98]
**Malawi**	Severe acute malnutrition	Stool	16S rRNA, V4 region, 18S rRNA (28S rRNA variable genetic region 2 and the internal transcribed spacers (transITS)), V4–V5 regions, Illumina MiSeq	Canada, Kenya, Malawi, The Netherlands, USA	Center for Global Child Health, SickKids Research Institute & Natural Sciences and Engineering Research Council of Canada	Cross-sectional	46	We suggest this novel two-amplicon-based strategy will prove an effective tool to deliver new insights into the role of eukaryotic microbiota in health and disease.	[99]
**Malawi**	Malnutrition	Stool	16S rRNA, V4 region, Illumina MiSeq and Shotgun metagenomic sequencing, 454 pyrosequencing	Colombia, Malawi, USA,	Bill and Melinda Gates Foundation, NIH	Case-control	40	The results revealed that apparently healthy twins in discordant pairs have viromes associated with, although not necessarily mediators of severe acute malnutrition.	[100]
**Malawi**	Kwashiorkor	Stool	16S rRNA, V4 region, Illumina and Shotgun metagenomic sequencing, 454 pyrosequencing	Malawi, UK, USA	Bill and Melinda Gates Foundation, NIH	Longitudinal	41	Results illustrate the value of using twins discordant for nutritional phenotypes to characterize the interrelationship between the functional development of the gut microbiome in children and their nutritional status.	[101]
**Malawi**, USA, Venezuela	None	Stool	16S rRNA, V4 region, Illumina HiSeq and Shotgun metagenomic sequencing, 454 pyrosequencing	Malawi, Puerto Rico, USA, Venezuela	NIH, St. Louis Children’s Discovery Institute, Howard Hughes Medical Institute, Crohn’s and Colitis Foundation of America, Bill and Melinda Gates Foundation	Cross-sectional	115	Pronounced differences in bacterial assemblages and functional gene repertoires were noted between US residents and those in Malawi and Venezuela. These distinctive features are evident in early infancy as well as adulthood. The findings underscore the need to consider the microbiome when evaluating human development, nutritional needs, physiological variations, and the impact of westernization.	[102]
**Mali**	Plasmodium falciparum infection	Stool	16S rRNA, V1–V3 regions, 454 pyrosequencing	Mali, USA	NIH	Cross-sectional	200	The findings underscore the diversity of gut microbiota across geographic regions and suggest that strategic modulation of gut microbiota composition could decrease the risk of P. Falciparum infection in malaria-endemic areas, potentially as an adjunct to partially effective malaria vaccines.	[103]
**Mali**	Blastocystis	Stool	16S rRNA, V3–V4 regions, Illumina MiSeq	France, Mali	The IHU-Mediterranean Infection Foundation, African Academy of Sciences, Wellcome Trust, UK government	Cross-sectional	296	Blastocystis colonization is significantly associated with a higher diversity of the gut bacterial communities in healthy children, while it is not associated with the presence of potentially pathogenic bacteria in the human gut.	[104]
**Mali**, **Mozambique**, India	Shigella infections	Stool	Shotgun metagenomics, Illumina HiSeq	India, Mali, Mozambique, Pakistan, USA	Bill and Melinda Gates Foundation, NIH, Fogarty International Center	Cross-sectional	18	Metagenomic sequencing indicates that *Shigella*/EIEC qPCR-positive samples are similar to those of *Shigella* culture-positive samples in *Shigella* sequence composition, thus supporting qPCR as an accurate method for detecting *Shigella*.	[105]
**Mali**	Pulmonary tuberculosis	Stool	Shotgun metagenomic sequencing, Illumina HiSeq	Mali, USA	NIH, Howard Hughes Medical Institute	Cross-sectional	10	Oral Urea Breath Test has significant limitations as a point of care diagnostic tool for pulmonary tuberculosis in a setting with endemic *H. pylori* infection.	[106]
**Morocco**	Colorectal cancer	Stool	16S rRNA, V1–V2 regions, Illumina MiSeq	Morocco, USA	NIH	Case-control	23	This suggests that involvement of the functional metagenomes detected in the study is similar and relevant in the carcinogenesis process, independent of the origin of the samples. Results from this study allowed identification of bacterial taxa relevant to the Moroccan population and encourages larger studies to facilitate population-directed therapeutic approaches.	[3]
**Niger**	None	Rectal swabs	16S rDNA, V3–V4 regions, Illumina	Niger, USA	Bill and Melinda Gates Foundation, Peierls Foundation, NIH, Research to Prevent Blindness	Double-blind randomized controlled trial	80	Oral administration of azithromycin definitively decreases the diversity of the gut microbiome of children in an antibiotic-naive community.	[107]
**Niger**	None	Rectal swabs and Stool	Shotgun metagenomics, Illumina HiSeq	Niger, USA	Bill and Melinda Gates Foundation, NIH, Research to Prevent Blindness Career Development Award, Research to Prevent Blindness	Double-masked, cluster randomized controlled clinical trial	300	Two mass azithromycin administrations, 6 months apart, in preschool children led to long-term alterations of the gut microbiome structure and community diversity. Here, long-term microbial alterations in the community did not imply disease but were associated with an improvement in childhood mortality.	[108]
**Niger**	None	Rectal swabs	Shotgun metagenomics, Illumina HiSeq	Niger, USA	Bill and Melinda Gates Foundation, the Peierls Foundation, Research to Prevent Blindness Career Development Award, Research to Prevent Blindness	Cluster randomized controlled trial	300	These results suggest that prolonged mass azithromycin distribution to reduce childhood mortality reduces certain gut bacteria, including known pathogens, while selecting for antibiotic resistance.	[109]
**Niger**	None	Rectal swabs	16S rRNA, V3–V4 regions, Illumina MiSeq	Niger, USA	Bill and Melinda Gates Foundation, Peierls Foundation, NIH, Research to Prevent Blindness	Cluster randomized clinical trial	80	Pooling microbiome samples before DNA amplification and metagenomics sequencing to estimate community-level diversity is a viable measure to consider in population-level association research studies.	[110]
**Niger**, **Senegal**	Kwashiorkor	Stool	16S rRNA, V3–V4 regions, Illumina MiSeq, and MALDI-TOF Culturomics	France, Mali, Niger, Senegal, UK	The Mediterranée Infection Foundation	Case-control	15	A complex of 12 species identified only in healthy children using culturomics and metagenomics were identified as probiotic candidates, providing a possible, defined, reproducible, safe, and convenient alternative to fecal transplantation to restore a healthy gut microbiota in malnourished children. Microbiotherapy based on selected strains has the potential to improve the current treatment of severe acute malnutrition and prevent relapse and death by reestablishing a healthy gut microbiota.	[111]
**Nigeria**	Type 2 diabetes	Stool	16S rRNA, V4 region, Illumina MiSeq	Nigeria, USA	NIH, the Intramural Research Program of the Center for Research on Genomics and Global Health with funding from NHGRI and NIDDK	Case-control	291	This first investigation of gut microbiome and diabetes in urban Africans shows that type 2 diabetes is associated with compositional changes in gut microbiota highlighting the possibility of developing strategies to improve glucose control by modifying bacterial composition in the gut.	[112]
**Nigeria**	None	Stool	16S rRNA, V4 region, Illumina MiSeq	Austria, Nigeria	Austrian Agency for International Mobility and Cooperation in Education, Science, and Research, Centre for International Cooperation and Mobility	Case-control	50	Significant differences in composition between both groups were likely due to differences in diet and lifestyle and exposure to pathogens. These results suggest that microbial diversity may not always be higher in non-industrialized societies than in westernized societies, as previously assumed.	[113]
**Nigeria**	None	Stool	16S rRNA, V3–V4 regions, Illumina MiSeq	Austria, Italy, Nigeria	Society for Applied Microbiology	Cross-sectional	48	Our findings stress the loss of ancient signatures along with urbanization and support distinct trajectories of development of the intestinal ecosystem in early life, depending on human subsistence.	[114]
**Nigeria**, **Sudan**, Azerbaijan, Jordan	Diabetes	Stool	16S rRNA, V4 region, Illumina MiSeq	Azerbaijan, Czech Republic, Jordan, Nigeria, Sudan	Ministry of Health of the Czech Republic	Case-control	83	Based on our results, some type of distortion of the gut bacteriome appears to be a global feature of type 1 diabetes, and our findings for four distant populations add new candidates to the existing list of bacteria. It remains to be established whether the observed associations are markers or causative factors.	[115]
**Nigeria**	HIV/AIDS	Rectal swabs	16S rRNA, V3–V4 regions, Illumina MiSeq	Nigeria, USA	Mpower, NIH, US Military HIV Research Program, CDC, Global AIDS program with IHVN	Cross-sectional	130	Untreated HIV infection does not significantly alter the rectal microbiota, whereas prior treatment is associated with a shift toward a more pathogenic pattern of microbiota.	[116]
**Nigeria**	Human papillomavirus and HIV	Rectal swabs	16S rRNA, V4 region, Illumina MiSeq	Nigeria, USA	National Cancer Institute, NIH, Henry M. Jackson Foundation for the Advancement of Military Medicine, U.S. Department of Defense, Fogarty Epidemiology Research Training for Public Health Impact in Nigeria program, the President’s Emergency Plan for AIDS Relief through a cooperative agreement between the Department of Health and Human Services/Centers for Disease Control and Prevention, Global AIDS Program, Institute for Human Virology-Nigeria	Cross-sectional	113	Further studies are needed to evaluate whether an anal microbial community enriched with members of the Fusobacteria phylum is associated with HIV-infected MSM who are virally suppressed and have a concurrent HPV-16.	[117]
**Senegal**	None	Stool	16S rRNA, V6 region, 454 pyrosequencing	France	European Research Council	Cross-sectional	1	There is evidence of the presence of mimiviruses and marseilleviruses in humans.	[118]
**Senegal**, France	None	Stool	16S rRNA, V6 region, 454 pyrosequencing	France, Senegal, USA	Centre National de la Recherche Scientifique, Institut de Recherche et Développement, Aix-Marseille Université	Cross-sectional	2	Microbial diversity in the human gut is substantially broader than predicted on the basis of genomic and metagenomic analyses.	[119]
**South Africa**	Respiratory, gastrointestinal, and other diseases	Stool	16S rRNA, V3–V4 regions, Illumina Miseq	Germany, South Africa	Institute for Food, Nutrition and Well-being and Genomics Research Institute, University of Pretoria	Case-control	34	This study provides preliminary evidence for the fecal microbiome-derived dysbiosis signature and pathobiome concept that may be observed in young children during illness.	[120]
**South Africa**	Atopic dermatitis	Stool	16S rRNA, V4 region, Illumina MiSeq	South Africa, USA	NIH, Brinson Foundation	Cross-sectional	38	No significant differences were observed in microbial diversity between the children with atopic dermatitis (AD) and the control children, and there were no differences in the relative abundance for any taxa between these 2 groups after adjusting for multiple comparisons.	[121]
**South Africa**, USA	Colorectal cancer	Stool	16S rRNA, V4 region, Illumina MiSeq	Germany, South Africa, UK, USA	NIH	Prospective cohort	21	The low-fiber, high-fat diet of Alaskan Native people and exposure to carcinogens derived from diet or environment are associated with a tumor-promoting colonic milieu as reflected by the high rates of adenomatous polyps in Alaska Native participants.	[122]
**South Africa**, USA	Colon cancer risk	Stool	16S rRNA, 454 pyrosequencing	South Africa, The Netherlands, USA	NIH	Cross-sectional	12	The results support the hypothesis that colon cancer risk is influenced by the balance between microbial production of health-promoting metabolites such as butyrate and potentially carcinogenic metabolites such as secondary bile acids.	[123]
**South Africa**	None	Stool	16S rRNA, V4 region, Illumina MiSeq	South Africa, USA	National Institute of Child Health and Human Development, National Institute of Environmental Health Sciences, National Science Foundation, University of Washington Center for AIDS Research, NIH, Harry Crossley Foundation, Suid-Afrikaanse Akademie vir Wetenskap en Kuns, US Agency for International Development	Prospective, longitudinal	155	These data suggest that non-exclusive breastfeeding alters the gut microbiota, increasing T-cell activation and, potentially, mucosal recruitment of HIV target cells. Study findings highlight a biologically plausible mechanistic explanation for the reduced post-natal HIV transmission observed in exclusively breastfed infants.	[124]
**South Africa**	None	Stool	16S rRNA, V4 region, Illumina MiSeq	South Africa, USA	H3Africa U01 award from NIH, the Wellcome Trust, Bill and Melinda Gates Foundation Global Health Grant, the National Research Foundation, and the Carnegie Corporation of New York, South African Medical Research Council	Cohort	197	The meconium from infants investigated in our study contained high proportions of the phylum Proteobacteria, in particular bacteria within the Enterobacteriaceae family.	[125]
**Tanzania**, Italy	None	Stool	Shotgun metagenomic sequencing	Germany, Italy, USA	Max Planck-Gessellschaft, Lincy Foundation	Cross-sectional	27	The results demonstrate how the functional specificity of the gut microbiota shows correlation to some environmental and lifestyle factors specific to the Hadza and urban Italians sampled in this study.	[32]
**Tanzania**, Italy	None	Stool	16S rDNA, V4 region, 454 pyrosequencing	Germany, Italy, Tanzania, UK, USA	Lincy Foundation, Max-Planck-Gesellschaft	Cross-sectional	27	The Hadza have higher levels of microbial richness and biodiversity than Italian urban controls.	[33]
**Tanzania**, USA	None	Stool	16S rRNA, V1–V3 and V3–V5 regions, Illumina MiSeq and Shotgun metagenomic sequencing, Illumina HiSeq	Canada, Tanzania, UK, USA	The Emch Family Foundation and Forrest & Frances Lattner Foundation, C&D Research Fund, NIH, Discovery Innovation Fund Awards	Longitudinal	188	The taxa within the Hadza that are the most seasonally volatile similarly differentiate industrialized and traditional populations. These data indicate that some dynamic lineages of microbes have decreased in prevalence and abundance in modernized populations.	[126]
**Tanzania**	Toxic blood metal levels	Stool	16S rRNA, V6 region, Ion Torrent PGM	Canada, Tanzania	Bill and Melinda Gates Foundation	Randomized open-label pilot study	104	The study demonstrated the potential value of long-term probiotic-based interventions to counter mercury and arsenic exposure in vulnerable populations.	[21]
**Uganda**	HIV/AIDS	Stool	16S rRNA, Illumina MiSeq	Uganda, USA	NIH, Harvard Center for AIDS Research	Case-control	122	Severe immunodeficiency is the likely mechanism leading to changes in the fecal microbiome.	[127]
**Uganda**	Malnutrition	Stool	16S rRNA, V3–V4 regions, Illumina MiSeq	Denmark, Uganda	Knud Højgaards Foundation, Oticon Foundation, Arvid Nilssons Foundation, Aase and Ejnar Danielsens Foundation, Brødrene Hartsmanns Foundation, Augustinus Foundation, Axel Muudfeldts Foundation, Torkild Steenbecks Legat, The Danish Free Research Council	Cross-sectional	87	The non-edematous SAM children have lower gut microbiota diversity compared to edematous SAM children; however, no clear compositional differences were identified.	[128]
**Uganda**	None	Stool	16S rRNA, V1–V2 regions, Illumina MiSeq	Uganda, UK	Wellcome, European Research Council Starting Grant	Cross-sectional	3	Stool collected in a fieldwork setting for comparative microbiome analyses should ideally be stored as consistently as possible using the same preservation method throughout.	[129]
**Uganda**	Malnutrition	Stool	16S rRNA	The Netherlands, Norway, South Africa, Uganda	The Throne Holst Foundation, University of Oslo, TNO’s Early Research Program “Personalized Health”	Two-armed, open-cluster, randomized education intervention	147	The maternal education intervention had positive effects on child development and growth at 3 years but did not alter gut microbiota composition. This intervention may be applicable in other low-resource settings.	[130]
**Uganda**	None	Stool	16S rRNA, V4–V5 regions, 454 pyrosequencing	Uganda, UK	UK Medical Research Council	Cross-sectional	21	The results show potential for the sharing of usually commensal bacterial taxa between humans and other animals.	[131]
**Zimbabwe**	HIV	Rectal swabs	16S rRNA, V4 region, Illumina MiSeq	Australia, Norway, South Africa, UK, Zimbabwe	Global Health and Vaccination Programme of the Medical Research Council of Norway, Northern Norway Regional Health Authority, NIH	Case-control	280	Human immunodeficiency virus-infected children have altered gut microbiota. Prolonged antiretroviral therapy may restore the richness of the microbiota closer to that of HIV-uninfected children.	[132]
**Zimbabwe**	HIV	Stool	Shotgun metagenomics	Canada, Uganda, UK, Zimbabwe	Wellcome Trust, Canadian Institutes of Health Research, Medical Research Council, European Union, MRC Clinical Trials Unit at UCL	Randomized control study	72	These data demonstrate that cotrimoxazole reduces systemic and intestinal inflammation both indirectly via antibiotic effects on the microbiome, and directly by blunting immune and epithelial cell activation. Synergy between these pathways may explain the clinical benefits of cotrimoxazole despite high antimicrobial resistance, providing further rationale for extending coverage among people living with HIV in sub-Saharan Africa.	[133]
**Zimbabwe**	Parasite infection	Stool	16S rRNA, V3–V4 regions, Illumina MiSeq	UK, Zimbabwe	WHO, Wellcome Trust, Thrasher Research Fund, Waiwick Medical School	Longitudinal	62	There are significant differences in the gut microbiome structure of infected vs. uninfected children and the differences were refractory to Praziquantel treatment.	[134]

**Table 2:** Summary of the African Human Urogenital Microbiome studies characteristics

Sample origin (country)	Disease of focus	Sample type	Methods and platform	Scientists involved (affiliation)	Funding source for the study	Study type	Number of participants	Conclusion	Reference
**Urogenital**									
**Burkina Faso**	HIV	Cervicovaginal lavage	16S rRNA, 454 pyrosequencing	Burkina Faso, France, UK, USA	NIH, Agence Nationale de Recherche sur le Sida, the Veterans Affairs Research Service, the Mucosal and Vaccine Research Program Colorado	Nested case-cohort study	64	The data suggests that alterations in vaginal microbial communities are associated with an increased risk for perinatal MTCT.	[135]
**Kenya**	Genital ulcer disease	Genital ulcer specimens	16S rRNA, V1-V2 region, 454 pyrosequencing	Canada, USA	Chicago Development Center for AIDS Research	Cross-sectional	59	Anaerobic bacteria are more common in genital ulcers of uncircumcised men.	[136]
**Kenya**	HIV-1	Vaginal swabs	16S rRNA, V1-V3 region, 454 pyrosequencing	Kenya, USA	Bill and Melinda Gates Foundation Grand Challenges Explorations, NIH, the Gilead Foundation grant	Longitudinal	72	Group counseling is effective in reducing intravaginal practices, and this in turn improved the vaginal health.	[137]
**Kenya**	None	Cervicovaginal lavage	16S rRNA, V3 region, Illumina MiSeq	Canada, Kenya	CIHR, Grand Challenges Canada, The Ontario HIV Treatment Network	Cross-sectional	67	High-risk sexual behavior is associated with greater diversity of the vaginal microbiota and lack of *Lactobacillus* species.	[138]
**Kenya**	HIV	Vaginal swabs	16S rRNA, V3 region, Illumina MiSeq	Canada, Kenya	Canadian Institutes of Health Research (CIHR)	Cohort	58	MPA-induced hypoestrogenism may alter key metabolic components that are necessary for vaginal colonization by certain bacterial species including lactobacilli and allow for greater bacterial diversity in the vaginal microbiota.	[139]
**Kenya**	*Trichomonas vaginalis* or *Chlamydia trachomatis* infections in pregnancy	Vaginal swabs	16S rRNA, V2 V4 V8 regions, Ion Torrent PGM	Belgium, Kenya, UK	Not specified	Case-control	53	The vaginal microbiomes of TV and CT-infected women were markedly different from each other and from women without TV and CT. Future studies should determine whether the altered microbiomes are merely markers of disease, or whether they actively contribute to the pathology of the two genital infections.	[140]
**Kenya**, **Tanzania**, **Uganda**	HIV	Vaginal swabs	16S rRNA, V3–V4 regions, 454 pyrosequencing	Kenya, USA	NIH	Nested case-control	110	Vaginal microbiota could influence women’s risk of HIV acquisition at multiple levels.	[141]
**Kenya**	HIV	Semen	16S rRNA, V3–V4 regions, 454 pyrosequencing	Kenya, UK, USA	NIH, the University of Washington Center for AIDS Research, the KEMRI-Wellcome Trust Research Programme at the Centre for Geographic Medicine Research-Kilifi	Cross-sectional	13	Most of these HIV-1-infected men had bacteria in their semen. Antiretroviral therapy use was associated with undetectable semen HIV-1 RNA and lower semen bacterial concentrations, whereas insertive anal sex was associated with higher bacterial concentrations.	[142]
**Kenya**	Bacterial vaginosis	Cervicovaginal lavage	16S rRNA, V3–V4 regions, Illumina HiSeq	Canada, Kenya, USA	Canadian Institutes of Health Research, Ontario HIV Treatment Network, NIH	Observational prospective cohort design	45	Bacterial Vaginosis treatment reduced genital CD4+ T-cell HIV susceptibility and IL-1 levels, but dramatically increased the genital chemokines that may enhance HIV susceptibility; the latter effect was related to the restoration of a *Lactobacillus* inner–dominated microbiota. Further studies are needed before treatment of asymptomatic Bacterial Vaginosis can be recommended for HIV prevention in women from African, Caribbean, and other Black (ACB) communities.	[143]
**Nigeria**	High-risk human papillomavirus infection	Mid-vaginal swabs	16S rRNA, V4 region, Illumina MiSeq	Nigeria, UK, USA	NIH	Cross-sectional	278	Vaginal microbial composition in African women is similar to that of African American women. Also, hrHPV infection was strongly associated with the abundance of various vaginal bacterial taxa.	[144]
**Nigeria**	High-risk human papillomavirus infection	Mid-vaginal swabs	16S rRNA, V3–V4 regions, Illumina MiSeq	Nigeria, USA	NIH	Longitudinal	194	A significant association between persistent *M. hominis* in the vaginal microbiota and persistent hrHPV in this study, but reverse causation could not rule out.	[145]
**Nigeria**	Schistosomiasis infection and bladder pathology	Urine	16S rRNA, V3 region, Ion Torrent PGM	India, Nigeria, USA	NA	Cross-sectional	70	The urinary microbiome is a factor to be considered in developing biomarkers, diagnostic tools, and new treatment for urogenital schistosomiasis and induced bladder pathologies.	[146]
**Rwanda**	Bacterial vaginosis	Vaginal swabs	16S rRNA, V3–V4 regions, Illumina HiSeq	The Netherlands, Rwanda, UK	DFID/MRC/Welcome Trust Joint Global Health Trials Scheme as a Development Project, University of Liverpool	Prospective cohort	68	Metronidazole alone may not cure women with high *G. vaginalis* relative abundance, potentially due to biofilm presence, and women with high pathobionts concentration. These women may benefit from additional biofilm-disrupting and/or pathobiont-targeting treatments.	[147]
**Rwanda**, USA	HIV	Cervicovaginal lavage	16S rRNA, 454 multitag pyrosequencing	Rwanda, USA	NIH, the Chicago Developmental Center for AIDS Research	Case-control	40	Similar prevalence of most major bacterial genera and *Lactobacillus* species in Rwanda and USA women.	[148]
**Rwanda**	Bacterial vaginosis	Vaginal swabs	16S rRNA, V6 region, Illumina MiSeq	Canada, Rwanda	Canadian International Development Agency, CIHR	Cross-sectional	131	Differences in the vaginal metabolome are driven by bacterial diversity.	[31]
**Rwanda**	None	Vaginal swabs	16S rRNA, V6 region, Illumina MiSeq	Canada, Rwanda, USA	Canadian Institute Health Research Vogue Team Grant	Randomized, blinded, placebo-controlled clinical trial	13	Overall women were receptive to the probiotic concept, but the lack of information on such products and logistical and economical challenges pose problems for wider population engagement.	[149]
**South Africa**	HIV, Papillomavirus infection and cervical cancer	Vaginal swabs	16S rRNA, V3–V4 regions, Illumina HiSeq	France, The Netherlands, South Africa, UK	The European Commission 7th Framework Programme, University of Liverpool	Nested case-control	448	hrHPV infection (and/or increased sexual risk-taking) may cause anaerobic vaginal dysbiosis, but a bidirectional relationship is also possible. In this population, dysbiosis did not increase CIN2þ risk, but CIN2þ increased dysbiosis risk. The CIN2þ risk associated with progestin-only injectable use requires further evaluation.	[147]
**South Africa**	Papillomavirus infection	Cervical swabs	16S rRNA, V3–V4 regions, Illumina MiSeq	South Africa	National Research Foundation of South Africa, Poliomyelitis Research Foundation (PRF), Cancer Association of South Africa (CANSA), University of Cape Town (UCT) Research Incentive Scheme, UCT Cancer Research Initiative	Cross-sectional	87	A majority of the reproductive-age HIV-seronegative Black South African women (57%) had cervical microbiota not dominated by Lactobacillus, the bacteria assumed to constitute a healthy cervical microbiota. These cervical microbiota were associated with findings suggestive of bacterial vaginosis.	[150]
**South Africa**	None	Cervical swabs	16S rRNA, V4 region, Ion Torrent PGM	South Africa	National Research Foundation (NRF) of South Africa, Poliomyelitis Research Foundation (PRF), Cancer Association of South Africa (CANSA), University of Cape Town (UCT) Research Incentive Scheme, UCT Cancer Research Initiative	Retrospective cross-sectional	62	To date, this remains the first study to examine the association between prevalent HPV and cervical microbiota in a Black South African cohort. Further investigations into the role of the cervical and vaginal microbiome in HPV/HR-HPV infections are warranted.	[151]
**South Africa**, Australia, China	Prostate cancer	Prostate tumor samples	Shotgun metagenomics, Illumina HiSeq	Australia, Canada, China, South Africa	Cancer Association of South Africa, China Scholarship Council, University of Sydney Foundation, Australian Prostate Cancer Research Centre, New South Wales	Cross-sectional	6	Our study provides suggestive evidence for the presence of a core, bacteria‐rich, prostate microbiome. While unable to exclude fecal contamination, the observed increased bacterial content and richness within the African vs non-African samples, together with elevated tumor mutational burden, suggests the possibility that bacterially driven oncogenic transformation within the prostate microenvironment may be contributing to aggressive disease presentation in Africa.	[152]
**South Africa**	*Chlamydia trachomatis* infection	Vulvo-vaginal, vaginal lateral wall, and endocervical swabs	16S rRNA, V4 region, Illumina MiSeq	Australia, South Africa, USA	European and Developing Countries Clinical Trials Partnership (EDCTP) Strategic Primer grant, South African Department of Science and Technology	Cohort	72	In this African adolescent cohort, significant differences between the lateral vaginal wall and endocervical microbiota diversity and composition were evident, although neither were strongly associated with *Chlamydia trachomatis* infection.	[153]
**South Africa**	None	Cervical swabs	16S rRNA, V4 region and Shotgun metagenomic sequencing, Illumina MiSeq	South Africa, USA	Bill and Melinda Gates Foundation, IAVI, NIH	Prospective cohort	146	The bacterial microbiome plays a role in modulating HIV risk, genital microbiome can significantly alter host inflammation.	[154]
**South Africa**	Bacterial vaginosis and sexually transmitted infections	Vulvo-vaginal swabs	16S rRNA, V4 region, Illumina MiSeq	South Africa, USA	European and Developing Countries Clinical Trials Partnership, the South African Department of Science and Technology	Cross-sectional	102	Young 16–22-year-old women in under-resourced Cape Town community have a high incidence of STIs, particularly chlamydia and high-risk HPV, as well as Bacterial vaginosis. The high abundance of *Prevotella amnii* may increase HIV risk, given its inflammatory capacity. Laboratory-based testing for STIs (chlamydia and gonorrhea in particular) appear to be warranted in this community, together with further monitoring or treatment of BV.	[23]
**South Africa**	Bacterial vaginosis	Vulvo-vaginal swabs	16S rRNA, V4 region, Illumina MiSeq	Australia, South Africa, USA	European and Developing Countries Clinical Trials Partnership (EDCTP), South African Department of Science and Technology	Cohort	168	We propose that women with this BVAB1-dominated subtype may have chronic genital inflammation due to persistent BV, which may place them at a particularly high risk for HIV infection.	[24]
**South Africa**	Bacterial vaginosis	Vaginal swabs	16S rRNA, V4 region, Illumina MiSeq	Australia, South Africa, USA	European and Developing Countries Clinical Trials Partnership (EDCTP) Strategic Primer grant, South African Department of Science and Technology	Cohort	181	Our results therefore suggest that HIV prophylactic approaches targeting the vaginal microbiota should be geographically tailored.	[25]
**South Africa**	HIV	Cervicovaginal lavage	16S rRNA, V3–V4 regions, Illumina MiSeq	Canada, South Africa, Sweden, USA	CIHR, the Department of Pharmaceutics at the University of Washington, the Public Health Agency of Canada	Clinical trial	688	This study provides evidence linking vaginal bacteria to microbicide efficacy through tenofovir depletion via bacterial metabolism.	[155]
**South Africa**	HIV	Cervical swabs	16S rRNA, V4 region, Illumina MiSeq	South Africa, USA	Bill and Melinda Gates Foundation, IAVI, NIH, the Harvard Center for AIDS Research	Prospective study	236	The results suggest that highly prevalent genital bacteria increase HIV risk by inducing mucosal HIV target cells. These findings may be leveraged to reduce HIV acquisition in women living in sub-Saharan Africa.	[156]
**Tanzania**	Cervical cancer and HIV	Cervical swabs	16S rRNA, V4 region, Illumina MiSeq	Tanzania, USA	NA	Cross-sectional	144	These results suggest a greater influence of the bacterial microbiota on the outcome of HPV infection than previously thought.	[157]
**Tanzania**	HIV	Vaginal swabs	16S rRNA, V6 region, Illumina	Canada, Tanzania, The Netherlands	Natural Sciences and Engineering Research Council of Canada	Longitudinal	132	The vaginal microbiota among women living with HIV in Sub-Saharan Africa constitutes several profiles associated with a normal microbiota or BV.	[30]
**Uganda**	HIV	Coronal sulcus swabs	16S rRNA, V3–V6 regions, 454 pyrosequencing	Uganda, USA	NIH	Randomized control trial	156	Combining bacterial quantification with parallel sequencing showed that circumcision resulted in significant decreases in the absolute abundances of several anaerobic bacterial taxa that defined the uncircumcised penis microbiome.	[26]
**Uganda**	HIV	Coronal sulcus swabs	16S rRNA, V3–V6 regions, 454 pyrosequencing	Canada, Uganda, USA	NIH, Bill and Melinda Gates Foundation, the Doris Duke Charitable Foundation	Cross-sectional	165	Female partner Nugent bacterial vaginosis is significantly associated with penile microbiota. The data support the exchange of bacterial vaginosis-associated bacteria through intercourse, which may explain BV recurrence and persistence.	[27]
**Uganda**	Genital anaerobic bacterial overgrowth	Subpreputial swabs	16S rRNA, V3–V6 regions, 454 pyrosequencing	Uganda, USA	NIH	Case-control	147	The PrePex-associated increase in anaerobes may account for unpleasant odor and a possible heightened risk of tetanus.	[28]
**Uganda**	HIV	Coronal sulcus swabs	16S rRNA, V3–V4 regions, Illumina MiSeq	Canada, Uganda, USA	NIH, Bill and Melinda Gates Foundation, CIHR	Case-control	182	Penile anaerobes may be a sexually transmissible risk factor for HIV and modifying the penile microbiome could potentially reduce HIV acquisition in both men and women.	[29]
**Uganda**	None	Coronal sulcus swabs	16S rRNA, V3–V4 regions, 454 pyrosequencing	Uganda, USA	NIH, Translational Genomics Research Institute	Randomized control trial	12	The reduction in putative anaerobic bacteria after circumcision may play a role in protection from HIV and other sexually transmitted diseases.	[158]
**Uganda**	HIV	Vaginal swabs	16S rRNA, V3–V4 regions	Canada, Uganda, USA	NIH	Double-blind randomized placebo-controlled trial	92	The vaginal microbiome of HIV-infected women was not affected by the initiation of ART or immune reconstitution in this observational study. Further research is needed to explore the long-term effects of ART treatment on the vaginal microbiome.	[159]
**Zambia**	HIV	Vaginal swabs	Shotgun metagenomics, Illumina HiSeq	USA, Zambia	Global Alliance to Prevent Prematurity and Stillbirth and the Center for AIDS Research, NIH	Cohort	256	Pregnant women in Zambia, particularly those with HIV, had diverse anaerobe-dominant vaginal microbiota.	[160]
**Zimbabwe**	HIV	Vaginal swabs	16S rRNA, V4 region, Illumina MiSeq	South Africa, USA, Zimbabwe	Letten Foundation Norway	Cross-sectional	356	Pregnant women living with HIV have more diverse vaginal communities and altered community structure compared to pregnant uninfected women. However, preterm birth was associated with HIV infections independent of vaginal community state type.	[161]

**Table 3:** Summary of the African Human Microbiome studies characteristics (other body sites)

**Sample origin (country)**	**Disease of focus**	**Sample type**	**Body site**	**Methods and platform**	**Scientists involved (affiliation)**	**Funding source for the study**	**Study type**	**Number of participants**	**Conclusion**	**Reference**
**Eye**										
**Gambia**	Trachoma	Ocular swabs	Eye	16S rRNA, V1-V3 region, Illumina MiSeq, and 454 pyrosequencing	The Gambia, UK	The Wellcome Trust	Case-control	361	Comparisons between active and scarring trachoma supported the relative absence of type-2 interferon responses in scarring, whilst highlighting a common suppression of re-epithelialization with altered epithelial and bacterial adhesion, likely contributing to development of scarring pathology.	[162]
**The Gambia**	Trachomatous disease	Conjunctival swabs	Eye	16S rRNA, V1–V3 regions, 454 pyrosequencing	The Gambia, UK, USA	Wellcome Trust, NIH	Case-control	220	The results indicate that changes in the conjunctival microbiome occur in trachomatous disease however, whether these are a cause or a consequence is not yet known.	[163]
**Anterior nares and naso- and oropharynx**										
**Botswana**	Respiratory infections	Nasopharyngeal swabs	Nasopharynx	16S rRNA, V3 region, Illumina MiSeq	Botswana, Canada, USA	Thrasher Research Fund, Children’s Hospital of Philadelphia, Pincus Family Foundation, NIH, CIPHER grant, the International AIDS Society, supported by ViiV Healthcare	Case-control	319	Pneumonia and upper respiratory infection symptoms are associated with distinct nasopharyngeal microbiota biotypes in African children. A lower abundance of the commensal genus Dolosigranulum may contribute to the higher pneumonia risk of HIV-infected children.	[164]
**Botswana**	Pneumococcal infections	Nasopharyngeal swabs	Nasopharynx	16S rRNA, V3 region, Illumina MiSeq	Botswana, Canada, USA	Thrasher Research Fund, Pincus Family Foundation, NIH, ViiV Healthcare	Case-control	170	Pneumococcal colonization was associated with characteristic alterations of the nasopharyngeal microbiota of children that reflect synergistic and antagonist interactions of S. pneumoniae with commensal bacteria and other potential respiratory pathogens.	[165]
**Kenya**	None	Nasopharyngeal specimens	Nasopharynx	16S rRNA, 454 pyrosequencing	Kenya, UK, USA	GlaxoSmithKline Biologicals	Double-blind randomized controlled trial	60	Vaccination of children with two doses of PHiD-CV did not significantly alter the nasopharyngeal microbiome.	[166]
**Egypt**	None	Nasal swabs	Nose	16S rRNA, V3–V4 regions, Illumina MiSeq	Egypt	Not funded by any funding agencies	Case-control	19	Rural communities displayed higher diversity than that was found across industrial populations that may be attributed to reduced exposure to environmental pollution found in the industrial cities.	[167]
**Gabon**, Germany	None	Anterior nare swabs	Nose	16S rRNA, V1-V2 region, Illumina GAIIx Genome Analyzer	Germany	German Federal Ministry of Education and Research	Cross-sectional	98	The non-westernized adults comprised the highest species richness and contained medium to high levels of species diversity compared with westernized adults and non-westernized children.	[168]
**LUNG**										
**Malawi**	None	Bronchoalveolar lavage	Lung	16S rDNA, V1-V3 region, 454 pyrosequencing	Malawi, UK, USA	Wellcome Trust, NIH	Cross-sectional	44	Healthy adults in Malawi exposed to higher levels of particulates have higher abundances of potentially pathogenic bacteria (Streptococcus, Neisseria) within their lung microbiome. Domestic biomass fuel use was associated with an uncommon environmental bacterium (Petrobacter) associated with oil-rich niches.	[169]
**South Africa**	HIV-bronchiectasis and cystic fibrosis	Sputum	Lung	16S rRNA, V1-V3 region, 454 pyrosequencing	South Africa	University of Pretoria Institutional Research –Genomics 2013	Cross-sectional	27	The microbiome in children with HIV-associated bronchiectasis seems to be less rich, diverse, and heterogeneous with predominance of Proteobacteria when compared to cystic fibrosis.	[170]
**Uganda**	HIV and pneumonia	Bronchoalveolar lavage	Lung	16S rRNA, V4 region, Illumina MiSeq	Uganda, USA	NIH	Cross-sectional	182	These data provide evidence that compositionally and structurally distinct lower airway microbiomes are associated with discrete local host immune responses, peripheral metabolic reprogramming, and different rates of mortality.	[171]
**Mouth**										
**Democratic Republic of the Congo**, **Sierra Leone**	None	Saliva	Mouth	16S rRNA, V1-V2 region, 454 pyrosequencing	China, Democratic Republic of the Congo, Germany, Kenya, Sierra Leone, USA	Max Planck Society	Cross-sectional	28	The greater similarity of the saliva microbiomes of the two Pan species to one another, and of the two human groups to one another, are in accordance with both the phylogenetic relationships of the hosts as well as with host physiology.	[172]
**Democratic Republic of the Congo**, **Sierra Leone**, **Uganda**	None	Saliva	Mouth	16S rRNA, V1-V2 region, 454 pyrosequencing	Germany, USA	Max Planck Society	Cross-sectional	72	The distinctive composition of the saliva microbiome of the Batwa may have been influenced by their recent different lifestyle and diet.	[173]
**Egypt**	Endodontic infection	Endodontic samples	Mouth	16S rRNA, V3–V4 regions, Illumina MiSeq	Egypt	Personal funding	Cross-sectional	19	This study revealed that microbiota of endodontic infection with periapical lesions had high polymicrobial communities.	[174]
**South Africa**	None	Saliva	Mouth	Shotgun metagenomic sequencing, Illumina HiSeq	China, South Africa, USA	NIH, NSF grant, the San Simeon Fund, Gladstone Institutes	Cross-sectional	15	Individuals from the Kalahari carry a higher oral pathogenic microbial load than samples surveyed in the Human Microbiome Project.	[175]
**Sudan**	Endodontic infections	Tooth and surrounding swabs	Mouth	16S rRNA, V1–V2 regions, 454 pyrosequencing	Germany, Sudan	German Research Foundation	Case-control	50	The pyrosequencing analysis revealed a distinctly higher diversity of the microbiota compared to earlier reports. The comparison of symptomatic and asymptomatic patients showed a clear association of the composition of the bacterial community with the presence and absence of symptoms in conjunction with the patients’ age.	[176]
**Breast**										
**Burundi**, Italy	None	Colostrum and mature milk	Breast	16S rRNA, V2–4–8 and V3–6, 7–9 regions, Ion Torrent PGM	Australia, Italy	NA	Cross-sectional	30	The microbiota of human milk is a dynamic and complex ecosystem with different bacterial networks among different populations containing diverse microbial hubs and central nodes, which change during the transition from colostrum to mature milk.	[177]
**Central African Republic**	None	Breast milk	Breast	16S rRNA, V1–V3 regions, Illumina MiSeq	USA	NSF CAREER Award, College of Arts and Sciences, Initiative for Global Innovation Studies, Elling Fund at Washington State University	Cross-sectional	41	While the origins of the human milk microbiome (HMM) are not fully understood, our results provide evidence regarding possible feedback loops among the infant, the mother, and the mother’s social network that might influence HMM composition.	[178]
**South Africa**	None	Breast milk	Breast	16S rRNA, V4 region, Illumina MiSeq	Australia, South Africa, Tanzania	NIH, Bill and Melinda Gates Foundation	Cross-sectional	554	We identified three major microbiome profile groups, defined by the relative abundances of *Staphylococcus* spp. and *Streptococcus* spp. We found little evidence of the association of various socio-economic or psychosocial variables with the human breast milk bacteriome, but we showed that maternal age, infant birth length, and study site were associated with composition of the HBM bacteriome.	[179]
**South Africa**, China, Finland, Spain	None	Breast milk	Breast	ITS1 of 18S rRNA and 5.8S conserved fungal region, Illumina MiSeq	China, Finland, South Africa, Spain	NA	Cross-sectional	20	Our data confirmed the presence of fungi in breast milk across continents and support the potential role of breast milk in the initial seeding of fungal species in the infant gut.	[180]
**South Africa**, China, Finland, Spain	None	Breast milk	Breast	16S rRNA, V4 region, Illumina MiSeq	China, Finland, South Africa, Spain	European Research Council (ERC) under the European Union’s Horizon 2020 research and innovation program	Cross-sectional	19	Our results reveal specific milk metabolomic profiles across geographical locations and also highlight the potential interactions between human milk’s metabolites and microbes.	[181]
**South Africa**, China, Finland, Spain	None	Mature breast milk	Breast	16S rRNA, V4 region, Illumina MiSeq	China, Finland, South Africa, Spain	NIH, H3Africa Initiative, Key Projects of Beijing Science and Technology, Natural scientific foundation of Beijing	Cross-sectional	18	The results demonstrate a significant effect of geographical variations in human milk polyamine concentrations, being correlated with human milk microbiota composition. These differences may have an impact on infant development during lactations.	[182]
**South Africa**, China, Finland, Spain	None	Breast milk	Breast	16S rRNA, V4 region, Illumina MiSeq	China, Finland, South Africa, Spain	University of Turku, NIH, Beijing Science and Technology, Natural Scientific Foundation of Beijing, European Research Council ERC	Cross-sectional	20	Results demonstrate differences in the composition of lipids and microbiota in breast milk in different geographic regions and offer a new insight into the differences in development of gut microbiota in infants in different geographic areas.	[183]
**Blood and plasma**										
**Burkina Faso**	Bacterial bloodstream infections	Blood	Blood	16S rRNA, V3–V4 regions, Illumina MiSeq	Australia, Belgium, Burkina Faso	Flemish Ministry of Sciences	Cross-sectional	75	16S metagenomics is a powerful approach for the diagnosis and understanding of bacterial bloodstream infections.	[184]
**Cameroon**	HIV/AIDS	Plasma	Blood	Illumina MiSeq	Cameroon, USA	NIH, UCSF-Abbott Viral Discovery Award	Cross-sectional	35	The extensive genome coverage obtained by NGS improved accuracy and confidence in phylogenetic classification of the HIV-1 strains present in the study population relative to conventional sub-region PCR.	[185]
**Kenya**	Unexplained febrile illness	Blood	Blood	Viral shotgun metagenomic sequencing, Illumina HiSeq	Brazil, Kenya, UK, USA	NIH, Blood Systems Research Institute (USA), IAVI, USAID, other donors at IAVI website	Cross-sectional	498	The study characterizing viral nucleic acids in the plasma of a febrile East African population has demonstrated a relatively high frequency of parvovirus B19 and dengue infections and revealed a novel human arbovirus, providing a baseline to compare with future virome studies to detect emerging viruses in this population.	[186]
**Nigeria**	Unexplained acute febrile illness	Blood	Blood	RNA seq, Illumina HiSeq for viruses	Australia, Nigeria, USA	NIH, Packard Foundation Fellowship for Science and Engineering, Broad Institute	Case-control	523	The results suggest that rhabdovirus infections could be common and may not necessarily cause overt disease. The identification of viral nucleic acid sequences in apparently healthy individuals highlights the need for a broader understanding of all viruses infecting humans as increased efforts are made to identify viruses causing human disease.	[187]
**Tanzania**	Unexplained febrile illness	Plasma	Blood	RNA sequencing, VirCapSeq-VERT, Illumina HiSeq	Switzerland, Tanzania, USA	Bill and Melinda Gates Foundation, NIH, Swiss National Science Foundation	Observational cohort	12	High-throughput sequencing (HTS) provides a comprehensive analysis of the plasma virome and is particularly well suited to situations where an infectious etiology has yet to be determined. Currently, the applicability of HTS in diagnostic medicine is limited by both cost and complexity of analysis.	[188]
**Uganda**, USA	AIDS	Plasma	Blood	Illumina MiSeq for Virus detection	Thailand, Uganda, USA	NIH, Blood Systems Research Institute (USA)	Cross-sectional	23	Several viruses were found in the plasma during this study, but it is possible that other viruses were not detected.	[189]
**BRAIN AND SPINAL CORD**										
**Ghana**	Encephalitis and meningoencephalitis	Cerebrospinal fluid	Brain and spinal cord	Viral shotgun metagenomics, Illumina MiSeq	Germany, Ghana	NA	Cross-sectional	70	This study increases the current knowledge on the genetic diversity of *Torque teno* mini viruses and strengthens that human anelloviruses can be considered biomarkers for strong perturbations of the immune system in certain pathological conditions.	[190]
**HAND**										
**Tanzania**, USA	None	Hand-wash samples	Hand	16S rDNA, V3–V5 regions, 454 pyrosequencing	USA	The Alfred P. Sloan Foundation, The Yale University Global Health Initiative, Stanford University’s School of Earth Sciences, Center for African Studies and Woods Institute for the Environment	Cross-sectional	29	The most abundant bacterial taxa on Tanzanian hands were soil-associated Rhodobacteraceae and Nocardioidaceae.	[191]
**Skin**										
**Benin**, USA, The Netherlands	Buruli ulcer	Skin biopsies	Skin	16S rRNA, V3–V4 regions, Illumina MiSeq	Belgium, Benin	The Medicor UBS Optimus Foundations and the Department of Economy, Science and Innovation of the Flemish Government	Case-control	9	The study suggests that Buruli ulcer may lead to changes in the skin bacterial community within the lesions.	[192]
**Egypt**	Atopic dermatitis	Skin swabs	Skin	16S rRNA, V1-V3 region, Illumina MiSeq	Egypt	NA	Case-control	95	Finally, AD-related differences in skin bacterial diversity appeared to be in part linked to the serum IgE level. These new observations attest to the promise of microbiome science and metagenomic analysis in AD specifically, and clinical dermatology broadly.	[193]
**Madagascar**	None	Skin swabs	Skin	16S rRNA, V3–V4 regions, Illumina MiSeq	USA	The Duke Global Health Institute and the Bass Connections Program at Duke University	Cross-sectional	20	Cattle ownership had, at best, weak effects on the human skin microbiome.	[194]
**Madagascar**	None	Skin swabs	Skin	16S rRNA, V3–V4 regions, Illumina MiSeq	USA	Duke University	Cohort	20	We found that antibacterial soap impacts the structure of microbial communities and that these changes persist for at least two weeks.	[195]
**South Africa**	None	Skin swabs	Skin	Phage sequencing, Illumina MiSeq	France, South Africa	L’Oréal Research & Innovation Grant	Cross-sectional	6	This study describes an enriched human skin metavirome that shows similar phage signatures to the only other dataset dedicated to studying human skin virus populations.	[196]
**More than one body site**										
**Cameroon**	None	Stool and saliva	Gut and mouth	16S rRNA, V4 region, Illumina MiSeq	Cameroon, France, South Africa, USA	ANR grant, a CNRS INEE grant, the Center for Microbiome Informatics and Therapeutics at MIT, Rasmussen Family Foundation to the Global Microbiome Conservancy	Case-control	147	Urbanization was associated with minor shifts in diversity of the gut and saliva microbiome, but also with changes in the gut microbiome composition that were reminiscent of those associated with industrialization.	[46]
**Ethiopia**, **Kenya**, **The Gambia**, **Ghana**, Peru, Spain, Sweden, USA	None	Breast milk and stool	Breast and gut	16S rRNA, V1–V3 regions, Illumina MiSeq	Canada, Ethiopia, The Gambia, Ghana, Kenya, Peru, Spain, Sweden, USA	National Science Foundation, the Ministry of Economy and Competitiveness (Spain), European Commission, supported in part by NIH COBRE	Cross-sectional	209	Our data provide additional evidence of within- and among-population differences in milk and infant fecal bacterial community membership and diversity and support for a relationship between the bacterial communities in milk and those of the recipient infant’s feces.	[197]
**Ghana**, **South Africa**, Jamaica, USA	Metabolic syndrome and cardiometabolic risk	Stool and saliva	Gut and mouth	16S rRNA, V4 region, Illumina MiSeq	Ghana, Jamaica, Republic of Seychelles, South Africa, Switzerland, USA	NIH	Cross-sectional	372	Our findings extend our insights into the relationship between the human microbiota and elevated CM risk at the structural and functional level, pointing to possible future therapeutic modalities for CM risk targeting the gut and oral microbiota.	[198]
**Kenya**	Respiratory syncytial virus	Nasopharyngeal and oropharyngeal or nasal swabs	Nasopharynx, oropharynx, and nose	16S rRNA, V3–V4 regions, Illumina MiSeq	Kenya, UK	Wellcome Trust	Case-control	84	Airway secretions of children infected with RSV have significantly greater antibacterial activity compared to respiratory syncytial virus RSV-negative controls. This RSV-associated, neutrophil-mediated antibacterial response in the airway appears to act as a regulatory mechanism that modulates bacterial growth in the airways of RSV-infected children.	[199]
**Malawi**	Unexplained paraplegia	Cerebrospinal fluid and Serum	Brain, spinal cord, and blood	Viral shotgun metagenomic sequencing, 454 pyrosequencing	Malawi, The Netherlands	European Commission, Virgo Consortium	Cross-sectional	12	A novel cyclovirus species was identified and subsequently found in 15% and 10% of serum and cerebrospinal fluid samples, respectively.	[200]
**Malawi**	Severe chorioamnionitis and adverse birth outcomes	Placental and fetal membrane, vaginal, and dental swabs	Placental and fetal membrane, urogenital, and mouth	16S rRNA, V5–V7 regions, Illumina MiSeq	Finland, Malawi, UK, USA	USAID, Bill and Melinda Gates Foundation, The Academy of Finland, Competitive State Research Financing of the Expert Responsibility Area of Tampere University Hospital	Cross-sectional	1391	Results provide data on the role of the vaginal microbiome as a source of placental infection as well as the possibility of therapeutic interventions against targeted organisms during pregnancy.	[201]
**Mozambique**	HIV-2	Stool and blood	Gut and blood	16S rRNA, V3–V4 regions, Illumina MiSeq, Shotgun metagenomics, Illumina HiSeq	Germany, Mozambique, Spain	Fondo de Investigaciones Sanitarias, Instituto de Salud Carlos III, European Regional Development Fund, Bill and Melinda Gates Foundation, Fundació Glòria Soler, People in Red, Fundació Catalunya La Pedrera	Prospective and controlled cohort	202	Our study shows that HIV-1 infection is followed by increased fecal Adenovirus shedding and by transient, non-HIV-specific changes in the gut bacteriome.	[202]
**Nigeria**	Lassa fever	Plasma, breast milk, or cerebrospinal fluid samples	Breast, blood, brain, and spinal cord	Shotgun metagenomics, MinION Oxford Nanopore, Illumina MiSeq	Belgium, Germany, Nigeria, Singapore, Switzerland, UK, USA	National Institute for Health Research, German Government, the European Union	Cross-sectional	120	Portable metagenomic sequencing of genetically diverse RNA viruses on the MinION, direct from patient samples without the need to export material outside of the country of origin and with no pathogen-specific enrichment, is shown to be a feasible methodology enabling a real-time characterization of potential outbreaks in the field.	[203]
**South Africa**	HIV-1	Plasma and cervicovaginal lavage	Urogenital and blood	Shotgun metagenomics, Illumina MiSeq, and Hiseq	USA	NIH, Bill and Melinda Gates Foundation, Burroughs Wellcome Fund	Prospective cohort	3	The use of metagenomic sequencing allowed us to characterize other organisms in the female genital tract, including commensal bacteria and sexually transmitted infections, highlighting the utility of the method to sequence both HIV-1 and its metagenomic environment.	[204]
**Tanzania**	None	Stool and hand swabs	Gut and skin	16S rRNA, V1–V3 and V3–V5 regions, Illumina MiSeq, Shotgun metagenomics, Illumina HiSeq	Canada, Tanzania, UK, USA	Emch Family Foundation and Forrest & Frances Lattner Foundation, C&D Research Fund, NIH, Discovery Innovation Fund Award	Longitudinal	188	This work serves as a snapshot of the state of the Hadza microbiota in the context of environment, diet, and lifestyle that can inform our understanding of the microbiota across a diverse set of populations.	[15]
**Tanzania**	None	Stool, saliva, vaginal swabs, and breast milk	Gut, mouth, urogenital, and breast	16S rRNA, V4 region, Illumina MiSeq	Canada, Tanzania, USA	Bill and Melinda Gates Foundation	Longitudinal	56	Daily micronutrient-supplemented probiotic yogurt provides a safe, affordable for pregnant women in rural Tanzania, and the resultant improvement in the gut microbial profile of infants is worthy of further study.	[22]
**Uganda**	HIV-pneumonia	Bronchoalveolar lavage and stool	Lung and gut	16S rRNA, V4 region, Illumina NextSeq, ITS2 of rRNA, Illumina MiSeq	Uganda, USA	NIH	Cohort	106	Gut microbiome is related to CD4 status and plays a key role in modulating macrophage function, critical to microbial control in HIV-infected patients with pneumonia.	[205]
**Uganda**	Pediatric febrile illness	Serum, nasopharyngeal, and stool	Nasopharynx, blood, and gut	Shotgun metagenomics, Illumina HiSeq	Uganda, USA	Doris Duke Charitable Foundation	Retrospective study	94	In this retrospective exploratory study, mNGS identified multiple potential pathogens, including 3 new viral species, associated with fever in Ugandan children.	[206]
**Uganda**	Severe acute respiratory infection	Naso- and oropharyngeal swabs	Nasopharynx and oropharynx	VirCapSeq-VERT, Illumina HiSeq	Uganda, USA	NIH, David R. Nalin, MD ‘65 Fund for International Research at Albany Medical College, Stony Wold-Herbert Fund, African Academy of Sciences, Alliance for Accelerating Excellence in Science in Africa, Wellcome Trust, UK Government	Cross-sectional	2901	Using a precision approach to public health surveillance, we detected and characterized the genomics of vaccine-preventable and zoonotic respiratory viruses associated with clusters of severe respiratory infections in Uganda.	[207]
**Zambia**	Meningitis	Cerebrospinal fluid	Brain and spinal cord	Full-length 16S rRNA, MinION Oxford Nanopore	Japan, Zambia	Japan Initiative for Global Research Network on Infectious Diseases of the Japan Agency for Medical Research and Development, Takeda Science Foundation and Japanese Ministry of Education, Culture, Sports, Science and Technology (MEXT)-Supported Program for the Strategic Research Foundation at Private Universities	Cross-sectional	11	Our results suggest that time-effective analysis could be achieved by determining the number of sequencing reads required for the rapid diagnosis of infectious bacterial species depending on the complexity of bacterial species in a sample.	[208]

### Distribution of studies across Africa

**Fig. 3:**
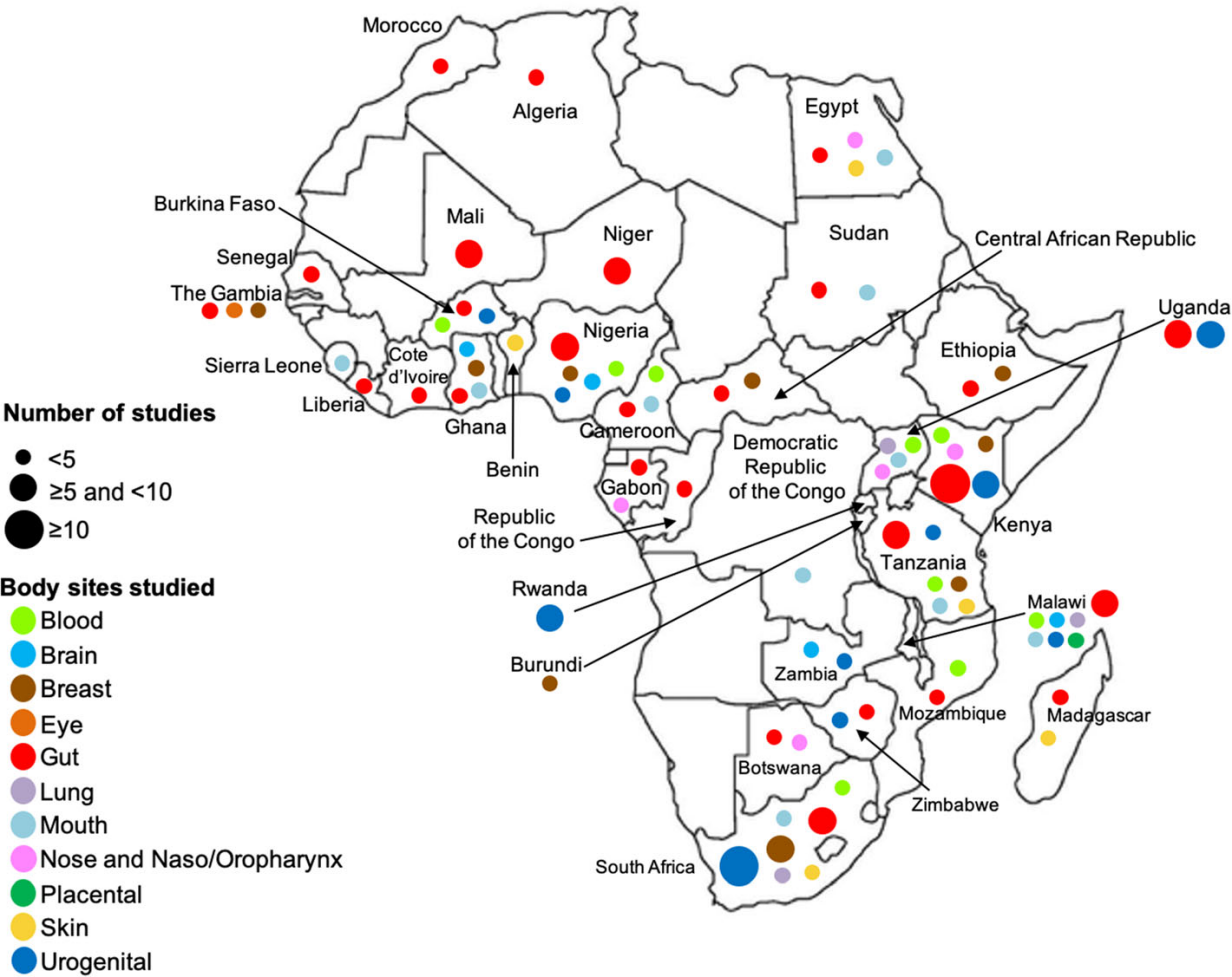
An African map showing the location, frequency, and body sites investigated in human microbiome studies

We analyzed the African countries of sample origin for all 168 eligible papers. The included studies collected samples from participants residing in 33 of the 54 countries in Africa (61%) (Fig. 3). The countries with the highest number of studies were South Africa (16.1%, 27/168), Kenya (13.7%, 23/168), and Uganda (10.7%, 18/168). Tanzania (7.1%, 12/168), Malawi (7.1%, 12/168), and Nigeria (6.5%, 11/168) also had a moderate number of studies conducted in them. The 27 remaining countries had less than ten studies each. Regionally, most of the studies were conducted in East Africa (39.9%, 67/168) followed by Southern (29.8%, 50/168), West (29.2%, 49/168), Central (7.7%, 13/168), and North Africa (6.5%, 11/168). The region with the highest coverage of countries was West Africa, where studies were conducted in 11/15 countries (73%). This was followed by Central Africa 4/7 (57%), East Africa 8/14 (57%), and North Africa 4/7 (57%) and finally Southern Africa 6/11 (55%).

### Body sites, sample types, methodology, and data archiving

**Fig. 4:**
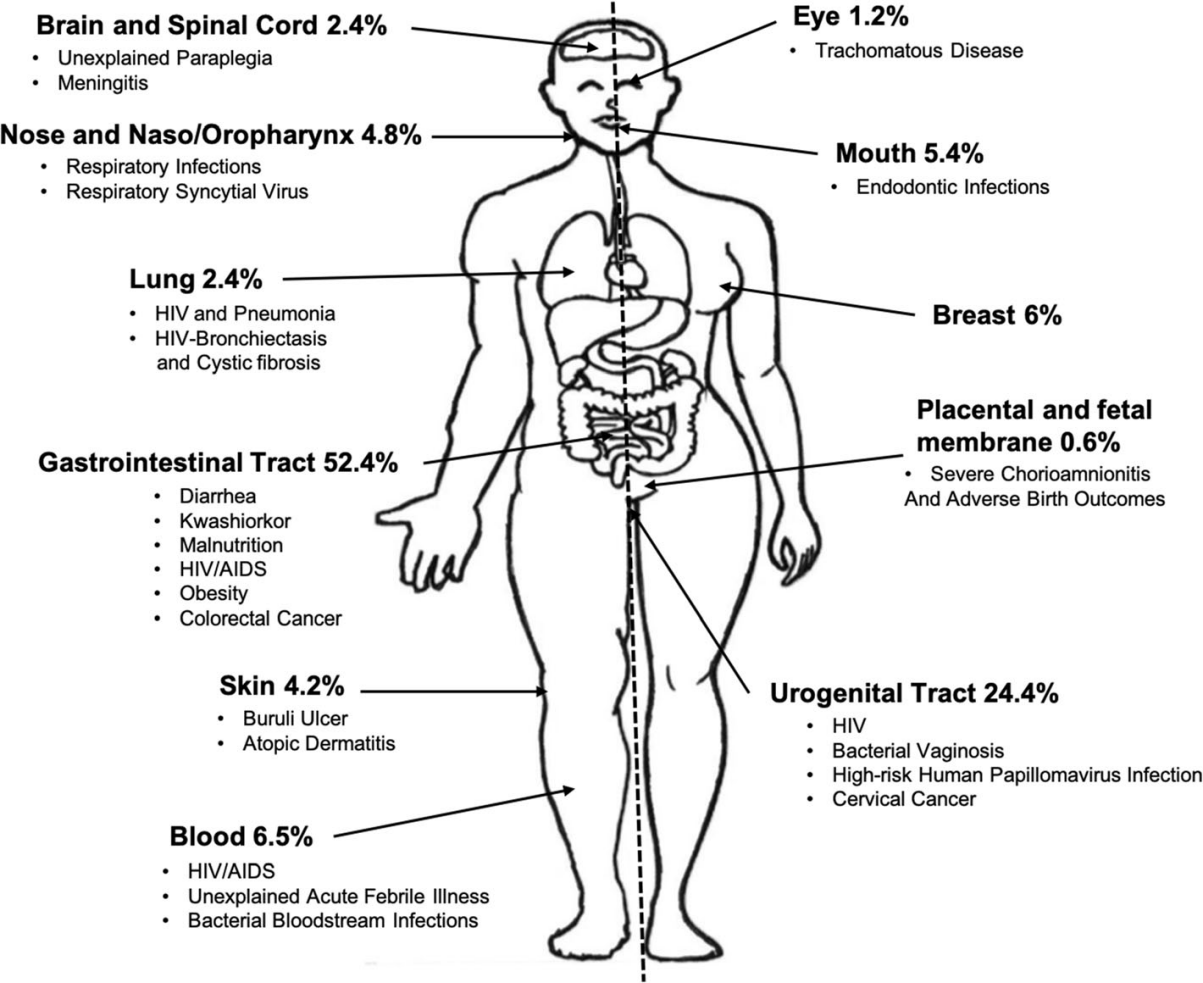
Representation of body sites included in microbiome studies conducted in Africa. The most studied diseases are listed for each body site. The total percentage exceeds 100% because eight, three, and one study characterized two, three, and four body sites respectively

The gastrointestinal tract (GIT) was the most studied body site (52.4%, 88/168, Table 1 and Table S2a), followed by the urogenital tract (24.4%, 41/168, Table 2 and Table S2b) while the eye (1.2%, 2/168, Table 3 and Table S2c) and placenta (0.6%, 1/168, Table 3 and Table S2c) were the least studied sites (Fig. 4). Similarly, the predominant sample type studied was stool (47.6%, 80/168) followed by vaginal samples (16.1%, 27/168). Placenta and fecal membrane samples were the least frequently studied (0.6%, 1/168). A total of 144 studies investigated the bacterial component, while 14 characterized the virome of the human microbiota. One study each focused on only fungi and only protozoa. While two studies investigated both bacteria and viruses, one each focused on bacteria and fungi collectively, and then bacteria and protozoa. Two studies explored viruses, bacteria, and the protozoal component of the microbiota while the remaining two studies investigated helminths in addition to the former three.

The majority of studies characterizing the bacteriome used only 16S rRNA amplicon sequencing (73.8%, 124/168) while 14/168 (8.3%) used only shotgun metagenomic sequencing. Eleven studies (6.5%) used both methods. One study targeted the *cpn*60 gene in place of the 16S rRNA gene for bacteriome characterization. For virome studies, shotgun metagenomics was used in seven studies while targeted methods including RNA sequencing, phage sequencing, and VirCapSeq-VERT were used in four studies. Targeted sequencing of the ITS1 of 18S rRNA and 5.8S conserved fungal region was used to characterize the mycobiome in one study. One study conducted full-length 16S rRNA sequencing while two utilized both 16S rRNA sequencing and ITS2. One study included both MALDI-TOF culturomics and 16S rRNA sequencing technologies. Two studies failed to specify the method used; however, they were included in the final analysis because they utilized high throughput sequencing. The platform most commonly used was Illumina MiSeq (57.1%, 96/168), followed by Roche 454 pyrosequencer (22.6%, 38/168) and Illumina HiSeq (14.3%, 24/168). Of the 168 studies, 64 (38.1%) did not indicate whether their data are publicly available (Tables S2a-S2c). However, for those that did, most (29.8%, 50/168) deposited their sequence data in the National Center for Biotechnology Institute Sequence Read Archive (NCBI-SRA). Similarly, 20/168 (11.9%) data sets were archived in The European Nucleotide Archive (ENA), 9/168 (5.4%) in GenBank, 5/168 (3.0%) in Metagenomics Rapid Annotation using Subsystems Technology (MG-RAST), 3/168 (1.8%) in Open Science Framework (OSF), and 2/168 (1.2%) in the DNA Data Bank of Japan, and 1/168 (0.6%) in NCBI Gene Expression Omnibus (GEO). Ten studies (6.0%) deposited data in more than one of the repositories mentioned above, while four studies (2.4%) indicated that they would make their data available upon request.

### Study participant information

**Fig. 5:**
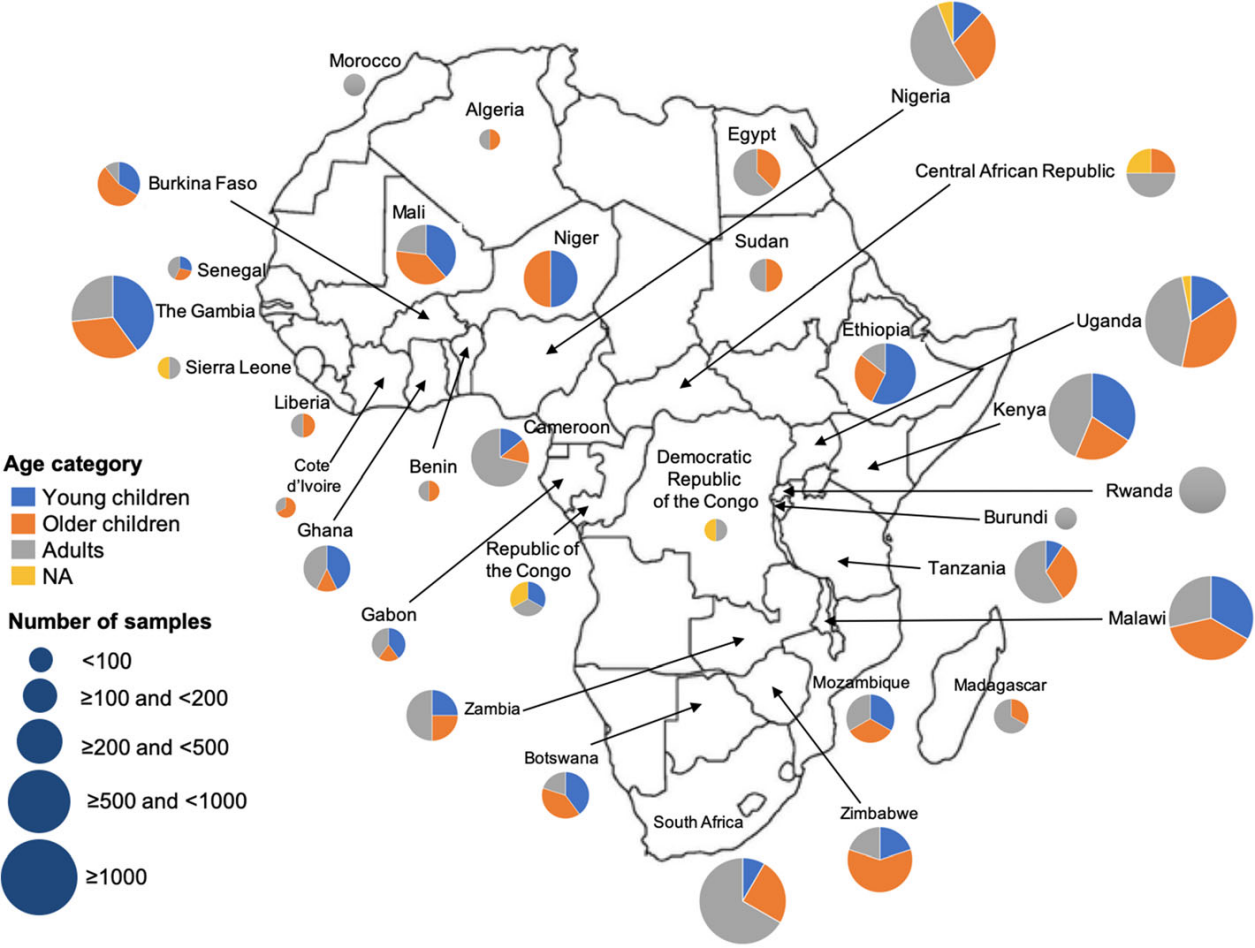
African map with pie charts showing the age categories and the number of participants included in human microbiome studies per country. The size of the divisions within the pie charts corresponds to the proportion of studies that included each age category (young children (0 to 5 years), older children (6 to 17 years), and adults (≥ 18 years)). The size of the pie chart represents the cumulative number of participants from all studies conducted in the country

**Fig. 6:**
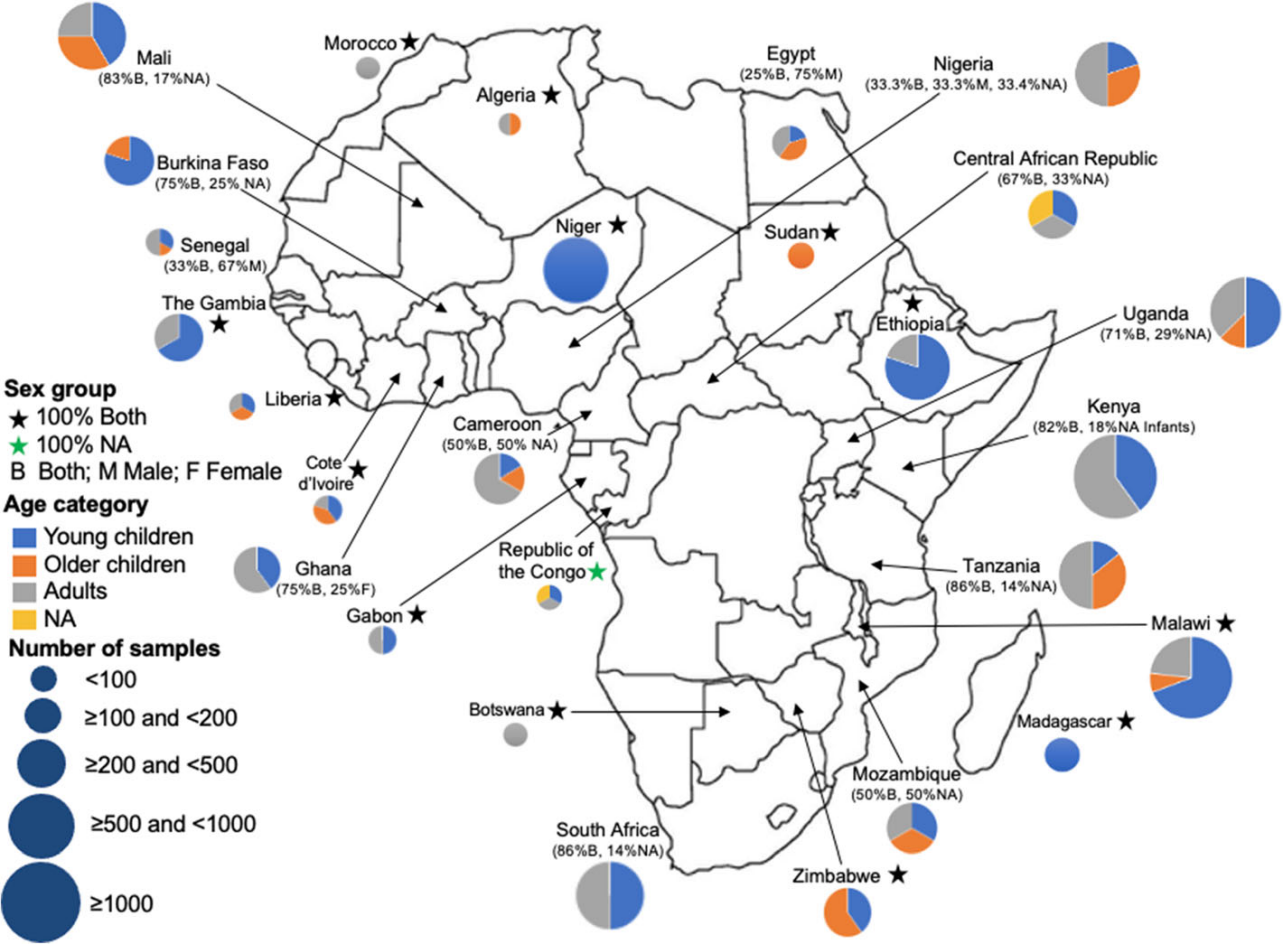
African map with pie charts showing the age categories and the number of participants included in human gut microbiome studies per country. The size of the divisions within the pie charts corresponds to the proportion of studies that included each age category (young children (0 to 5 years), older children (6 to 17 years), and adults (≥ 18 years)). The size of the pie chart represents the cumulative number of participants from all studies conducted in the country

**Fig. 7:**
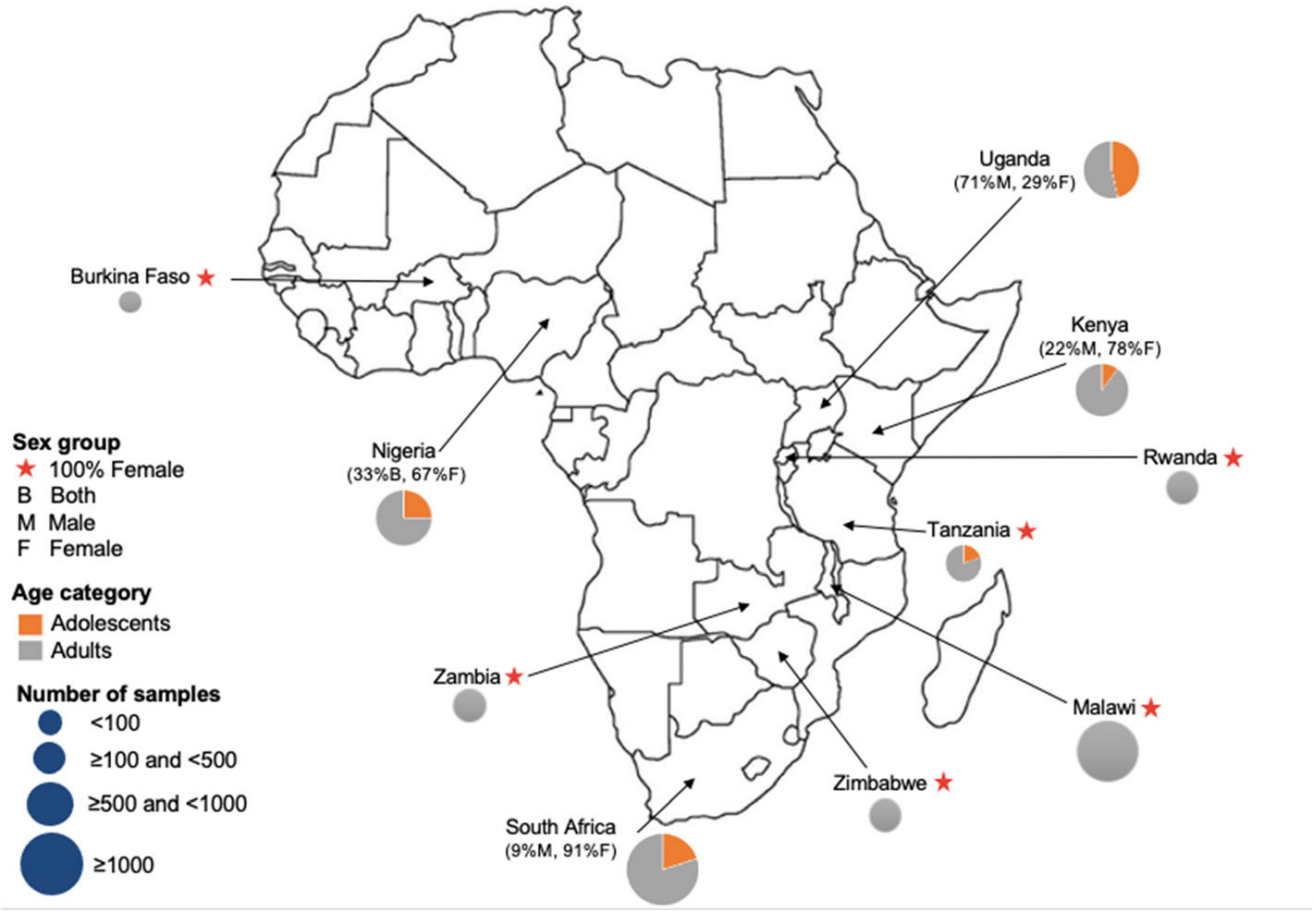
African map with pie charts showing the age categories and the number of participants included in human urogenital microbiome studies per country. The size of the divisions within the pie charts corresponds to the proportion of studies that included each age category (adolescents (13 to 17 years) and adults (≥ 18 years)). The size of the pie chart represents the cumulative number of participants from all studies conducted in the country

More studies (42.2%, 71/168) investigated adult [≥ 18 years] microbiomes than those of young children [0 to 5 years] (23.8%, 40/168). No study focused on only older children [6 to 12 years old] or adolescents [13 to 17 years] (Fig. 5). However, 31.5% (53/168) of the studies compared the microbiomes of more than one age group. While 51.8% (87/168) of the studies included both males and females, 24.4% (41/168) and 11.3% (19/168) included only females and only males, respectively. The sex of participants was not specified in 19 (11.3%) studies. The two (1.2%) remaining studies included mothers and their infants however, the sex of the infants was undefined. A total of 84.5% (142/168) of the studies did not specify the ethnicity of their participants. While 31% (52/168) of studies focused on participants in rural settings and 4.8% (8/168) investigated microbiomes of urban dwellers, five studies (3%) collected samples from residents in peri-urban communities and 54.7% (92/168) did not specify whether their participants were from rural or urban settings. Only two studies compared microbiomes of participants from rural, urban and semi-urban settings. Eight studies compared rural and urban while one study compared rural and semi-urban residents’ microbiomes. Most studies [60% (101/168)] included less than 100 participants, while 31.5% (59/168) studies enrolled 100 to 499 participants. Six studies included 500 to 999 participants. Only two studies involved 1000 or more people (Fig. 5). In total, Nigeria, the Gambia, Kenya, Malawi, South Africa, and Uganda had the microbiome of more than 1000 residents characterized. Additionally, several of the studies were derived from the same cohort of people [21] and [22–24] and [25–28] and [29, 30] and [31, 32] and [33]. Figure 6 and Figure 7 summarize the gut and urogenital studies in Africa.

### Diseases of focus of the studies

To identify the extent to which the studies focused on diseases of major public health importance in Africa, we analyzed the diseases of focus. Of the 168 eligible studies, 38.1% (64/168) did not focus on any specific disease (Fig. 4). Of the remaining 61.9% (104/168) that investigated the microbiome in the context of a specific disease, 45 studies focused on the top nine diseases responsible for the highest morbidity and mortality in Africa. They are as follows: lower respiratory infections (4), HIV/AIDS (29), diarrheal diseases (6), malaria (2), preterm birth complications (1), tuberculosis (1), and neonatal sepsis/infections (2). Other diseases that were frequently studied included malnutrition (8/104), bacterial vaginosis (5/104), obesity only (2/104), diabetes only (2/104), obesity and diabetes (1/104), and metabolic syndrome (1/104). Under neglected tropical diseases, only one study investigated Buruli ulcer, two each focused on trachoma and schistosomiasis and four on other parasitic infections (helminths and blastocystis). Fifteen studies examined non-communicable diseases (cancers, anemia, atopic dermatitis, environmental enteric dysfunction, and toxic blood metal levels).

### Intercontinental and intra-continental collaborations among study co-authors

**Fig. 8:**
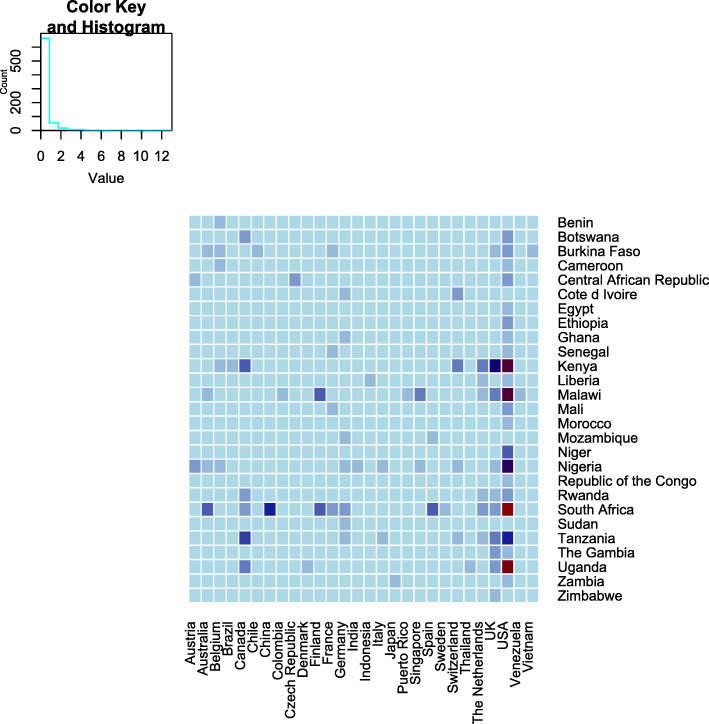
Heatmap of intercontinental collaborations between African countries and non-African countries

We analyzed the countries of institutional affiliation of all authors on each manuscript in order to understand the extent and pattern of collaborations between researchers in Africa and the rest of the world. For within-country collaborations, 17 studies had all the collaborating scientists based within the same country [Egypt (6), USA (5), South Africa (3), France (2), and Germany (1)]. Out of these, Egypt and South Africa were the only African countries where the collaborating scientists were from the same country. Furthermore, seven of the studies that involved researchers collaborating from more than one country did not include any African scientists as an author (Table 4). Asian countries whose scientists collaborated with African scientists included China, India, Bangladesh, Indonesia, Thailand, and Vietnam. Scientists from South America who collaborated with African scientists were based in Colombia, Brazil, Puerto Rico, Venezuela, and Chile. A total of 85.7% (144/168) of the studies involved intercontinental collaborations between one African country and one or more non-African countries (Fig. 8). Among these studies, the most significant collaborative efforts were between scientists in the USA and African countries, mainly South Africa (13/168), Uganda (12/168), Kenya (10/168), and Malawi (10/168). Intercontinental collaboration was also common between African scientists and researchers based in the UK, Canada, and the Netherlands.

**Table 4:** Different types of collaborations in the African Microbiome studies (intra-continental, collaborations from the same country, and between non-African countries)

Intra-continental Collaborations in Africa		Collaborations from the same Country		Collaborations between non-African Countries	
Reference	Collaborators	Reference	Collaborators	Reference	Collaborators
Li, 2013 [172]	**Democratic Republic of the Congo, Sierra Leone, Kenya**, Germany, China, USA	Aly, 2016 [75] Tawfik, 2018 [174] Ahmed, 2019 [167] Ramadan, 2019 [193] Sahly, 2019 [76] Salah, 2019 [77]	**Egypt**	De Filippo, 2010 [63]	Italy, Belgium
Jaeggi, 2014 [85]	**South Africa, Kenya**, The Netherlands, Switzerland	Masekela, 2018 [170] Onywera, 2019 [151] Onywera, 2019 [150]	**South Africa**	Nasidze, 2011 [173]	USA, Germany
Pop, 2014 [91]	**The Gambia, Mali, Kenya**, Bangladesh, UK, USA	Colson, 2013 [118] Lokmer, 2019 [65]	France	Mehta, 2012 [136]	USA, Canada
Brazier, 2017 [72]	**Gabon, Republic of the Congo**, France	Camarinha-Silva, 2014 [168]	Germany	Morton, 2015 [66]	USA, France
Tidjani Alou, 2017 [111]	**Niger, Mali, Senegal**, UK, France	Hospodsky, 2014 [191] Manus, 2017 [194] Yu, 2018 [195] Meehan, 2018 [178] Piantadosi, 2019 [204]	USA	Rampelli, 2015 [32]	USA, Italy, Germany
Cinek, 2018 [115]	**Nigeria, Sudan**, Azerbaijan, Czech Republic, Jordan			Davis, 2017 [82]	USA, UK
Liu, 2018 [105]	**Mali, Mozambique**, India, Pakistan, USA			Drago, 2017 [177]	Italy, Australia
Oldenburg, 2018 [64]	**Burkina Faso, South Africa**, Germany, USA				
Popovic, 2018 [99]	**Kenya, Malawi**, Canada, The Netherlands, USA				
Vonaesch, 2018 [68]	**Central African Republic, Madagascar**, Canada, France,				
Atukunda, 2019 [130]	**South Africa, Uganda**, The Netherlands, Norway				
Bourke, 2019	**Uganda, Zimbabwe**, Canada, UK				
Fei, 2019 [198]	**Ghana, South Africa**, Jamaica, Republic of Seychelles, Switzerland, USA				
Hansen, 2019 [48]	**Botswana, Tanzania**, Finland, UK, USA				
Lackey, 2019 [197]	**Ethiopia, The Gambia, Ghana, Kenya**, Canada, Peru, Spain, Sweden, USA				
Lane, 2019 [81]	**Ethiopia, The Gambia, Ghana, Kenya**, Canada, Peru, Spain, Sweden, UK, USA				
Ojo-Okunola, 2019 [179]	**South Africa, Tanzania**, Australia				
Oldenburg, 2019 [62]	**Burkina Faso, Niger, South Africa**, Germany, USA				
Seck, 2019 [60]	**Mali, Senegal**, France, French Polynesia, Saudi Arabia				
Flygel, 2019 [132]	**South Africa, Zimbabwe**, Australia, Norway, UK				
Gudza-Mugabe, 2020 [161]	**South Africa, Zimbabwe**, USA				
Lokmer, 2020 [46]	**Cameroon, South Africa**, France, USA

### Leadership in microbiome studies

**Fig. 9:**
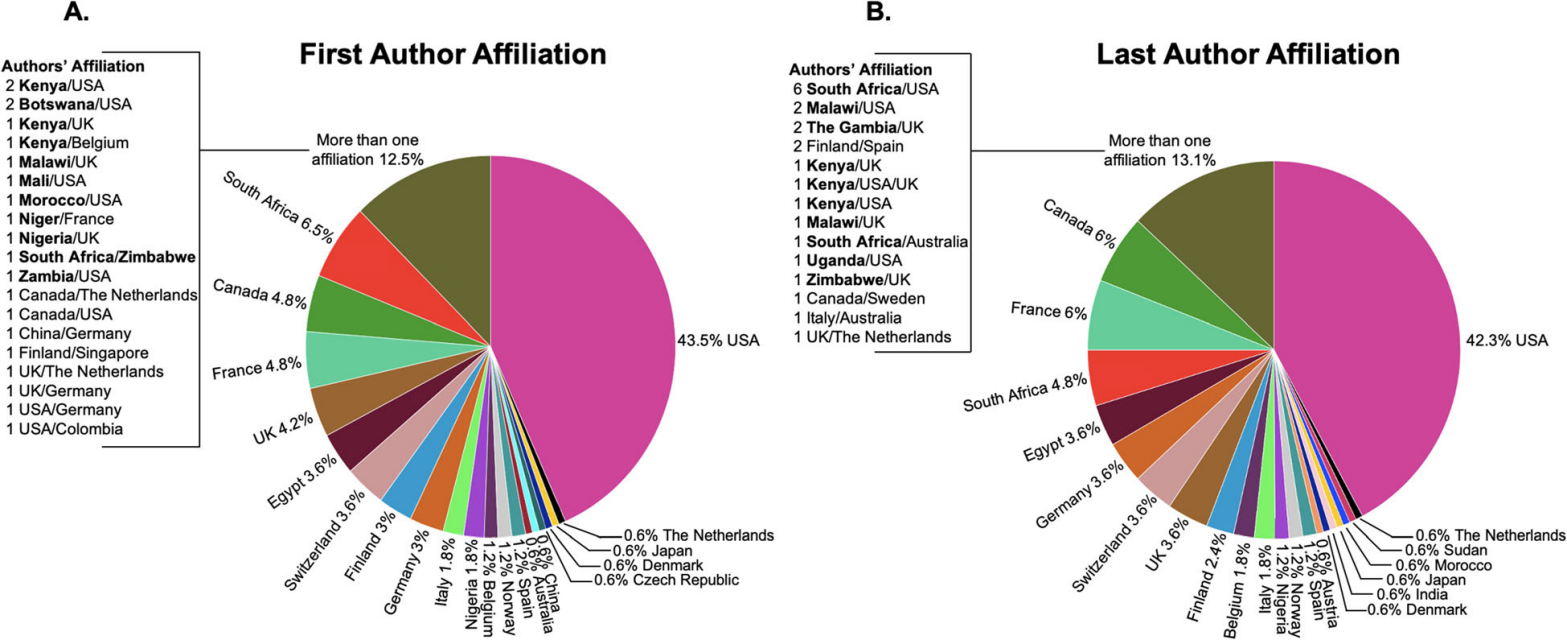
Pie charts showing the percentage of affiliations per country for the first author (**A**) and for the last author (**B**)

To determine the extent to which these studies were led by African scientists, we analyzed the countries of institutional affiliations of the first (Fig. 9A), and the senior (last) authors of the studies (Fig. 9B) as proxies. Among first authors with a single country of institutional affiliation, 43.5% (73/168) were from the USA, 6.5% (11/168) from South Africa, 4.8% (8/168) from Canada, and France and 3% (5/168) from Germany. A total of 12.5% (21/168) were affiliated with institutions in more than one country. Out of these 21 studies, the first authors of 13/21 were affiliated to both an African institution and a non-African institution while 8/21 were affiliated to two institutions from different non-African countries. Only one study had the first author affiliated to institutions in two African countries (South Africa and Zimbabwe).

South Africa 6.5% (11/168), Egypt 3.6% (6/168), and Nigeria 1.8% (3/168) were the only African countries that had a scientist with a single African country of institutional affiliation as the first author.

In contrast, other first authors with affiliations to institutions in Africa (Nigeria, Kenya, Mali, Botswana, Malawi, Morocco, Zambia, and Niger) also concurrently held affiliations to institutions in non-African countries mainly the USA and the UK. Regionally, the majority of these authors were from Southern and East Africa (5/13 from Southern Africa and 4/13 from East Africa compared to 3/13 from West Africa, one from North, and none from Central Africa).

The affiliations of the last authors followed a similar pattern (Fig. 9B); 42.3% (71/168) were from the USA, followed by Canada 6% (10/168), France 6% (10/168), South Africa 4.8% (8/168) and Germany, Switzerland and the UK (3.6% (6/168) each). Thirteen percent (22/168) were affiliated to institutions in more than one country, mainly the USA, the UK, and Australia. Similar to the observation made with the first authors, South Africa (8/168), Nigeria (2/168), and Egypt (6/168), and, in this case, Morocco (1/168) were the only African countries that had a scientist with a single African country of institutional affiliation as the last author.

African researchers from South Africa, Zimbabwe, Kenya, Malawi, Uganda, and The Gambia were also simultaneously affiliated to institutions in other countries outside the African continent, mainly the USA and the UK. Regionally, the majority of these authors were from Southern and East Africa (11/17 from Southern Africa and 4/17 from East Africa compared to 2/17 from West Africa and none from North and Central Africa). Using the first and last authors as proxies for the leadership of studies, we found that 79.8% of all the studies had first and/or last authors affiliated to institutions outside Africa.

### Funding

We analyzed the agencies that directly supported the studies by awarding research grants. We found that funding from USA sources predominated (Fig. 2), with more than 70% of the studies partially or fully funded by American governmental institutions, foundations, and agencies. These included the National Institute of Health (NIH) 76/168 (45.2%), Bill and Melinda Gates Foundation 30/168 (17.8%), the United States National Science Foundation 8/168 (4.8%), and Blood Systems Research Institutes 5/168 (3.0%). This was followed by the European Union 20/168 (11.9%), through the European and Developing Countries Clinical Trials Partnership (EDCTP) (4/20), European Research Council (5/20), European Union Regional development fund (2/20), European Union’s Seventh Framework program (3/20) and other European Union agencies (6/20). Other funding sources included the Wellcome Trust (UK) 14/168 (8.3%), and the Canadian Institute of Health Research (CIHR) 8/168 (4.8%). It is noteworthy that South Africa is the only African country to have funded a published microbiome study on the continent.

## Discussion

We conducted a systematic survey of studies that utilized NGS to characterize the human microbiome of residents of Africa. Our results revealed that up to 1^st^ April 2020, 168 published studies utilized NGS to characterize the human microbiome of African participants. Of the 61.9% (104) of studies that examined the microbiome in the context of disease, less than half (43.3%, 45/104) focused on diseases that are responsible for the highest morbidity and mortality in Africa with HIV/AIDS accounting for 29/45 studies alone. With regard to collaboration, partnerships between the USA and African scientists were most common. However, the leadership of these studies (first and last authorship) was mainly assumed by the American scientists.

### African human microbiome publications

With the advances in NGS extending over a decade, it is interesting that half of all the studies were only published within the past 3 years (Fig. 2). It is, however, disappointing that only 168 studies investigated the African human microbiome using this technology. Considering that Africa is made up of 54 independent countries with extremely diverse genetic backgrounds and cultures and is also the second most populous continent with a population of 1.3 billion (2018 estimates [34]), Africa is under-represented in the global microbiome literature. More than half of the studies involved less than 100 participants further suggestive of reduced coverage. Additionally, several of the publications were derived from the same cohort of people [21] and [22–24] and [25–28] and [29, 30] and [31, 32] and [33], which further reduces the diversity and coverage of African people included in microbiome studies. Since the literature search extended only to April 2020, the numbers reflected for this year are lower.

Most studies (73.2%) involved a single sampling time point. Cross-sectional designs are appropriate for studies that aim to describe the microbiome signatures associated with a particular outcome of interest [35]. However, owing to high within-subject and between-subject variability and the influence of environmental factors, longitudinal study designs with multiple temporally-separated sampling points are recommended for more robust and reproducible results [35]. Cross-sectional designs were common probably because of the following factors: budgetary constraints, invasiveness of sampling procedure, participant compliance to study protocol, and availability of samples in the case of retrospective studies [35]. With regard to budgetary constraints, multiplexing techniques [36, 37] allow multiple samples from the same or even different origins to be processed and sequenced together. This technique substantially reduces sequencing costs.

Most (83.3%) of the studies were either published in open access journals or as open access articles in subscription-based journals or freely accessible through PubMed Central. This may be due to the open-access revolution that has gained ground in the scientific world [38]. Furthermore, the open access publishing policies adopted by the top funders (NIH, Bill and Melinda Gates Foundation, and Wellcome Trust) of the studies may also explain this observation [39]. Open-access publishing of studies conducted in Africa is crucial because the majority of libraries in African universities struggle to afford expensive subscriptions to prominent publishing companies. Although journal access initiatives such as WHO Health InterNetwork Access to Research Initiative (HINARI) [40, 41] allow access to some of these subscription-based journals, some vital research articles remain behind a paywall. Open access publishing will, therefore, improve access to studies conducted on the continent to researchers, students, and the general public. Access to research already conducted in Africa will inform, equip and encourage African scientists to engage in microbiome research. It will also encourage intra-continental collaboration by increasing the visibility of African researchers who already have the capacity to undertake microbiome research.

Similarly, 61.9% of the studies mentioned storing their sequence data in publicly available repositories mainly NCBI-SRA. The increase in data archiving for public access is fuelled in part by funder [39] and journal requirements [42]. This will allow the secondary use of the data by other researchers, particularly those in Africa who may not have the funding, capacity, and facilities to generate such data. The preference for NCBI may be influenced by the fact that most of the studies were led by America scientists who may be more familiar with NCBI-SRA than the other repositories.

The countries where most studies were conducted were in East and Southern Africa. This may be influenced by the fact that most of the first and last authors who had multiple affiliations (from both African and non-African institutions) were from East and Southern Africa. Therefore, these scientists have more opportunities through their North American/European affiliations to foster collaborations outside Africa and also secure funding for microbiome studies in these specific regions of the continent. Another reason for the over-representation of Eastern and Southern Africans in the microbiome studies may be the higher prevalence of HIV in these parts of Africa (20 million in Eastern and Southern Africa compared to 6 million in West, Central, and North Africa collectively in 2018 [43]. As a high proportion of studies focused on HIV/AIDS (29/168 compared to less than 10 for any other disease), it follows that more of such studies will be situated in these two regions to permit the recruitment of required large numbers. However, Africans have widely different genetic and cultural backgrounds [16] and this diversity may affect their microbiomes [1, 35, 44]. This variability argues for broader coverage of residents of Africa from all regions in microbiome studies.

Most of the studies reported very little metadata related to participants. For instance, 54.7% of the studies did not specify whether participants are from rural or urban areas. Other studies mentioned the hospitals where the patients were recruited without specifying any further details about the location of residence of the participants themselves. This specification is important because Africans in cities are increasingly adopting western diets and lifestyles compared to those in rural areas [12, 45]. This change in lifestyle can confound microbiome associations found in studies and must, therefore, be accounted for. Indeed, Lokmer et al. found that Cameroonians along an urbanization gradient differed by diet, habitat, and socio-cultural conditions, and this affected their gut and salivary microbiomes [46]. This difference further underscores the importance of collecting as much metadata as possible for microbiome studies.

Ethnicity information was not collected in 84.5% of the studies. Ethnicity may directly impact the microbiome, but more importantly, it is frequently strongly associated with a specific culture, lifestyle, and diet, which in turn affect the microbiome [12, 47, 48]. Failure to collect this information may be because ethnicity is not always easy to define. Also, in studies in localized geographic areas, ethnicity may be relatively homogenous and therefore not the focus of the research. Furthermore, ethnicity may be confounded by the increasing frequency of inter-marriage. Additional metadata that would add value to studies include disease status, medication exposure, family history, socio-economic status, and lifestyle (diet, smoking, alcohol consumption, physical activity) [49].

Most of the studies (124/168) utilized 16S rRNA metagenomic sequencing to profile the bacterial component of the microbiome. This limits the number of studies that have looked at the fungi, viral, and eukaryotic components of the African microbiome [50]. These other components are also important in health and disease [51, 52] and therefore warrant attention. The extensive use of 16S rRNA metagenomic sequencing limits the resolution of microbial profiles to genus level [50]. It also fails to provide the genomic as well as functional contexts of the bacteria identified [50]. Decreasing the cost of shotgun metagenomic sequencing and simplifying bioinformatic analysis techniques will tip the scale toward this superior methodology.

### African human microbiome studies focusing on diseases of significant public health concern

Apart from HIV/AIDS, which was the focus of 29 studies, few studies focused on diseases among the top 10 diseases of public health importance in Africa. Human microbiome studies focusing on diseases including malaria, diarrheal diseases, pneumonia, tuberculosis were limited. This may be due to the perceived relative low contribution of the microbiome to each of these diseases. However, the role of the microbiome in these conditions cannot be completely ruled out as limited research has been conducted in these areas. Metabolic diseases including obesity and diabetes that are mediated by the microbiome were also sparsely studied. These conditions are also highly prevalent in Africa and warrant microbiome-based investigation [17, 53]. While 38.1% of studies did not characterize the microbiome in the context of any particular disease, research on healthy individuals are important to establish what the “normal” or “healthy” microbiome is for comparative purposes. Additionally, although certain conditions such as bacterial vaginosis are not part of the top 10 diseases of public health importance, they are still relevant health issues in Africa, particularly for reproductive health outcomes which are a focus of the United Nation’s Sustainable Development goals.

### The extent and pattern of collaboration with researchers in Africa and the rest of the world

African scientists collaborated most commonly with American scientists on microbiome research projects with the latter often assuming leadership. Reasons for this observation are not known but could be speculated. One factor may be that the American partners were the principal investigators of the grants funding the studies. They may also have conducted the laboratory investigations, data analysis, and drafting of the manuscripts. The African collaborator’s primary role may have only been recruitment and sample collection [54]. The practice of scientists from the global north using African scientists as conduits to obtain samples, then shipping them away without building the capacity of their African partners or directly benefiting the continent is commonly known as “helicopter research”. To address this phenomenon, the H3Africa consortium ethics working group developed a guideline in 2018 on the ethical handling of genomic samples from Africa [55]. It calls for investigators from the global north to build the capacity of their African collaborators to equip them to work independently post projects [55]. The guideline also invites western researchers to allow for substantial intellectual contribution from African scientists on studies that draw on samples recovered from the continent [55]. For this guideline to effectively combat “helicopter research,” funding agencies could specify local capacity building as a condition for awarding grants to western scientists who partner with African scientists. Institutional review boards in Africa could consider making capacity building a requirement in studies that involve international collaboration.

Additionally, African governments must recognize the importance of research and invest in microbiome studies. Apart from South Africa, through the Department of Science and Technology, no other African country directly funded any of the microbiome research projects identified here. This factor may also contribute to African scientists’ inability to initiate and therefore lead microbiome studies. Intra-continental collaboration within Africa was almost non-existent, possibly hampered by lack of funding and language barrier. Similar findings were made by Boshoff*,* who investigated intra-regional research collaboration among countries within the Southern African Development Community (SADC) [56]. This author found only 3% and 5% of intra-regional and continental collaboration respectively in contrast to 47% inter-continental collaboration with high-income countries [56]. Onyancha et al. also observed a similar pattern for research collaborations in sub-Saharan Africa [57] where intra-continental collaboration was minimal compared to inter-continental north-south partnerships. To encourage intra-continental collaborations, Onyancha recommended regional conferences as well as student and staff exchanges [57]. However, these exchanges will have a limited impact if researchers cannot access funding to conduct projects. Lack of pathways to independent funding necessitates outside collaboration and is therefore likely to be a key limitation for African leadership on articles and grants. Access to independent funding streams is the most important factor that should be tackled to address low African leadership of microbiome studies. High reagent costs associated with microbiome studies in Africa also frequently result in the shipping of samples out of the region. Microbiome research in Africa would be greatly improved by efforts to reduce the cost per sample for assays such as 16S amplicon sequencing.

International collaborations with non-African partners followed colonial ties [54] as well as commonality of a national language, with African scientists from Francophone countries collaborating with French scientists, while English-speaking western countries partnered with Anglophone African researchers. An African scientist who collaborates with a western scientist increases his/her chances of securing funds for research, and this may explain the preference for international collaboration. Indeed, several funding agencies specifically make international collaboration, usually with a partner from the funder’s own country, a requirement for funding. This requirement further discourages intra-continental collaboration.

Computational resources to handle bioinformatics analysis are also scarce on the continent, making inter-continental partnerships important. However, the H3Africa Consortium [58] through its subsidiary, the H3ABioNet [59] has launched many initiatives to build capacities for African scientists to lead and conduct microbiome research in Africa. Additional efforts [59], including workshops by other agencies, are being made to further build bioinformatics capacity in Africa [11].

## Conclusion

Residents in Africa are under-represented in human microbiome studies. There is a need to build capacity for microbiome research in Africa, increase collaboration among scientists within Africa, and ensure equitable partnerships with international collaborators. African governments and research funding agencies should identify microbiome research as a priority area for investigation and funding.

## Limitations

Certain studies utilized the same cohort resulting in multiple counting of the same individuals. Funding information was sometimes difficult to extract as some authors did not clearly distinguish personal funding from project funds. Some African researchers may receive internal funding from within their research institutions, which may not be captured in our review. African scientists may travel abroad for educational purposes, and during this period may be affiliated with non-African institutions Although they may still return to Africa in leadership positions, this could not be assessed in this review. The number of studies that focused on priority health care areas of Africa may be underestimated due to the exclusion of publications that did not employ NGS technology.

## Supplementary Information

**Additional file 1:** **Table S1.** Details of the search terms used in the respective databases. **Table S2a.** Additional summary of African Gut Microbiome studies. **Table S2b.** Additional summary of African Urogenital Microbiome studies. **Table S2c.** Additional summary of African Microbiome studies of other body sites.

## Data Availability

All data generated or analyzed during this study are included in this published article [and its supplementary information files].

## Abbreviations

AD: Atopic dermatitis
AIDS: Acquired immune deficiency syndrome
BV: Bacterial vaginosis
BVAB: Bacterial vaginosis-associated bacteria
CAPRISA: Centre for the AIDS Program of Research in South Africa
CDC: Centers for Disease Control and Prevention
CIHR: Canadian Institute of Health Research
CINAHL: Current Nursing and Allied Health Literature
CIPHER: Collaborative Initiative for Pediatric HIV Education and Research
Cpn60 UT: Chaperonin-60 Universal Target
EDCTP: European and Developing Countries Clinical Trials Partnership
EED: Environmental enteric dysfunction
ENA: European Nucleotide Archive
ETH Global: Swiss Federal Institute of Technology
EU: European Union
GEMS: Global Enterics Multicenter Study
GOS: Galacto-oligosaccharides
*H. pylori*: *Helicobacter pylori*
HCV: Hepatitis C virus
HIV: Human immunodeficiency virus infection
HMOs: Human milk oligosaccharides
HMP: Human Microbiome Project
hrHPV: High-risk human papillomavirus infection
IAVI: International AIDS Vaccine Initiative
Ion Torrent PGM: Ion Torrent Personal Genome Machine
MALDI-TOF: Matrix-assisted laser desorption/ionization-time-of-flight
MG-RAST: Metagenomic rapid annotations using subsystems technology
MNP: Micronutrient powder
MTCT: Mother-to-child transmission
NA: Not available
NCBI-SRA: National Center for Biotechnology Institute Sequence Read Archive
NGS: Next-Generation Sequencing
NIH: National Institute of Health
NSF: National Science Foundation
OSF: Open Science Framework
*P. falciparum*: *Plasmodium falciparum*
PCR: Polymerase chain reaction
pH: Potential of hydrogen
PHiD-CV: Pneumococcal nontypeable *Haemophilus influenzae* protein conjugate vaccine
PNAS: Proceedings of the National Academy of Sciences
PrEP: Partners pre-exposure prophylaxis study
PRISMA: Preferred Reporting Items for Systematic Reviews and Meta-analyses
rDNA: Ribosomal deoxyribonucleic acid
rRNA: Ribosomal ribonucleic acid
SAM: Severe acute malnutrition
STI: Sexually transmitted infections
UK: United Kingdom
USA: United States of America
USAID: United States Agency for International Development
WHO: World Health Organization
