# Correction to: Human microbiota research in Africa: a systematic review reveals gaps and priorities for future research

**DOI:** 10.1186/s40168-022-01226-x

**Published:** 2022-01-19

**Authors:** Imane Allali, Regina E. Abotsi, Lemese Ah. Tow, Lehana Thabane, Heather J. Zar, Nicola M. Mulder, Mark P. Nicol

**Affiliations:** 1 Computational Biology Division, Department of Integrative Biomedical Sciences, University of Cape Town, Cape Town, South Africa; 2 Laboratory of Human Pathologies Biology, Department of Biology, Faculty of Sciences, and Genomic Centre of Human Pathologies, Faculty of Medicine and Pharmacy, Mohammed V University in Rabat, Rabat, Morocco; 3 Institute of Infectious Disease and Molecular Medicine, Faculty of Health Sciences, University of Cape Town, Cape Town, South Africa; 4 Department of Molecular and Cell Biology, Faculty of Science, University of Cape Town, Cape Town, South Africa; 5 Department of Pharmaceutical Microbiology, School of Pharmacy, University of Health and Allied Sciences, Ho, Ghana; 6 Division of Medical Microbiology, Department of Pathology, Faculty of Health Sciences, University of Cape Town, Cape Town, South Africa; 7 Department of Health Research Methods, Evidence and Impact, McMaster University, Hamilton, Ontario Canada; 8 Biostatistics Unit, Father Sean O’Sullivan Research Centre, St Joseph’s Healthcare, Hamilton, Ontario Canada; 9 Departments of Paediatrics and Anaesthesia, McMaster University, Hamilton, Ontario Canada; 10 Centre for Evaluation of Medicine, St Joseph’s Healthcare, Hamilton, Ontario Canada; 11 Population Health Research Institute, Hamilton Health Sciences, Hamilton, Ontario Canada; 12 Centre for Evidence-based Health Care, Faculty of Health Sciences, Stellenbosch University, Tygerberg, South Africa; 13 Department of Medicine, Faculty of Health Sciences, University of Cape Town, Cape Town, South Africa; 14 Department of Paediatrics and Child Health, Red Cross War Memorial Children’s Hospital, Cape Town, South Africa; 15 MRC Unit on Child & Adolescent Health, University of Cape Town, Cape Town, South Africa; 16 School of Biomedical Sciences, University of Western Australia, M504, Perth, WA 6009 Australia

**Correction to: Microbiome 9, 241 (2021)**

**https://doi.org/10.1186/s40168-021-01195-7**

Supplementary Data was missing from this article and has now been uploaded.

The original article has been updated.

## Supplementary Information

**Additional file 1: Supplementary Table S1.** Details of the search terms used in the respective databases. **Supplementary Table S2a.** Additional summary of African Gut Microbiome studies. **Supplementary Table S2b.** Additional summary of African Urogenital Microbiome studies. **Supplementary Table S2c.** Additional summary of African Microbiome studies (Other body sites).
