# Supplementary material for: Human microbiota research in Africa: a systematic review reveals gaps and priorities for future research
Source: Microbiome. 2021 Dec 15;9:241. doi: 10.1186/s40168-021-01195-7 (PMC8672519; doi:10.1186/s40168-021-01195-7)
Supplement: Supplementary file 2 — Additional file 1: Table S1. Details of the search terms used in the respective databases. Table S2a. Additional summary of African Gut Microbiome studies. Table S2b. Additional summary of African Urogenital Microbiome studies. Table S2c. Additional summary of African Microbiome studies of other body sites. [file 40168_2021_1195_MOESM2_ESM.docx]

Supplementary Tables

**Supplementary Table S1.** Details of the search terms used in the respective databases.

| **Database** | **Search period** | **Search strategy** |
| --- | --- | --- |
| PubMed  ISI Web of Science  EBSCOhost:  - Academic Search  Premier  - Africa-Wide  Information  - CINAHL | Up to 28 February 2018 | (microflora OR microbiota OR microbiome OR microbiomics OR metagenome OR metagenomics OR resistome OR phageome OR viromics OR virome OR mycobiome OR metabolomics OR metabolome OR metaproteomics OR metatranscriptomics)  **AND**  (Algeria OR Angola OR Benin OR Botswana OR Burkina Faso OR "Burkina Faso” OR Upper Volta OR "Upper Volta" OR Burundi OR Cameroon OR Cape Verde OR "Cape Verde" OR Central African Republic OR Chad OR Comoros OR "Iles Comores" OR Iles Comores OR Comoro Islands OR "Comoro Islands" OR Congo OR Democratic Republic Congo OR "Democratic Republic of the Congo" OR Zaire OR Djibouti OR Egypt OR Equatorial Guinea OR "Equatorial Guinea" OR Eritrea OR Ethiopia OR Gabon OR Gambia OR Ghana OR Gold Coast OR "Gold Coast" OR Guinea OR Guinea Bissau OR "Guinea Bissau" OR Ivory Coast OR "Ivory Coast" OR Cote d'Ivoire OR "Cote d'Ivoire" OR Kenya OR Lesotho OR Liberia OR Libya OR Libia OR Jamahiriya OR Jamahiryia OR Madagascar OR Malawi OR Mali OR Mauritania OR Mauritius OR Ile Maurice OR "Ile Maurice" OR Morocco OR Mozambique OR Moçambique OR Namibia OR Niger OR Nigeria OR Rwanda OR Sao Tome OR "Sao Tome" OR Senegal OR Seychelles OR Sierra Leone OR "Sierra Leone" OR Somalia OR South Africa OR "South Africa" OR Sudan OR South Sudan OR "South Sudan" OR Swaziland OR Tanzania OR Tanganyika OR Zanzibar OR Togo OR Tunisia OR Uganda OR Zambia OR Zimbabwe OR Northern Rhodesia OR "Northern Rhodesia" OR Southern Rhodesia OR "Southern Rhodesia" OR Africa OR Africa* OR Southern Africa OR West Africa OR Western Africa OR Eastern Africa OR East Africa OR North Africa OR Northern Africa OR Central Africa OR Sub Saharan Africa OR Subsaharan Africa OR Sub-Saharan Africa)  **NOT**  (Guinea pig* OR "Guinea pig*" OR Aspergillus niger OR "Aspergillus niger")  **AND**  (humans OR human) |
| Scopus | Up to 28 February 2018 | (microflora OR microbiota OR microbiome OR microbiomics OR metagenome OR metagenomics OR resistome OR phageome OR viromics OR virome OR mycobiome OR metabolomics OR metabolome OR metaproteomics OR metatranscriptomics)  **AND**  ( LIMIT-TO ( AFFILCOUNTRY , "Algeria " ) OR LIMIT-TO ( AFFILCOUNTRY , " Angola " ) OR LIMIT-TO ( AFFILCOUNTRY , " Benin " ) OR LIMIT-TO ( AFFILCOUNTRY , " Botswana " ) OR LIMIT-TO ( AFFILCOUNTRY , " Burkina Faso " ) OR LIMIT-TO ( AFFILCOUNTRY , " Upper Volta " ) OR LIMIT-TO ( AFFILCOUNTRY , " Burundi " ) OR LIMIT-TO ( AFFILCOUNTRY , " Cameroon " ) OR LIMIT-TO ( AFFILCOUNTRY , " Cape Verde " ) OR LIMIT-TO ( AFFILCOUNTRY , " Central African Republic " ) OR LIMIT-TO ( AFFILCOUNTRY , " Chad " ) OR LIMIT-TO ( AFFILCOUNTRY , " Comoros " ) OR LIMIT-TO ( AFFILCOUNTRY , " Iles Comores " ) OR LIMIT-TO ( AFFILCOUNTRY , " Comoro Islands " ) OR LIMIT-TO ( AFFILCOUNTRY , " Congo " ) OR LIMIT-TO ( AFFILCOUNTRY , " Democratic Republic Congo " ) OR LIMIT-TO ( AFFILCOUNTRY , " Democratic Republic of the Congo " ) OR LIMIT-TO ( AFFILCOUNTRY , " Zaire " ) OR LIMIT-TO ( AFFILCOUNTRY , " Djibouti " ) OR LIMIT-TO ( AFFILCOUNTRY , " Egypt " ) OR LIMIT-TO ( AFFILCOUNTRY , " Equatorial Guinea " ) OR LIMIT-TO ( AFFILCOUNTRY , " Eritrea " ) OR LIMIT-TO ( AFFILCOUNTRY , " Ethiopia " ) OR LIMIT-TO ( AFFILCOUNTRY , " Gabon " ) OR LIMIT-TO ( AFFILCOUNTRY , " Gambia " ) OR LIMIT-TO ( AFFILCOUNTRY , " Ghana " ) OR LIMIT-TO ( AFFILCOUNTRY , " Guinea " ) OR LIMIT-TO ( AFFILCOUNTRY , " Guinea Bissau " ) OR LIMIT-TO ( AFFILCOUNTRY , " Ivory Coast " ) OR LIMIT-TO ( AFFILCOUNTRY , " Cote d'Ivoire " ) OR LIMIT-TO ( AFFILCOUNTRY , " Kenya " ) OR LIMIT-TO ( AFFILCOUNTRY , " Lesotho " ) OR LIMIT-TO ( AFFILCOUNTRY , " Liberia " ) OR LIMIT-TO ( AFFILCOUNTRY , " Libya " ) OR LIMIT-TO ( AFFILCOUNTRY , " Libia " ) OR LIMIT-TO ( AFFILCOUNTRY , " Jamahiriya " ) OR LIMIT-TO ( AFFILCOUNTRY , " Jamahiryia " ) OR LIMIT-TO ( AFFILCOUNTRY , " Madagascar " ) OR LIMIT-TO ( AFFILCOUNTRY , " Malawi " ) OR LIMIT-TO ( AFFILCOUNTRY , " Mali " ) OR LIMIT-TO ( AFFILCOUNTRY , " Mauritania " ) OR LIMIT-TO ( AFFILCOUNTRY , " Mauritius " ) OR LIMIT-TO ( AFFILCOUNTRY , " Ile Maurice " ) OR LIMIT-TO ( AFFILCOUNTRY , " Morocco " ) OR LIMIT-TO ( AFFILCOUNTRY , " Mozambique " ) OR LIMIT-TO ( AFFILCOUNTRY , " Moçambique " ) OR LIMIT-TO ( AFFILCOUNTRY , " Namibia " ) OR LIMIT-TO ( AFFILCOUNTRY , " Niger " ) OR LIMIT-TO ( AFFILCOUNTRY , " Nigeria " ) OR LIMIT-TO ( AFFILCOUNTRY , " Rwanda " ) OR LIMIT-TO ( AFFILCOUNTRY , " Sao Tome " ) OR LIMIT-TO ( AFFILCOUNTRY , " Senegal " ) OR LIMIT-TO ( AFFILCOUNTRY , " Seychelles " ) OR LIMIT-TO ( AFFILCOUNTRY , " Sierra Leone " ) OR LIMIT-TO ( AFFILCOUNTRY , " Somalia " ) OR LIMIT-TO ( AFFILCOUNTRY , " South Africa " ) OR LIMIT-TO ( AFFILCOUNTRY , " Sudan " ) OR LIMIT-TO ( AFFILCOUNTRY , " South Sudan " ) OR LIMIT-TO ( AFFILCOUNTRY , " Swaziland " ) OR LIMIT-TO ( AFFILCOUNTRY , " Tanzania " ) OR LIMIT-TO ( AFFILCOUNTRY , " Tanganyika " ) OR LIMIT-TO ( AFFILCOUNTRY , " Zanzibar " ) OR LIMIT-TO ( AFFILCOUNTRY , " Togo " ) OR LIMIT-TO ( AFFILCOUNTRY , " Tunisia " ) OR LIMIT-TO ( AFFILCOUNTRY , " Uganda " ) OR LIMIT-TO ( AFFILCOUNTRY , " Zambia " ) OR LIMIT-TO ( AFFILCOUNTRY , " Zimbabwe " ) OR LIMIT-TO ( AFFILCOUNTRY , " Northern Rhodesia " ) OR LIMIT-TO ( AFFILCOUNTRY , " Southern Rhodesia " ) OR LIMIT-TO ( AFFILCOUNTRY , " Gold Coast " ))  **AND NOT**  (Guinea pig* OR "Guinea pig*" OR Aspergillus niger OR "Aspergillus niger")  **AND**  ( LIMIT-TO ( EXACTKEYWORD , "Human" ) OR LIMIT-TO ( EXACTKEYWORD , "Humans" )) |

**Supplementary Table S2a. Additional summary of African Gut Microbiome studies.**

| **Title** | | **Aim** | **Participant Information** | **Ethnicity** | **Region, District or Town** | **Rural vs Urban** | **Name of Journal** | **Accessibility** | **First Author Affiliation (Country)** | **Last Author Affiliation (Country)** | **Data Availability** | **Reference** |
| --- | --- | --- | --- | --- | --- | --- | --- | --- | --- | --- | --- | --- |
|  | **GUT** | | | | | | | | | | | |
| Salt in stools is associated with obesity, gut halophilic microbiota and Akkermansia muciniphila depletion in humans | | To investigate whether fecal salinity could be associated with age, gender, geography, obesity, and with gut microbiota alteration both by 16S rRNA-targeted metagenomics and culturomics | Algeria (n=5), Senegal (n=25), Mali (n=62), Amazonia (n=18), France (n=22), French Polynesia (n=7), Saudi Arabia (n=25). Tuaregs from the Algerian desert. Healthy 0 to 59-month-old children from an ongoing study on severe acute malnutrition (healthy controls) in Bamako, Mali. Individuals included in a cohort longitudinal study on malaria in 2 villages of Southern Senegal (Dielmo & N’Diop). | Tuaregs (Algeria) | Africa (Bamako and surroundings villages, Mali; Dielmo and N’Diop, rural Southern Senegal; Tuareg people of the Algerian desert, Algeria), Europe (France, Marseille), South America (French Guiana, Amazonian Forest), Saudi Arabia (Urban Jeddah and nomadic population from southwestern Saudi Arabia), and Oceania (French Polynesia, Iles du Vent). | Rural and Urban | International Journal of Obesity | Not open | Aix Marseille University, France | Aix Marseille University, France | NA | [60] |
| Population structure of human gut bacteria in a diverse cohort from rural Tanzania and Botswana . | | To survey the gut bacteria of seven non-industrialized populations from Tanzania and Botswana. | Adults population from rural populations in Tanzania (n=60), Botswana (n=54) that practiced diverse modes of subsistence (such as pastoralism, agropastoralism, hunting and gathering, and mixed hunting and gathering), and individuals living in an urban US city (Philadelphia, PA) (n=12). (Bantu (n=26), Herero (n=8), San (n=20), Hadza (n=25), Sandawe (n=12), Maasai (n=12), Burunge (n=11)). | Burunge, Sandawe, Maasai, and Hadza (Tanzania) and Bantu, Herero, and San (Botswana) | Arusha and Shinyanga regions surrounding Lake Eyasi (Hadza hunter-gatherers), the Northern Ngorongoro district (Maasai), the Kondoa district in Central Tanzania (Burunge, Sandawe), Western/Northwestern regions (Botswana), Philadelphia region (USA) | Rural (Tanzania, Botswana) and Urban (USA) | Genome Biology | Open | University of Pennsylvania, USA | University of Pennsylvania, USA | NCBI SRA accession number: PRJNA395034 | [48] |
| Acute diarrhea in west African children: diverse enteric viruses and a novel parvovirus genus | | To conduct viral metagenomic analysis of feces of children from Burkina Faso with acute diarrhea. | Children less than 5 years of age with acute diarrhea report. | NA | Ouagadougou | NA | Journal of Virology | Open | University of California at San Franscico & Blood System Research Institute, USA | University of California at San Franscico & Blood System Research Institute, USA | NA | [61] |
| Indirect Effect of Azithromycin Use on the Intestinal Microbiome Diversity of Untreated Children: A Randomized Trial | | To evaluate whether receipt of azithromycin in one sibling confers changes to the intestinal microbiome in an untreated sibling compared with placebo in a randomized controlled trial. | Households were eligible for inclusion in the study if they had 2 or 3 children between the ages of 6 and 59 months. Children were monitored at baseline and 5 days after the last antibiotic dose (after treatment). | NA | Nouna district | Rural | Open Forum Infectious Diseases | Open | Francis I. Proctor Foundation & University of California, USA | Francis I. Proctor Foundation & University of California, USA | NA | [62] |
| Impact of diet in shaping gut microbiota revealed by a comparative study in children from Europe and rural Africa | | To compare the fecal microbiota of European children and that of children from Burkina Faso. | 1-6-year-old healthy children from Italy (n=15) and Burkina Faso (n=15). Nine boys and six girls from each cohort. | Mossi (Burkina Faso) | Boulpon district of Nanoro, Boulkiemde province (Burkina Faso) and Florence (Italy) | Rural (Burkina Faso) and Urban (Italy) | Proceedings of the National Academy of Sciences (PNAS) | Open | University of Florence, Italy | University of Florence, Italy | EBI-ENA SRA accession number: ERP000133 | [63] |
| Effect of Commonly Used Pediatric Antibiotics on Gut Microbial Diversity in Preschool Children in Burkina Faso: A Randomized Clinical Trial | | To determine the effect of 3 commonly used pediatric antibiotics on the intestinal microbiome in healthy preschool children. | Children aged 6–59 months were randomized to a 5-day course of 1 of 3 antibiotics, including amoxicillin (n=27), azithromycin (n=30), cotrimoxazole (n=29), or placebo (n=29). | NA | Nouna district | Rural | Open Forum Infectious Diseases | Open | Francis I. Proctor Foundation & University of California, USA | Francis I. Proctor Foundation & University of California, USA | NA | [64] |
| Use of shotgun metagenomics for the identification of protozoa in the gut microbiota of healthy individuals from worldwide populations with various industrialization levels | | To compared metagenomics-based and targeted methods of detection for two common protozoan genera: *Blastocystis* and *Entamoeba.* | Healthy individuals (23 hunter-gatherers, 24 farmers and 21 individuals from a fishing population; 40 males and 28 females) aged 26 to 78 years (median of 50 years) and living in seven different villages situated in Southwest Cameroon. | NA | Southwest Cameroon | Rural | PLoS One | Open | CNRS—MNHN—Univ Paris Diderot—Sorbonne Paris Cité, France | CNRS—MNHN—Univ Paris Diderot—Sorbonne Paris Cité, France | EBI-ENA accession number: ERS2539904-ERS2539960, BioProject: ID PRJEB27005 | [65] |
| Variation in rural African gut microbiota is strongly correlated with colonization by Entamoeba and subsistence | | To better understand the relative influence of diet, host genetics and parasitism on human gut microbiota composition and structure. | 26 females and 38 males 26 to 78 years, 20 hunter-gatherers, 24 farmers, 20 individuals from fishing populations, 20 Pygmy and 44 Bantu. | Pygmy and Bantu | Bidou, Makouré, Bandevouri, Ndtoua, Afan Essokié, Akak and Ebodié (7 villages in Southern Cameroon) | Rural | PLoS Genetics | Open | University of Minnesota, USA | University Denis Diderot, France | Raw Sequence through MG-RAST project ID: mgp 15238 | [66] |
| Gut Virome Analysis of Cameroonians Reveals High Diversity of Enteric Viruses, Including Potential Interspecies Transmitted Viruses | | To investigate the gut virome of humans from Cameroon and to further determine if bat viruses are possible causative agents of gastrointestinal infections in humans. | Humans (almost all with gastroenteritis symptoms) between 0 and 89 years of age with different degrees of bat contact. 131 from Kumba and 90 from Lysoka. Subjects between age 0 and <3 years (n = 80), 3 and <20 (n = 63), 20 and <60 (n = 65), and 60 and older (n = 13). | NA | Kumba, Lysoka (Southwest region of Cameroon) | NA | mSphere | Open | Rega Institute for Medical Research & KU Leuven-University of Leuven, Belgium | Rega Institute for Medical Research & KU Leuven-University of Leuven, Belgium | NCBI SRA accession number: PRJNA491626 | [67] |
| Stunted childhood growth is associated with decompartmentalization of the gastrointestinal tract and overgrowth of oropharyngeal taxa | | To describe small intestinal bacterial overgrowth (SIBO) composition in stunted children and suggests that oropharyngeal taxa may be an important contributing factor to the pathophysiology of pediatric environmental enteropathy (PEE). | Female and male children (aged 2–5 years) with moderate and severe stunting with small intestinal bacterial overgrowth. 404 fecal samples, 57 gastric samples, and 46 duodenal samples. Central African Republic (n=251), Madagascar (n=153). | NA | Bangui (Central African Republic), or Antananarivo (Madagascar) | NA | Proceedings of the National Academy of Sciences (PNAS) | Open | Institut Pasteur, France | Institut Pasteur & Collège de France, France | EBI-ENA accession numbers: PRJEB27868 and ERP110005 (ERS2620873–ERS2621413) | [68] |
| Gut microbiome of coexisting BaAka Pygmies and Bantu reflects gradients of traditional subsistence patterns | | To explore how the gut microbiome varies with human adaptation in traditional societies with distinct diets and environment. | 28 BaAka (also included seven female partners) and 29 Bantu. | BaAka pygmies and Bantu | Dzanga Sangha Protected Areas | Rural | Cell Reports | Open | University of Minnesota & J. Graig Venter Institute, USA | University of Minnesota, USA | MG-RAST accession number: 16608 | [69] |
| Plasticity in the Human Gut Microbiome Deﬁes Evolutionary Constraints. | | To understand the forces that shape the primate gut microbiome with reference to human populations by expanding the comparative analysis of variation among gut microbiome compositions and their primate hosts, including 9 different primate species and 4 human groups characterized by a diverse set of subsistence patterns. | 9 different primate species and 4 human groups characterized by a diverse set of subsistence patterns (n= 448 samples). Hunter-gatherers (The BaAka, n=28) and traditional agriculturalists (The Bantu, n=29) from the Dzanga Sangha Protected Areas, Central African Republic, and western researchers working at the same field site for 3 to 6months (n=5). 392 fecal samples of anthropoid primates were collected in different locations across Africa, Central America, and Mexico, including African great apes (mountain gorillas [n=48], western lowland gorillas [n=191], and Central African chimpanzees [n=10]), Old World monkeys (olive baboons [n=4], geladas [n=7], agile mangabeys [n=10], and vervets [n=23]), as well as New World monkeys (black howlers [n=33] and captive tufted capuchins [n =4]). | The BaAka and The Bantu | The Dzanga Sangha Protected Areas (Central African Republic) | Rural | American Society for Microbiology | Open | University of Minnesota, USA | University of Colorado, USA | MG-RAST accession number: 16608, mgp6321, mgp13961, and mgp89894, NCBI SRA accession number: SRP065516 | [70] |
| The microbiome and resistome of chimpanzees, gorillas, and humans across host lifestyle and geography | | To show that the gut microbial taxa, microbial gene family composition, and resistomes of great apes and humans are more related by host lifestyle than geography. | Human samples were collected from humans living outside of the national park boundaries, the village of Bomassa, and from humans working within the park boundaries. Wild ape fecal samples were collected in the Goualougo Triangle region of Nouabalé-Ndoki National Park in the Republic of the Congo. | NA | Bomassa | Rural | The ISME Journal | Not open | Washington University School of Medicine, USA | Washington University School of Medicine & Washington University in St. Louis, USA | NCBI SRA accession number: PRJNA539933 | [71] |
| Evolution in fecal bacterial/viral composition in infants of two central African countries (Gabon and Republic of the Congo) during their first month of life | | To analyze the different steps involved in the infant gut bacterial/virus seeding and to monitor the changes of the gut microbiota community in the early days of life. | Healthy newborns at the first month of life, 9 from Congo (including the twins) and 6 from Gabon and one fecal sample collected from each mother (14). | NA | Angondjé (Gabon) and Brazzaville (Republic of Congo) | NA | PLoS One | Open | Institut de Recherche pour le Développement (IRD), France | Institut de Recherche pour le Développement (IRD), France | Open Science Framework (OSF) DOI: doi.org/10.17605/OSF. IO/G7T2U | [72] |
| Investigations on the interplays between Schistosoma mansoni, praziquantel and the gut microbiome | | To investigate potential worm-induced dysbacteriosis of the gut microbiota and to assess whether a specific microbiome profile could influence praziquantel response. | Schistosoma mansoni infected and uninfected (2–15 years) children. From each infected child one pre-treatment, one 24-hour and one 21-day follow-up sample after administering 60 mg/kg praziquantel or placebo, were collected. | NA | Azaguié (Southern Côte d’Ivoire) | Rural | Parasit Vectors | Open | Swiss Tropical and Public Health Institute & University of Basel, Switzerland | Swiss Tropical and Public Health Institute & University of Basel, Switzerland | Available upon request | [73] |
| Metagenomic diagnostics for the simultaneous detection of multiple pathogens in human stool specimens from Côte d'Ivoire: a proof-of-concept study | | To prove that shotgun metagenomics approach provides useful information on the diverse composition of intestinal pathogens and antimicrobial resistance profiles in human stool samples. | 2 males and 2 females aged 1 to 34 years with persistent diarrhea and abdominal pain. | NA | Dabou, West of Abidjan | Rural and Peri-urban | Infection, Genetics and Evolution | Not-open | Institute for Plant Production Sciences IPS, Federal Office for Civil Protection, Swiss Tropical and Public Health Institute, & University of Basel, Switzerland | Swiss Tropical and Public Health Institute & University of Basel, Switzerland | NA | [74] |
| Gut microbiome alterations in patients with stage 4 hepatitis C | | To explore the composition of the gut microbiomes of Hepatitis C virus (HCV) patients at Beni-Suef Emergency Hospital, (Beni-Suef, Egypt) in comparison to healthy individuals from the same city. | Adult males aged 21-65 with 6 cases with stage 4 Hepatitis C virus and 8 controls. One sample from cases group were excluded. | NA | Beni-Suef | NA | BMC Gut Pathogens | Open | Beni-Suef University, Egypt | Cairo University, Egypt | NCBI SRA accession number: SAMN0586401, SAMN05386428 BioProject: PRJNA328966 | [75] |
| Effect of radiotherapy on the gut microbiome in pediatric cancer patients: a pilot study | | To analyze ten fecal samples from three pediatric cancer patients, suffering from rhabdomyosarcoma near their pelvic region, and two healthy individuals. | Children (patients and healthy individuals (not exposed to chemotherapeutic drugs or antibiotics)) were males with age range between 3.5 and 7 years old. All patients were diagnosed with rhabdomyosarcoma in the pelvic region. They were treated with a complex treatment protocol including chemotherapy. Ten fecal samples were collected from three cancer patients and two healthy controls. | NA | Cairo | NA | PeerJ | Open | Zewail City of Science and Technology & American University in Cairo, Egypt | Zewail City of Science and Technology & AGERI, ARC, Egypt | NCBI SRA accession number: PRJNA545788 | [76] |
| New Insights on Obesity and Diabetes from Gut Microbiome Alterations in Egyptian Adults | | To report notable differences in gut microbiome in adult patients with obesity and diabetes in a population sample from the El-Sharkia governate in north-east Egypt. | Male and female subjects, 5 controls with a normal body mass index, without obesity or diabetes, 25 Obese adults without diabetes, 5 adults with diabetes who are not obese, and 25 adults who are both obese and diabetic. | NA | El-Sharkia governate in North-eastern Egypt | NA | OMICS A Journal of Integrative Biology | Not open | Port Said University, Egypt | Suez Canal University, Egypt | NCBI SRA accession number: PRJNA484095 | [77] |
| Differences in gut metabolites and microbial composition and functions between Egyptian and U.S. children are consistent with their diets | | To discover possible relationships between human gut microbiota and consumed diets. | Healthy pre-adolescent and adolescent male volunteers from Egypt (n=28) and USA (n=14). | NA | Giza (Egypt) and Dayton, OH (USA) | Urban | mSystems | Open | Wright State University, USA | Wright State University, USA | NCBI SRA Bioproject ID: PRJNA314988 and MG-RAST for egkHLT, 4552772.3 and 4552773.3, for uskHLT, 4552774.3 and 4552775.3 | [78] |
| Viral species richness and composition in young children with loose or watery stool in Ethiopia | | To evaluate potential associations between enteric virome composition, species richness and stool consistency. | Children aged 0 to 5 years. The median age of children with stool samples was 36 months. | NA | Amhara region | Rural | BMC Infectious Diseases | Open | University of California San Francisco, USA | University of California San Francisco, USA | Open Science Framework repository | [79] |
| Enteric virome of Ethiopian children participating in a clean water intervention trial | | To describe the eukaryotic viral genomes in the feces of Ethiopian children participating in a clean water intervention trial. | Samples were collected from children aged 0–5 years with a mean age of 2.7 years from 14 villages in the Amhara region of Ethiopia, half of which received a new hand-dug water well. | NA | Goncha Siso Enese district (woreda) of Amhara | Rural | PLoS One | Open | Blood Systems Research Institute & University of California San Francisco, USA | Blood Systems Research Institute & University of California San Francisco, USA | GenBank accession number: SRP120619 | [80] |
| Household composition and the infant fecal microbiome: The INSPIRE study | | To examine relationships between infant fecal microbiome (IFM) diversity/composition and infants' household size, number of siblings, and number of other household members. | Infants enrolled in the study were between the ages of 2 weeks and 5 months, described by their mothers to be healthy, showing no symptoms of illness in the week prior to prior to enrollment. Mothers were also required to be healthy, showing no symptoms of acute illness in the week prior to enrollment, not experiencing breast infection or atypical breast pain, and having not received antibiotics in the month prior to enrollment. Ethiopia (rural n=39, urban n=32), Kenya (n=40), The Gambia( rural n=36, urban n=38), Ghana (n=32), Peru (n=42), Spain (n=37), Sweden (n=23), USA (n=41). | Nordic (Sweden), Unspecified ethnicity (Southeastern Washington), Hispanic, (Northwestern Idaho), Sidama (Ethiopian), Mandika (Gambia), Krobo ot Dangme (Ghana), multiethnic (Kenya) | Madrid, Zaragoza, Huesca, and Vizcaya (Spain), Helsingborg (Sweden), Lima (Peru), Southern California, Southeastern Washington and Northwestern Idaho (USA), Hawassa (Ethiopia), Bakauarea and West Kiang region (Gambia), Southeastern Ghana, and Nakuru (Kenya) | Peri-urban (Peru), Rural, and Urban (Ethiopia, The Gambia, Kenya, and Ghana) | The American Journal of Physical Anthropology | Not open | Washington State University, USA | Washington State University, USA | NA | [81] |
| Growth and morbidity of Gambian infants are influenced by maternal milk oligosaccharides and infant gut microbiota | | To determine whether human milk oligosaccharides (HMO) content and composition are influenced by seasonal environmental changes, and whether changes in HMO composition are associated with protection from morbidity, improved growth, and with the intestinal microbiome. | Mother-infants’ pairs with infant fecal samples collected at 4, 16, and 20 weeks postpartum. | NA | NA | NA | Scientific Reports | Open | University of California, USA | University of California, USA | Qiita Database under Study ID: 10297 and in EBI-ENA accession number: ERP017462 | [82] |
| Decreased microbial co-occurrence network stability and SCFA receptor level correlates with obesity in African-origin women | | To examine the ecological co-occurrence network topology of the gut microbiota as well as the relationship of short chain fatty acids (SCFAs) with obesity in rural Ghanian and urban Americans. | 13 lean and 37 obese US women, and 29 lean and 21 obese Ghanaian women. Participants were excluded from participating in the original METS study if they self-reported an infectious disease, including HIV, are pregnant or lactating or have any condition which prevented the individual from participating in normal physical activities. | NA | NA | Rural (Ghana) and Urban (USA) | Scientific Reports | Open | Loyola University Chicago, USA | University of Illinois at Chicago & Jesse Brown Veterans Affairs Medical Center, USA | NA | [83] |
| The Impact of Anthelmintic Treatment on Human Gut Microbiota Based on Cross-Sectional and Pre- and Postdeworming Comparisons in Western Kenya | | To utilize stool samples collected longitudinally from an area where A. lumbricoides and N. americanus are endemic in order to examine the relationship between these soil-transmitted helminths (STH) and the human gut microbiota. | Individuals aged 3 to 84 years (median age, 51 years) who are infected with STH. Pretreatment and post-treatment samples from the same individuals were compared. | NA | 5 villages Western Kenya (Siangwe, Siaka, Sang’alo, Nasimbo, and Ranje village) | Rural | mBio | Open | National Institutes of Health, USA | National Institutes of Health, USA | NCBI SRA accession number: PRJNA510835 | [84] |
| Iron fortification adversely affects the gut microbiome, increases pathogen abundance and induces intestinal inflammation in Kenyan infants | | To determine the effect of high and low dose in-home iron fortification on the infant gut microbiome and intestinal inflammation. | 6-month-old infants consuming home-fortified maize porridge daily for 4 months, no maternal or infant chronic disease, mother more than 15 years. | NA | Msambweni County (Southern coastal Kenya) | Rural | Gut | Open | ETH Zurich, Switzerland | ETH Zurich, Switzerland | NA | [85] |
| Longitudinal Analysis of Infant Stool Bacteria Communities Before and After Acute Febrile Malaria and Artemether-Lumefantrine Treatment | | To determine to what degree Plasmodium infections and antimalarial treatment affect human gut microbiota. | Ten children were selected on the basis of stool samples having been collected before (n = 27) or after (n = 17) a malaria episode and without antibiotics having been administered between collections. | NA | Kilifi | NA | Journal of the Infectious Diseases | Open | University of Louisville, USA | University of Louisville, USA | NA | [86] |
| Microbiome sharing between children, livestock and household surfaces in western Kenya | | To investigate the role of animal and household contact on gut microbiome of children in rural livestock-owning households. | 143 children less or equal to 5 years old with at least one cow and at least one chicken at home. Samples from cooking area (n=69), living space (n=34), cows (n=125) and chicken (n=36). | NA | Western Kenya | Rural | PLoS One | Open | University of Washington & Washington State University, USA | University of Washington, USA | NCBI SRA accession number: SRP090785 | [87] |
| Molecular definition of vaginal microbiota in East African commercial sex workers | | To investigate whether vaginal microbiology was associated with HIV-exposed seronegative (HESN) or HIV-seropositive (HIV+) status in a cohort of commercial sex workers. | HIV-exposed seronegative women (16), HIV-negative controls (16) and HIV-seropositive women (12). | NA | Nairobi | NA | Applied and Environmental Microbiology | Open | University of Manitoba, Canada | University of Manitoba & Public Health Agency of Canada, Canada | NA | [88] |
| Prebiotic galacto-oligosaccharides mitigate the adverse effects of iron fortification on the gut microbiome: a randomised controlled study in Kenyan infants | | To evaluate the efficacy and safety of a new micronutrient powder (MNP) formula with prebiotic galacto-oligosaccharides (GOS) combined with a low dose (5 mg/ day) of highly bioavailable iron. | Healthy Kenyan infants aged 6.5–9.5 months, 10 infants dropped out and not included in the study. | NA | Southern coastal Kenya | NA | Gut | Open | ETH Zurich, Switzerland | ETH Zurich, Switzerland | NA | [89] |
| Maternal Human Milk Oligosaccharide Profile Modulates the Impact of an Intervention with Iron and Galacto-Oligosaccharides in Kenyan Infants | | To determine Human milk oligosaccharides profiles and the secretor/non-secretor phenotype of lactating Kenyan mothers and investigate their effects on the maternal and infant gut microbiota, and on the infant response to a fortification intervention with 5 mg iron (2.5 mg as sodium iron ethylenediaminetetraacetate and 2.5 mg as ferrous fumarate) and 7.5 g prebiotic galacto-oligosaccharides. | Healthy infants 6.5–9.5 months of age who had received no vitamin and mineral supplements 8 weeks and no antibiotics 10 weeks prior to study entry, who had a hemoglobin (Hb) >70 g/L, and Z-scores weight-for-age (WAZ) and weight-for-length (WLZ) both >−3. | NA | Southern coastal, Kenya | NA | Nutrients | Open | ETH Zurich, Switzerland | ETH Zurich, Switzerland | NA | [90] |
| Iron-containing micronutrient powders modify the effect of oral antibiotics on the infant gut microbiome and increase post-antibiotic diarrhoea risk: a controlled study in Kenya | | To evaluate the effect of antibiotics on the infant gut microbiome and diarrhoea when given with or without iron-containing micronutrient powders (MnPs). | Four groups of community-dwelling infants (n=28; aged 8–10 months) received either: (a) antibiotics for 5 days and iron-MnPs for 40 days ; (B) antibiotics and no-iron-MnPs; (c) no antibiotics and iron-MnPs; or (D) no antibiotics and no-iron-MnPs. We collected a faecal samples before the first antibiotic dose (D0) and after 5, 10, 20 and 40 days (D5–D40). | NA | Southern coastal, Kenya | NA | Gut | Not open | ETH Zurich, Switzerland | ETH Zurich, Switzerland | NA | [91] |
| Diarrhea in young children from low-income countries leads to large-scale alterations in intestinal microbiota composition | | To survey the intestinal microbiota in a subset of GEMS (Global Enterics Multicenter Study) cases and controls. | Children under 5 years with moderate-to-severe diarrhea (n=508) and diarrhea-free controls (n=484). 269 of them from The Gambia, 305 from Kenya, 212 from Mali and 206 from Bangladesh. | NA | NA | NA | BMC Genome Biology | Open | University of Maryland, USA | University of Maryland, USA | NCBI archive under project: PRJNA234437 | [92] |
| Iron in micronutrient powder promotes an unfavorable gut microbiota in Kenyan infants | | To assess the effects of iron fortification in the form of micronutrient powder (MNP) on the gut microbiome and inflammation status in six-month-old, non- or mildly-anemic Kenyan infants. | Participants, 6 months of age, non-mildly anemic, predominantly-breastfed in malaria-endemic area. 13 received micronutrient powder (MNP) containing 12.5 mg iron; another 13 received MNP containing no iron; and 7 received Placebo. | NA | Western Kenya | Rural | Nutrients | Open | University of Colorado School of Medicine, USA | University of Colorado School of Medicine, USA | NA | [93] |
| Differential human gut microbiome assemblages during soil-transmitted helminth infections in Indonesia and Liberia | | To determine the association between the soil-transmitted helminths (STHs) and the gut microbiome. | No subjects with acute infections or other acute illnesses were included in the study as they would be not eligible for anthelminthic mass drug administration. Participants were from remote areas of north-western Liberia (Foya District, Lofa County; n= 68) or in coastal eastern Liberia (Harper District, Maryland County; n= 30) and Indonesia (n=304). | NA | Remote areas of North-western (Foya District, Lofa County) or in coastal Eastern (Harper District, Maryland County) (Liberia) | Rural (Remote areas) | Microbiome | Open | Washington University, USA | Washington University & Washington University School of Medicine, USA | NCBI SRA accession number: PRJNA407815 and SAMN07688522 to SAMN07688545 | [94] |
| Gut microbiota in Malawian infants in a nutritional supplementation trial | | To examine whether two forms of lipid-based nutrient supplements or a micronutrient-fortified corn-soya blend were associated with development of the gut microbiota in Malawian infants, to assess the microbiota profiles at the age of 6 and 18 months and to follow the changes during the 12-month period. | 6-month old, underweight and stunted infants’ resident in either of the two communities (117 of them were girls). | NA | Lungwena and Malindi | Rural | Tropical Medicine International Health | Open | University of Tampere School of Medicine, Finland & Duke-NUS Graduate Medical School, Singapore | University of Tampere School of Medicine & Tampere University Hospital, Finland | NA | [95] |
| Environmental exposures and child and maternal gut microbiota in rural Malawi | | To investigate whether gut microbiota composition in young children and their mothers is associated with different environmental exposures in rural Malawi. | Samples from children and mothers participating in a nutrition intervention trial were collected at 1, 6, 12, 18, and 30 months (children) and at 1 month (mothers) after birth. | NA | Mangochi district | Rural | Paediatric and Perinatal Epidemiology | Open | Tampere University, Finland | Tampere University & Tampere University Hospital, Finland | EBI-ENA accession number: PRJEB29433 | [96] |
| A Prospective Study on Child Morbidity and Gut Microbiota in Rural Malawi | | To investigate whether morbidity predicts gut microbiota composition in Malawian children and whether microbiota predicts subsequent morbidity. | Samples were collected from birth until children reached 18 months of age and fecal samples were collected at 6, 12, 18, and 30 months of age. If a child had diarrhea, no fecal sample was collected, and the visit was postponed by 2 weeks. | NA | Mangochi district (Southern Malawi) | Rural | Journal of Pediatric Gastroenterology and Nutrition | Not open | Tampere University, Finland | Tampere University & Tampere University Hospital, Finland | EBI-ENA accession number: PRJEB29433 | [97] |
| The effect of dietary resistant starch type 2 on the microbiota and markers of gut inflammation in rural Malawi children | | To test hypothesis that gut inflammation will be reduced with dietary supplementation with resistant starch in rural Malawian children. | 3-5 years old boys (n=8) and girls (n=10) with stunted growth. | NA | Machinga District | Rural | BMC Microbiome | Open | Washington University, USA | Washington University, Baylor College of Medicine, Houston, USA & University of Malawi, Malawi | NA | [98] |
| Environmental enteric dysfunction and the fecal microbiota in Malawian children | | To determine which bacterial taxa were associated with environmental enteric dysfunction (EED). | 12-23 month old healthy rural children (n=47 boys and n=34 girls). | NA | Machinga and Nsanje district | Rural | American Journal of Tropical Medicine and Hygiene | Open | Washington University, USA | Washington University, United States Department of Agriculture/Agricultural Research Service Childrens Nutrition Research Centre, USA & University of Malawi, Malawi | NA | [99] |
| Design and application of a novel twoamplicon approach for defining eukaryotic microbiota | | To design a novel two-amplicon surveillance tool, based on sequencing regions of ribosomal RNA genes and their internal transcribed spacers. | Children suffering from Severe Acute Malnutrition (SAM). | NA | NA | NA | Microbiome | Open | The Hospital for Sick Children & University of Toronto, Canada | The Hospital for Sick Children & University of Toronto, Canada | NCBI SRA accession number: PRJNA483794 | [100] |
| Gut DNA viromes of Malawian twins discordant for severe acute malnutrition | | To characterize gut microbial community development in healthy versus malnourished infants/children. | 8 twin pairs concordant for healthy growth, 12 twin pairs discordant for severe acute malnutrition within the first 30 months of post-natal life. Samples collected from mothers and older siblings of infants. | NA | Southern villages of Malawi | Rural | Proceedings of the National Academy of Sciences (PNAS) | Open | Washington University, USA & Universidad de los Andes, Colombia | Washington University, USA | EBI-ENA accession number: PRJEB9818 | [101] |
| Gut microbiomes of Malawian twin pairs discordant for kwashiorkor | | To investigate the role of the gut microbiome in childhood nutrition. | Twin pairs younger than 3 years old regardless of their health status. Nine same-gender twin pairs who remained well-nourished and 13 same-gender twin pairs who became discordant for kwashiorkor. | NA | Makhwira, Mintodo, M'biza, Chamba, and Mayaka (Southern region of Malawi) | Rural | Science | Open* | Washington University in St. Louis, USA | Washington University in St. Louis, USA | 16S rRNA & shotgun in EBI accession number: ERP001928, ERP001911 | [102] |
| Human gut microbiome viewed across age and geography | | To examine how gut microbiomes differ among human populations by characterizing bacterial species and bacterial gene content in fecal samples. | Parents and siblings, 115 individuals (34 families) from Malawi; 100 individuals (19 families) from Venezuela, and 316 individuals (98 families) from the USA. A subset of 110 fecal samples were used in shotgun 454 pyrosequencing. | Guahibo Amerindians (Venezula) | Chamba, Makwhira, Mayaka and Mbiza, rural communities (Malawi), The greater metropolitan areas of St Louis, Philadelphia and Boulder (USA), and Platanillal and Coromoto, near Puerto Ayacucho in the Amazonas State (Venezula) | Rural (Malawi and Venezula) and Urban (USA) | Nature | Open* | Washington University School of Medicine, USA | Washington University School of Medicine, USA | MG-RAST accession number qiime: 850 and for 16S V4 Illumina and qiime: 621 for shotgun | [103] |
| Stool microbiota composition is associated with the prospective risk of Plasmodium Falciparum infection | | To identify the composition of the gut bacterial communities in Malian children and adult prior to an intense P. falciparum transmission season. | Samples collected just prior to the six-month malaria season from healthy individuals aged 3 months to 25 years (106 females and 94 males). | 87% Bambara | Kalifabougou | Rural | BMC Genomics | Open | J. Craig Venter Institute, USA | J. Craig Venter Institute, USA | NCBI SRA accession number: PRJNA285808 and in MG-RAST Project number: 4793 | [104] |
| Blastocystis Colonization Is Associated with Increased Diversity and Altered Gut Bacterial Communities in Healthy Malian Children | | To assess the influence of Blastocystis on the gut bacterial communities in healthy children. | Healthy children from a prospective malaria cohort study with a mean age of 8 years, consisting of 154 (51.3%) females and 146 (48.7%) males (p=0.97). Children with Blastocystis-colonized (n=147) and with Blastocystis-noncolonized (n=149). | Dogon, Peuhl, Bobo, Tamacheck, Sonrhai, Bellah, Minyanka, Dafing, Soninké, Senoufo, Mossi, Haoussa, Gana, Bambara, and Malinke | Bandiagara | NA | Microorganisms | Open | Aix Marseille Université & IHU Méditerranée Infection, France | Aix Marseille Université & IHU Méditerranée Infection, France | NA | [105] |
| Direct Detection of Shigella in Stool Specimens by Use of a Metagenomic Approach | | To conﬁrm qPCR-based detection of Shigella via a metagenomics approach. | Children with diarrhea from three countries: Mali, Mozambique, and India (nine samples each). | NA | NA | NA | Journal of Clinical Microbiology | Open | University of Virginia, USA | University of Virginia, USA | NCBI SRA accession numbers: SRX3008896 to SRX3008922 and BioProject: PRJNA394687 | [106] |
| Stool microbiome reveals diverse bacterial ureases as confounders of oral urea breath testing for Helicobacter pylori and Mycobacterium tuberculosis in Bamako, Mali | | To evaluate the sensitivity and specificity of oral Urea Breath Test to diagnose active tuberculosis and H. pylori in patients who were co-infected with H. pylori and Mycobacterium tuberculosis, mono-infected with Mycobacterium tuberculosis, and mono-infected with H. pylori. | 80 participants 18 years and older in total but only 10 healthy participants with no clinical or laboratory evidence of either Mycobacterium tuberculosis or H. pylori infection but with a positive oral Urea Breath Test (18 years old or older) used for microbiome study. | NA | Bamako | NA | Journal of Breath Research | Open* | Université des Sciences, des Techniques et des Technologies de Bamako, Mali & Johns Hopkins University, USA | Johns Hopkins University, USA | NA | [107] |
| Gut microbiome of Moroccan colorectal cancer patients | | To compare the stool microbiome of Moroccan cancer patients with healthy individuals. | Male and female adults age from 20 to 80 years (11 colorectal cancer patients and 12 healthy subjects). | NA | Oriental region (Northeastern area) and Casablanca | NA | Medical Microbiology and Immunology | Open | Mohammed V University in Rabat & University Mohammed Premier, Morocco & University of North Carolina, USA | University Mohammed Premier & National Center Scientific and Technological Research, Morocco | NA | [3] |
| Gut microbial diversity in antibiotic-naive children after systemic antibiotic exposure: a randomized controlled trial | | To evaluate the effects of azithromycin on the gut microbiome diversity of children from an antibiotic-naive community in Niger. | Healthy children aged 1-60 months, 31 boys and 49 girls, randomized to receive one-time dose of azithromycin (n=40) and placebo (n=40). | NA | Dosso Region | Rural and agricultural | Clinical Infectious Diseases | Open | University of California San Francisco, USA | University of California San Francisco, USA | NA | [108] |
| Mass Azithromycin Distribution and Community Microbiome: A Cluster-Randomized Trial | | To assess whether 2 biannual mass treatments of preschool children affect the community’s gut microbiome at 6 months after the last distribution. | Children aged 1–60 months in the Dossa region of Niger were randomized at the village level to receive a single dose of azithromycin or placebo every 6 months. | NA | Dossa region (Niger) | NA | Open Forum Infectious Diseases | Open | University of California San Francisco, USA | University of California San Francisco, USA | NA | [109] |
| Gut microbiome alteration in MORDOR I: a community-randomized trial of mass azithromycin distribution | | To examine the gut microbiome of preschool children from 30 Nigerien communities randomized to either biannual azithromycin or placebo. | Preschool children aged 1–59 months from 30 villages in Niger. These villages were randomly selected from the same pool as the mortality MORDOR I trial in Niger and randomized to receive either one oral administration of azithromycin Mass Drug Administration or placebo every 6 months for 24 months. | NA | 30 villages in the Dosso region | Rural | Nature Medicine | Open | University of California, USA | University of California, USA | NCBI SRA accession number: PRJNA549968 | [110] |
| High-throughput sequencing of pooled samples to determine community-level microbiome diversity | | To randomly pool a number of microbiome samples from the same community into one sample before sequencing to estimate community-level microbiome diversity. | Children aged 1-60 months were assigned to receive oral azithromycin (n = 40) or placebo (n = 40) 5 days before their rectal sample collection. | NA | Sarkin Yara Koira | NA | Annals of Epidemiology | Open | Francis I. Proctor Foundation & University of California, USA | Francis I. Proctor Foundation & University of California, USA | NA | [111] |
| Gut bacteria missing in severe acute malnutrition, can we identify potential probiotics by culturomics? | | To describe the gut microbiota of patients with kwashiorkor and healthy children using both metagenomics and culturomics and to identify prokaryote candidate probiotics that restore a healthy gut microbiota in malnourished children. | 10 severely undernourished children with nutritional oedema (kwashiorkor), 6 from Senegal and 4 from Niger and 5 healthy children (no wasting, no stunting, and not underweight), 3 from Senegal and 2 from Niger. | NA | Dakar (Senegal) and Niamey (Niger) | NA | Frontiers in Microbiology | Open | Aix-Marseille Université, France & Université Abdou Moumouni de Niamey, Niger | Aix-Marseille Université, France | NA | [112] |
| Gut Microbiome Profiles Are Associated With Type 2 Diabetes in Urban Africans | | To describe microbiome composition and identify differences between Type 2 Diabetes and controls. | Participants (n=98 cases, n=193 controls), mean age (54.3 (13.3) for controls and 59.7 (10.4) for cases. 97% of cases were on treatment with 38.8% on metformin only, 5.1% on sulfonylurea only, 49% on metformin and sulfonylurea, and 4.1% on combinations of anti-diabetes medication. 3.1% were treatment-naïve. | NA | Ibadan | Urban | Frontiers in Cellular and Infection Microbiology | Open | National Institutes of Health, USA | National Institutes of Health, USA | NCBI SRA accession number: PRJNA607849 | [113] |
| Impact of a Nomadic Pastoral Lifestyle on the Gut Microbiome in the Fulani Living in Nigeria | | To determine the taxonomic and predicted functional proﬁle of the gut microbiome of a hitherto unstudied human community. | 28 healthy Fulani (13 males and 15 females, mean age = 19 years, SD = 16) and 22 healthy Jarawa (non-Fulani, 9 males and 13 females, mean age = 29 years, SD = 18). The volunteers were healthy and had not received antibiotics for at least 1 month before sampling. | The Fulani tribe and the Jarawa tribe | Pabamanshanu village, Plateau State, North-Central Nigeria | Rural and Semi-urban | Frontiers in Microbiology | Open | University of Ibadan, Nigeria | Medical University of Graz & Theodor Escherich Laboratory for Microbiome Research, Austria | NA | [114] |
| Infant and Adult Gut Microbiome and Metabolome in Rural Bassa and Urban Settlers from Nigeria | | To assess the subsistence-related variation of the human gut microbiome at a fine resolution for two of the main dimensions of microbiome variation, age and geography. | 18 Bassa (9 infants aged <3 years and 9 individuals aged 3–60 years) and 30 urban Nigerians (12 infants aged <3 years; 18 adults aged 5–75 years). | Bassa, Hausas, Igbos, Yorubas, Ebira, and Others | Bassa rural agriculturalists, and urban individuals from four state capitals (Ilorin, Abeokuta, Ado Ekiti, and Ibadan) and the Nigerian capital city (Abuja) | Rural and Urban | Cell Reports | Open | University of Ibadan, Nigeria | University of Bologna, Italy | MG-RAST accession number: mgp83994 | [115] |
| The bacteriome at the onset of type 1 diabetes: a study from four geographically distant African and Asian countries | | To characterize the stool bacteriome in children after onset of Type 1 Diabetes along with age- and place-matched control subjects from four geographically distant African and Asian countries. | Children and adolescents with median age of 11.8 years (interquartile range 7.8 - 14.0) shortly after T1D onset (Azerbaijan (n=19), Jordan (n=20), Nigeria (n=14), Sudan (n=18)) and 103 matched control subjects of similar place of residence (Azerbaijan (n=32), Jordan (n=20), Nigeria (n=13), Sudan (n=38)) and age (11.3 years, interquartile range 8.1 - 13.7). | NA | NA | NA | Diabetes Research and Clinical Practice | Not open | Charles University in Prague and University Hospital Motol, Czech Republic | University of Khartoum & Sudan Childhood Diabetes Center, Sudan | NCBI SRA accession number: PRJNA445932 | [116] |
| Rectal microbiota among HIV-uninfected, untreated HIV, and treated HIV-infected in Nigeria | | To evaluate the rectal microbiota among three groups: HIV-uninfected, untreated HIV, and ART-treated HIV. | Men who have sex with men (55 HIV-uninfected, 41 with untreated HIV infection, and 34 ART-treated HIV). | NA | Abuja | NA | AIDS | Open* | University of Maryland, USA | TRUST/RV368 group, USA | NA | [117] |
| Anal Microbial Patterns and Oncogenic Human Papillomavirus in a Pilot Study of Nigerian Men Who Have Sex with Men at Risk for or Living with HIV | | To cluster anal microbiota and define microbial patterns associated with biological, clinical, and behavioural correlates among Nigerian men who have sex with men (MSM) living with or at risk for HIV. | Men who have sex with men. Approximately half of the participants were HIV uninfected (47%), a third had untreated HIV (31%), and one-fifth were taking ART for their HIV infections (22%). | NA | Abuja | NA | AIDS Research and Human Retroviruses | Open | University of Maryland School of Medicine, USA | University of Maryland School of Medicine, USA | NA | [118] |
| Evidence of the megavirome in humans | | To describe the detection of sequences related to Mimivirus and Marseillevirus in the gut microbiota from a young Senegalese man. | Healthy 20-year-old Senegalese man. | NA | NA | Rural | Journal of Clinical Virology | Not-open | Aix-Marseille Université & Centre Hospitalo-Universitaire Timone, France | Aix-Marseille Université & Centre Hospitalo-Universitaire Timone, France | NA | [119] |
| Microbial culturomics: paradigm shift in the human gut microbiome study | | To introduce culturomics as a major complement to metagenomics in the study of the human gut microbiome, by combining many well-known and new methods (sequencing, culture). | 2 young lean males in Senegal and 1 obese French individual. | NA | NA | Rural (Senegal) | Clinical Microbiology and Infection | Open | Aix-Marseille Université, France | Aix-Marseille Université, France | NCBI SRA accession number: SRA049748 | [120] |
| Dysbiosis Signatures of Fecal Microbiota in South African Infants with Respiratory, Gastrointestinal, and Other Diseases | | To determine the association between the fecal microbiota diversity of infants with different disease conditions, and vitamin A supplementation, antibiotic, and deworming therapies. | Infants (mean age, 16 ± 8 months; 17 male and 17 female) were hospitalized for respiratory (n = 16 [47%]), gastrointestinal (n = 11 [33%]), and other diseases (n = 7 [20%]). | NA | East London | NA | Journal of Pediatrics | Not open | University of Pretoria, South Africa | University of Pretoria, South Africa | NA | [121] |
| Atopic dermatitis and food sensitization in South African toddlers: Role of fiber and gut microbiota | | To examine whether toddlers with Atopic Dermatitis and control toddlers from Cape Town have different microbiomes in terms of bacterial richness and diversity. | 12-36 months old black African children, 29 with atopic dermatitis and 9 healthy controls. | NA | Cape Town | NA | Annals of Allergy, Asthma and Immunology | Open | Rush University, USA | University of Cape Town, South Africa | NA | [122] |
| A prospective cohort analysis of gut microbial co-metabolism in Alaska Native and rural African people at high and low risk of colorectal cancer | | To investigate how the diet of Alaskan Native people affects the composition and function of the gut microbiota and related metabolic markers that are associated with increased colorectal cancer risk and to compare findings with rural people from South Africa, who have a low-fat, high-fiber diet. | Healthy male and female Alaskan Natives or Rural Africans without history of cancer, bowel diseases, HIV, diabetes and antibiotic use within the past 12 weeks, and regional resident for at least the past 5 years. A total of 32 Alaska Native participants and 21 healthy Rural African individuals. | NA | KwaZulu-Natal (South Africa), Anchorage, Utqiagvik (Alaska) | Rural (South Africa) | American Journal of Clinical Nutrition | Not open | University of Pittsburgh, USA & German Institute of Human Nutrition Potsdam-Rehbruecken, Germany | University of Pittsburgh, USA | Available upon request | [123] |
| Diet, microbiota, and microbial metabolites in colon cancer risk in rural Africans and African Americans | | To examine the hypothesis that the influence of diet on colon cancer risk is mediated by the microbiota through their metabolites. | Healthy volunteers of either sex aged 50-65 years, 12 African Americans at high risk of colon cancer and 12 native Africans (South Africans) with low risk of colon cancer. | NA | Rural area outside Empangeni, KwaZulu-Natal, (South Africa), and Pittsburgh (USA) | Rural (South Africa) | The American Journal of Clinical Nutrition | Open | University of Pittsburgh, USA | University of Pittsburgh, USA | NA | [124] |
| Feeding-Related Gut Microbial Composition Associates With Peripheral T-Cell Activation and Mucosal Gene Expression in African Infants | | To interrogate the mechanisms underlying the protective effect of exclusive breastfeeding. | Infants at birth with mean gestational age of 38.9 weeks and mean birth weight of 3.2 kg. All infants were exclusively breastfed (EBF) at birth, but only 43.5% and 20% remained EBF at 6 or 14 weeks of age, respectively. | NA | Khayelitsha, Western Cape | NA | Clinical Infectious Diseases | Open | University of Washington Schools of Medicine and Public Health, USA | University of Washington Schools of Medicine and Public Health & Seattle Children’s Research Institute, USA & University of Cape Town, South Africa | NA | [125] |
| HIV-exposure, early life feeding practices and delivery mode impacts on faecal bacterial profiles in a South African birth cohorT | | To characterize meconium and early life faecal bacterial profiles of HIV-exposed and -unexposed infants enrolled in a South African birth cohort study | Stool and meconium specimens were collected from 90 mothers and 107 infants at birth, and from a subset of 72 and 36 infants at 4–12 and 20–28 weeks of age, respectively. | TC Newman (primarily a mixed ancestry population) and Mbekweni (primarily a black African population) | TC Newman and Mbekweni | Peri-urban | Scientific Reports | Open | University of Cape Town, South Africa | University of Cape Town, South Africa | NCBI SRA accession number: PRJNA356372 and BioSamples SAMN06131047 to SAMN06131374 | [126] |
| Metagenome sequencing of the Hadza hunter-gatherer gut microbiota | | To describe the gut microbiota resistome configuration in Hadza as compared with Italians and obtain a picture of antibiotic resistant gene expression in a human population with little or no antibiotic exposure. | 27 healthy Hadzas aged 8-70 years and 11 healthy Italians aged 20-40 years. | Hadza | Dedauko and Sengele camps (Northwestern Tanzania) and around Bologna (Italy) | Rural (Tanzania) and Urban (Italy) | Current Biology | Open | University of Bologna, Italy | University of Bologna, Italy | NCBI SRA accession number: SRP056480, Bioproject ID: PRJNA278393 and Shotgun in MG-RAST project: 8810 | [32] |
| Gut microbiome of the Hadza hunter-gatherers | | To explore variation in gut microbiota and to understand how these bacteria may have co-evolved with humans. | 27 Hadza volunteers (8-70 years) and 16 adults (20-40 years) healthy volunteers from Italy. | Hadza | Dedauko and Sengele camps (Northwestern Tanzania) and the greater Bologna metropolitan area (Italy) | Rural (Tanzania) and Urban (Italy) | Nature Communications | Open | Max Planck Institute for Evolutionary Anthropology, Germany | University of Nevada, USA | MG-RAST database under project ID 201, ENA accession number: ERP000133 | [33] |
| Seasonal cycling in the gut microbiome of the Hadza hunter-gatherers of Tanzania | | To perform an in-depth, longitudinal analysis of the Hadza hunter-gatherer microbiome, to provide insight into the dynamics of a diverse gut microbiota in a non-industrial, non-urban setting. | 188 Samples collected during a 12-month period spanning five sub seasons from healthy Hadza hunter-gatherers. Data from 20 Hadzas from a previous Hadza study and 71 U.S. residents from the Human Microbiome project (HMP). 35 samples across the seasons selected for shotgun metagenomic sequencing. | Hadza (Tanzania) | Hadza near Lake Eyasi (Tanzania) | Rural | Science | Not-open | Stanford University, USA | Stanford University, USA | NCBI SRA under the project IDs: PRJNA392012, PRJNA392180 | [127] |
| Randomized open-label pilot study of the influence of probiotics and the gut microbiome on toxic metal levels in Tanzanian pregnant women and school children | | To investigate the potential for probiotic-supplemented yoghurt to lower heavy metal levels in at-risk populations of pregnant women and children in Tanzania. | 44 healthy children aged 6 to 10 years and 60 pregnant women in their first trimester. | NA | Mwanza | NA | mBio | Open | Lawson Health Research Institute & The University of Western Ontario, Canada | Lawson Health Research Institute & The University of Western Ontario, Canada | NCBI SRA BioProject: PRJNA244107 | [21] |
| Altered virome and bacterial microbiome in human immunodeficiency virus-associated acquired immunodeficiency syndrome | | To examine the effect of HIV on the enteric virome and bacterial microbiome in a well-characterized Ugandan cohort. | Men and women aged 24-49 years, divided into 82 cases (42 subjects with untreated HIV infection and 40 location-matched samples from subjects on long-term anti-retroviral therapy for more than 5 years) and 40 controls (HIV uninfected subjects). | NA | Mbarara region | NA | Cell Host and Microbe | Open | Washington University, USA | Washington University, USA | EMBL-EBI accession number: PRJEB9524 | [128] |
| Gut microbiota in children hospitalized with oedematous and non-oedematous severe acute malnutrition in Uganda | | To assess whether gut microbiota composition differed between Ugandan children suffering from either oedematous or non-oedematous malnutrition. | 6-24 months children admitted for treatment of severe acute malnutrition (SAM). | NA | Kampala | NA | PLoS Neglected Tropical Diseases | Open | University of Copenhagen, Denmark | University of Copenhagen, Denmark | EBI-ENA accession number: PRJEB10006, sample accession numbers: ERS799842-ERS799928 | [129] |
| The impact of storage conditions on human stool 16S rRNA microbiome composition and diversity | | To address the impact of different preservative methods, time-to-freezing at ambient tropical temperatures, and stool heterogeneity on stool microbiome diversity and composition under real-life physical environments found in resource-limited ﬁeldwork conditions. | Children aged 12–14 years. | NA | Mayuge District | NA | PeerJ | Open | University of Glasgow, UK | University of Glasgow, UK | EBI-ENA accession number: PRJEB32925 | [130] |
| Child development, growth and microbiota: follow-up of a randomized education trial in Uganda | | To examine the effects of an education package delivered to mothers in rural Uganda on their children’s development, growth and gut microbiota at 36 months of age. | Mother-infant pairs in the parental study were randomized to the intervention (n = 263) or the control (n = 248) group. The infants were 6-8 months. | NA | The Kisoro and Kabale districts of South-Western Uganda | Rural | Journal of Global Health | Open | University of Oslo, Norway | Kristiania University College, Norway | NCBI SRA accession number: SUB4476421 | [131] |
| Comparison of the distal gut microbiota from people and animals in Africa | | To analyze the relationship between bacterial taxa of the human gut microbiota and those in the gut microbiota of domestic and semi-wild animals. | Five mother and child pairs, children below or 5 years; 6 adult males, 30-45 years old, whose relationships to the mother and child pairs were unknown; 6 chimpanzees wild-born semi-captive, 2-5 years and 5 grazing cattle. | NA | Bugoto, Mayuge District | Rural | PLoS One | Open | Animal Health and Veterinary Laboratories Agency, UK | University of Bristol, UK | NCBI SRA (accession numbers to follow) | [132] |
| Composition of Gut Microbiota of Children and Adolescents With Perinatal Human Immunodeficiency Virus Infection Taking Antiretroviral Therapy in Zimbabwe | | To investigate composition of gut microbiota in HIV-infected and -uninfected children and assessed associations between gut microbiota and patient characteristics. | Children aged 6–16 years. 149 HIV-infected participants with chronic lung disease, 28 HIV-infected participants without chronic lung disease, 103 HIV-uninfected participants. All HIV-infected participants on ART for at least 6 months with 89% on cotrimoxazole prophylaxis. | NA | Harare | NA | The Journal of Infectious Diseases | Open | UiT - The Arctic University of Norway & University Hospital of North Norway, Norway | UiT - The Arctic University of Norway & University Hospital of North Norway, Norway | EBI-ENA accession number: PRJEB32077 | [133] |
| Cotrimoxazole reduces systemic inflammation in HIV infection by altering the gut microbiome and immune activation | | To determine whether cotrimoxazole has anti-inflammatory effects, and to elucidate underlying mechanisms. | Stool samples were collected at weeks 84 and 96 after randomization from a subgroup of children in Zimbabwe who had been receiving ART and once-daily cotrimoxazole prophylaxis (200 mg of sulfamethoxazole/ 40 mg of trimethoprim, 400 mg of sulfamethoxazole/80 mg of trimethoprim, or 800 mg of sulfamethoxazole/160 mg of trimethoprim for body weight 5 to 15, 15 to 30, or >30 kg, respectively). Stool for whole-metagenome sequencing (stop, n=36; continue, n=36). | NA | NA | NA | Science translational medicine | Open | Queen Mary University of London, UK | Queen Mary University of London & MRC Clinical Trials Unit at University College London, UK & Zvitambo Institute for Maternal and Child Health Research, Zimbabwe | NA | [134] |
| Differences in the faecal microbiome in Schistosoma haematobium infected children vs. uninfected Children | | To determine if there were differences in the structure (diversity and abundance) of the faecal microbiome between children infected with S. haematobium and uninfected children and to investigate the effects of Praziquantel treatment on the structure of the gut microbiome. | Schistosome infected and uninfected children (male and female), aged 6 months to 13 years old. | NA | Murewa district | NA | PLOS Neglected Tropical Diseases | Open | University of Warwick, UK | University of Warwick, UK | NA | [135] |

**Supplementary Table S2b. Additional summary of African Urogenital Microbiome studies.**

| **Title** | **Aim** | **Participant Information** | **Ethnicity** | **Region, District or Town** | **Rural vs Urban** | **Name of Journal** | **Accessibility** | **First Author Affiliation (Country)** | **Last Author Affiliation (Country)** | **Data Availability** | **Reference** |
| --- | --- | --- | --- | --- | --- | --- | --- | --- | --- | --- | --- |
| **UROGENITAL** | | | | | | | | | | | |
| Altered vaginal microbiota are associated with perinatal mother-to-child transmission of HIV in African women from Burkina Faso | To determine whether perinatal mother-to-child transmission (MTCT) is associated with the vaginal microbiotas of HIV-infected mothers. | HIV-1-infected pregnant women at 36-38 weeks of gestation, 10 who transmitted and 54 who did not transmit to infant were included in study. | NA | Bobo-Dioulasso | NA | Journal of Acquired Immune Deficiency Syndromes | Not-open | University of Colorado, USA | University of Colorado School of Medicine & Department of Veterans Affairs Medical Center, USA | GenBank accession number: JF461543-JF487783 | [136] |
| Microbial diversity of genital ulcer disease in men enrolled in a randomized trial of male circumcision in Kisumu, Kenya | To define the etiology of genital ulcer disease in men participating in the Kenyan trial and examine how medical male circumcision affects genital ulcer disease etiology. | Men aged 18-24 years who are uncircumcised, HIV negative and have been sexually active in the last 12 months. | NA | Kisumu | NA | PLoS One | Open | University of Illinois at Chicago, USA | Rush University, USA | NA | [137] |
| An effective intervention to reduce intravaginal practices among HIV-1 uninfected Kenyan women | To investigate the potential impact of intravaginal practice intervention on the composition of the vaginal microbiota, epithelial integrity, vaginal inflammatory cytokines and activated immune cells as key biomarkers of vaginal ecology relevant to HIV-1 acquisition risk. | HIV-uninfected women, not pregnant, 18-45 years old, asymptomatic, sexually active (at least one episode of sex in the last year), and not self-identifying as a female sex workers. 42 engages in intravaginal practice and 30 do not (controls). | NA | Mombasa | NA | AIDS Research and Human Retroviruses | Open* | New York University, USA | New York University, USA | NA | [138] |
| Association of high-risk sexual behaviour with diversity of the vaginal microbiota and abundance of Lactobacillus | To compare the vaginal microbiota of women engaged in high-risk sexual behaviour (sex work) with women who are not engaged in high-risk sexual behaviour. | HIV-negative women >18 years, not pregnant, not breastfeeding and not post-menopausal. 19 of them were non-sex worker controls and 48 were female sex workers with <3 years of sex work. | NA | Pumwani | NA | PLoS One | Open | McMaster University, Canada | McMaster University, Canada | NA | [139] |
| Medroxyprogesterone acetate alters the vaginal microbiota and microenvironment in women and increases susceptibility to HIV-1 in humanized mice | To determine whether depot medroxyprogesterone acetate affected vaginal microbiota diversity. | Women were included if >18 years old, pre-menopausal, sex workers, willing to undergo pelvic exams, intact uterus and cervix, in good health and negative for STIs (gonorrhoea, chlamydia, Trichomonas vaginalis, syphilis, HIV), negative for yeast infection. | NA | Nairobi | Urban | Disease Models and Mechanisms | Open | McMaster University, Canada | McMaster University, Canada | NCBI Gene Expression Omnibus (GEO) accession number: GSE138971 | [140] |
| Comparative analysis of the vaginal microbiome of pregnant women with either Trichomonas vaginalis or Chlamydia trachomatis | To determine the vaginal microbiome profiles of three groups of pregnant women (Trichomonas vaginalis-infected, Chlamydia trachomatis-infected, and controls, i.e. women with none of the two infections) attending antenatal care at Kilifi, County Hospital, Kenya, and compare our results with previous findings. | Women aged 18– 45 years with Trichomonas vaginalis (TV, N = 18) were compared to those with Chlamydia trachomatis (CT, N = 14) infection, and to controls (women negative for TV, CT and bacterial vaginosis, N = 21) | NA | Kilifi | NA | PLoS One | Open | Centre for Geographic Medicine Research-Coast & Pwani University, Kenya & Ghent University, Belgium | Centre for Geographic Medicine Research-Coast & Pwani University, Kenya & University of Oxford, UK | EBI-ENA accession number: PRJEB25935 | [141] |
| Evaluation of the association between the concentrations of key vaginal bacteria and the increased risk of HIV acquisition in African women from five cohorts: a nested case-control study | To evaluate the association between the concentrations of specific vaginal bacteria and increased risk of HIV acquisition in African women. | Participants were selected from three cohorts of Eastern and Southern African women (female sex workers, pregnant and post-partum women, and women in serodiscordant relationships). Mombasa cohort (n=7 controls and n=7 cases who were aged 16 years or older, HIV seronegative, and self-identified as exchanging sex for cash or in-kind payment were included), Mama Salama Study (n=20 controls and n=20 cases who are 14 years or older, pregnant, and HIV-seronegative), the Partners Pre-Exposure Prophylaxis Study (PrEP) (n=28 controls and n=28 cases who are 18 years or older, HIV-seronegative, and had an HIV-seropositive male partner aged 18 years or older). | NA | Mombasa (Eastern and Southern Africa), Mama Salam Cohorts (Kenya), and the PrEP Study (Kenya and Uganda) | NA | The Lancet Infectious Diseases | Not-open | University of Washington, USA & University of Nairobi, Kenya | University of Washington, USA | NCBI SRA (In Progress) | [142] |
| Semen bacterial concentrations and HIV-1 RNA shedding among HIV-1-seropositive kenyan men | To determine the association of semen bacterial concentrations with semen HIV-1 RNA levels in a cohort of HIV-1-positive Kenyan men. | HIV-1–seropositive men (29 to 35 years) from a high-risk cohort including men with multiple sexual partners and men who had sex with men. | NA | NA | NA | Journal of Acquired Immune Deficiency Syndromes | Open | University of Washington, USA | University of Washington, USA & University of Nairobi, Kenya & Oxford University, UK | NCBI SRA accession number: SRP073630 | [143] |
| Impact of Standard Bacterial Vaginosis Treatment on the Genital Microbiota, Immune Milieu, and Ex Vivo Human Immunodeficiency Virus Susceptibility. | To assess the impact of oral metronidazole treatment on the genital immune parameters of HIV acquisition risks in Kenyan women with BV. | Women aged 19-46 years (median=26 years) who were sexually active. | NA | NA | NA | Clinical Infectious Diseases | Open | University of Toronto, Canada | University of Toronto, Canada | NCBI SRA accession number: PRJNA407441 | [144] |
| Prevalent high-risk HPV infection and vaginal microbiota in Nigerian women | To evaluate the association between High-risk Human Papillomavirus (hrHPV) and the vaginal microbiome. | Women above 18 years regardless of HIV and HPV status. | NA | Abuja | NA | Epidemiology and Infection | Open | Institute of Human Virology,Nigeria & University of Cambridge, UK | University of Maryland School of Medicine & Harvard School of Public Health, USA | NA | [145] |
| Mycoplasma hominis and Mycoplasma genitalium in the vaginal microbiota and persistent High-risk Human Papillomavirus infection | To examine associations between prevalence and persistence of *Mycoplasma spp.* in the vaginal microbiota, and prevalent as well as persistent High-risk Human Papillomavirus (hrHPV) infections. | Women 18 years or older who had a history of vaginal sexual intercourse, were not pregnant and had no history of hysterectomy. | NA | Abuja | NA | Frontiers in Public Health | Open | University of Maryland School of Medicine, USA | Institute of Research Virology, Nigeria | NA | [146] |
| The microbiome in urogenital schistosomiasis and induced bladder pathologies | To determine the microbiome features and changes in urine during urogenital schistosomiasis and induced bladder pathologies. | 36 Males and 34 Females ages between 15 and 65. | NA | Eggua (South-Western Nigeria) | Rural | PLOS Neglected Tropical Diseases | Open | University of Ibadan, Nigeria | National Center for Cell Science, India | NCBI SRA accession number: SRP094688 | [147] |
| Impact of oral metronidazole treatment on the vaginal microbiota and correlates of treatment failure | To evaluate the impact of a standard course of oral metronidazole treatment (500 mg twice per day for 7 days) on the vaginal microbiota of Rwandan bacterial vaginosis patients using microscopy and 16S rRNA gene sequencing, and to evaluate correlates of treatment failure. | HIV-negative, nonpregnant women, aged 18-45 years, and in good overall physical and mental health but at high risk of bacterial vaginosis. | NA | Kigali | NA | American Journal of Obstetrics and Gynecology | Open | University of Liverpool, UK | University of Liverpool, UK & Utrecht University, The Netherlands | Available upon request | [148] |
| Comparison of lower genital tract microbiota in HIV-infected and uninfected women from Rwanda and the US | To assess the geographic differences in types of genital microbiota between HIV-infected and uninfected women from Rwanda and the United States. | 40 women from Rwanda (18 HIV Infected and 22 HIV-uninfected) and 46 women from USA (36 HIV Infected and 10 HIV-uninfected). | NA | NA | NA | PLoS One | Open | Johns Hopkins Bloomberg School of Public Health, USA | Rush University Medical Center, USA | NA | [149] |
| A multi-platform metabolomics approach identifies highly specific biomarkers of bacterial diversity in the vagina of pregnant and non-pregnant women | To demonstrate that the vaginal metabolome is driven by bacterial diversity, and identify biomarkers of clinical bacterial vaginosis that can be reproduced in a blinded validation cohort. | Premenopausal women between the ages of 18 and 55 years, 67 pregnant and 64 non-pregnant. | NA | Mwanza | NA | Scientific Reports | Open | The University of Western Ontario, Canada | The University of Western Ontario, Canada | NCBI SRA BioProject ID: PRJNA289672 | [31] |
| Pilot assessment of probiotics for pregnant women in Rwanda | To examine receptivity and compliance for orally administered probiotic capsules containing Lactobacillus rhamnosus GR-1 and Lactobacillus reuteri RC-14 in pregnant women and assess any initial side effects or changes to the vaginal microbiome. | Pregnant women between the ages of 18 and 55 were randomly assigned to receive probiotic or placebo capsules for one month. The mean age of the 20 women who received probiotics and 18 women who received placebo for one month was 26.3 and 27.6 years respectively. | NA | Nyamata district | NA | PLoS One | Open | Lawson Health Research Institute, Canada & Lerner Research Institute, USA | Lawson Health Research Institute & The University of Western Ontario, Canada | NA | [150] |
| Human papillomavirus infection and cervical dysplasia in HIV-positive women: potential role of the vaginal microbiota | To assess the associations between microbiological markers of vaginal dysbiosis and incident/cleared/type-swap/persistent high-risk human papillomavirus (hrHPV) infection; and incident/cured/cleared/persistent high-grade cervical intraepithelial neoplasia (CIN2þ) while controlling for persistent hrHPV infection. | HIV-infected women, aged 25–50 years without previous treatment for cervical cancer, previous hysterectomy, and being pregnant or less than 8 weeks postpartum. | NA | Johannesburg | Urban | AIDS | Not open | University of Liverpool, UK & Utrecht University, The Netherlands | London School of Hygiene and Tropical Medicine, UK | NA | [148] |
| The cervical microbiota in reproductive-age South African women with and without human papillomavirus infection | To investigate the baseline structure of cervical microbiota of reproductive-age black South African women and determine their (microbiota) associations with the participants’ demographic, sociobehavioural, and clinical information. | Reproductive-age, HIV-seronegative women aged 18-44, menstruating or pregnant at the time of sampling. | NA | Gugulethu, Cape Town | NA | Papillomavirus Research | Open | University of Cape Town, South Africa | University of Cape Town, South Africa | NCBI SRA accession number: PRJNA473351 | [151] |
| Factors associated with the composition and diversity of the cervical microbiota of reproductive-age Black South African women: a retrospective cross-sectional study | To examine potential associations of human papillomavirus (HPV) infection with the cervical microbiota. | Black South African women, 37 (42.5%) were HPV-positive. Among the HPV positive women, 51.4% (19/37) had single HPV infections. A total of 45.9% (17/37) and 81.1% (30/37) of the women were infected with LR- and HR-HPV types, respectively. Among these women, 27.0% (10/ 37) were infected with both LR- and HR-HPVs. A majority of the women had normal cervical cytology (75.6% (62/82)) while few had experienced vaginal discharge (15.4% 12/78)) or genital ulceration (2.3% (2/ 87)) in the last 6 months. A total of 43.7% (38/87) of the women had findings suggestive of Bacterial Vaginosis on their Pap smear results. Approximately one-third of the women (32.2%) were currently cigarette smokers. | NA | Gugulethu, Cape Town | NA | PeerJ | Open | University of Cape Town, South Africa | University of Cape Town, South Africa | NCBI SRA accession numbers: SRX4103412–SRX4103416 and BioProject ID: PRJNA472137 (SRP148486) | [152] |
| Metagenomic analysis reveals a rich bacterial content in high‐risk prostate tumors from African men | To find if pathogenic microbes may be contributing, at least in part, to high‐risk prostate cancer presentation within Africa and in turn the observed ethnic disparity. | African patients from South Africa (n=6), while Australian patients (all European descent) from New South Wales (n=16). All patients received antibiotics, Ciproxin before biopsy (African) or Ceftriaxone during surgery (Australian). | NA | NA | NA | Prostate | Open | Zhejiang University School of Medicine, China | The Kinghorn Cancer Centre & University of New South Wales Sydney & University of Sydney, Australia & University of Pretoria, South Africa | NCBI SRA accession numbers: SRP119289 and PRJNA412953 | [153] |
| Endocervical and vaginal microbiota in South African adolescents with asymptomatic Chlamydia trachomatis infection | To compare the microbiota of the lateral vaginal wall and endocervix, and asses associations with Chlamydia trachomatis infection in South African adolescent. | Women 16–22-year-old women from a low income, high population density community in Cape Town, South Africa. Females were enrolled if they were HIV-negative, in good health, not pregnant or menstruating at the time of sampling and if they had not unprotected sex or douched in the last 48 hours, nor taken antibiotics in the prior two weeks. | NA | Cape Town | Urban | Scientific Reports | Open | University of Cape Town, South Africa | University of Cape Town, South Africa & Seattle Children’s Research Institute & University of Washington, USA | NA | [154] |
| Cervicovaginal bacteria are a major modulator of host inflammatory responses in the female genital tract | To determine the contribution of cervicovaginal microbiota to genital inflammation in an asymptomatic HIV negative cohort of young South African women. | HIV negative black South African women aged 18-23 years. | NA | KwaZulu-Natal | NA | Immunity | Open | Massachusetts General Hospital & Harvard Medical School, USA | Massachusetts General Hospital, USA | NA | [155] |
| High rates of bacterial vaginosis and Chlamydia in a low-income, high-population-density community in Cape Town | To describe the vaginal microbiota and prevalence of sexually transmitted infections in HIV-negative South African women. | HIV-negative women aged 16-22 years from a low-income and high-population-density community in Cape Town. | 99% Xhosa and 1% Zulu | Cape Town | NA | Suid-Afrikaanse Tydskrif vir Natuurwetenskap en Tegnologie | Open | University of Cape Town, South Africa | University of Cape Town, South Africa & University of Washington, USA | NA | [23] |
| Microbial Composition Predicts Genital Tract Inﬂammation and Persistent Bacterial Vaginosis in South African Adolescent Females | To unbiasedly identify microbial factors that best predict genital inﬂammation and/or increased HIV target cell activation in adolescent females in an unbiased manner. | Black women, 16- to 22-year-old, HIV-negative women from low-income, high-population-density communities in Cape Town (n=90) and Johannesburg (n=78), not pregnant not menstruating at the time of sampling | NA | Cape Town and Johannesburg | NA | Infection and Immunity | Open | University of Cape Town, South Africa | University of Cape Town, South Africa & Seattle Children's Research Institute & University of Washington, USA | EBI-ENA Bioproject number: PRJEB15497 | [24] |
| Vaginal microbiota varies by geographical location in South African women | To compare the vaginal microbiota of 16–22-year-old black HIV-negative South African women from two low income high population density communities; one in Cape Town (CPT) and the other Johannesburg (JHB.) | Black, 16–22-year-old HIV-negative women were recruited from low-income, high population density communities in Cape Town (n=102) and Johannesburg (n=79). Young women were enrolled if they were HIV-negative, in general good health, not pregnant or menstruating at the time of sampling, and if they had not unprotected sex or douched in the last 48 hours. | Xhosa and Others | Cape Town and Johannesburg | NA | South African Journal for Science and Technology | Open | University of Cape Town, South Africa | University of Cape Town, South Africa & University of Washington, USA | NA | [25] |
| Vaginal bacteria modify HIV tenofovir microbicide efficacy in African women | To investigate whether vaginal microbiota modulated tenofovir gel microbicide efficacy in the CAPRISA (Centre for the AIDS Program of Research in South Africa) 004 trial. | HIV-negative women. | NA | KwaZulu-Natal | NA | Science | Not-open | University of Washington, USA | Public Health Agency of Canada & University of Manitoba, Canada & Karolinska University Hospital, Sweden | NA | [156] |
| Lactobacillus-deficient cervicovaginal bacterial communities are associated with increased HIV acquisition in young South African Women | To present a comprehensive characterization of the genital bacterial and viral microbiome in healthy young women in sub-Saharan Africa, and to identify a significant association of distinct cervicovaginal bacterial communities and specific bacterial taxa with HIV acquisition. | 31 HIV infected and 205 uninfected healthy women aged 18–23 years old. | NA | Umlazi | NA | Immunity | Open | Massachusetts General Hospital & Harvard Medical School, USA | Massachusetts General Hospital & Harvard Medical School, USA | EBI-ENA project number: PRJEB14858 | [157] |
| Relationship between the Cervical Microbiome, HIV Status, and Precancerous Lesions | To gain an understanding of the differences in the cervical bacterial community composition as a function of cervical cytology grade and HIV status. | Female patients (HIV positive (n=41), HIV negative (n=103)), 18 years and above (mean 37 years), undergoing cervical cancer screening. Participants were not pregnant or menstruating. | NA | Dar es Salaam, Chalinze, Bagamoyo | Rural and Urban | mBio | Open | University of Nebraska-Lincoln, USA | University of Nebraska-Lincoln, USA | NA | [158] |
| Deep sequencing of the vaginal microbiota of women with HIV | To define (1) the microbial communities of women living with HIV, (2) their association with the vaginal pH and (3) their relation with the Amsel criteria and Nugent scoring system commonly used to diagnose bacterial vaginosis. | HIV infected women not yet on ART aged 18-45 years with 39 diagnosed with bacterial vaginosis. | NA | Mwanza | NA | PLOS One | Open | Lawson Health Research institute, Canada & University Medical Centre Rottenham, The Netherlands | Lawson Health Research institute & The University of Western Ontario, Canada | GenBank accession number: HM585291-HM585350 | [30] |
| Male circumcision significantly reduces prevalence and load of genital anaerobic bacteria | To assess the effect of male circumcision on the genital microbiota using absolute abundance. | HIV negative men, 15-49 years, 79 circumcised (intervention group) and 77 uncircumcised (control group). | NA | Rakai | NA | mBio | Open | Translational Genomics Research Institute & Northern Arizona University & Johns Hopkins University, USA | Translational Genomics Research Institute & George Washington University, USA | NA | [26] |
| Penile microbiota and female partner bacterial vaginosis in Rakai, Uganda | To test the hypothesis that having a female partner with bacterial vaginosis (BV) increases BV-associated bacteria in uncircumcised men. | Uncircumcised HIV-negative men, 15 to 49 years of age. | NA | Rakai | NA | mBio | Open | Translational Genomics Research Institute & Northern Arizona University & Johns Hopkins University, USA | Johns Hopkins University, USA | NCBI SRA accession number: SRP058681 | [27] |
| Genital anaerobic bacterial overgrowth and the PrePex male circumcision device, Rakai, Uganda | To assess the microbiome in the subpreputial space at time of PrePex removal and compared this to the microbiome in uncircumcised men. | 2 men (median age 20.0) 7 days after PrePex device placement and 145 uncircumcised men (median age 30 years). | NA | Rakai | NA | The Journal of Infectious Diseases | Open | Northern Arizona University & Johns Hopkins University & Translational Genomics Research Institute, USA | Johns Hopkins University, USA & Rakai Health Sciences Program, Uganda | NA | [28] |
| Penile anaerobic dysbiosis as a risk factor for HIV infection | To compare the microbiome and cytokine levels in the penile coronal sulci in uncircumcised men who seroconverted (cases) and uncircumcised controls who remained persistently HIV seronegative (controls) during a randomized-controlled trial of medical male circumcision in Rakai, Uganda. | Uncircumcised men 15 to 49 years old. 46 of them infected with HIV (cases) and 136 remained uninfected (controls). | NA | Rakai (Uganda) | NA | mBio | Open | George Washington University & Translational Genomics Research Institute, USA | George Washington University & Translational Genomics Research Institute, USA | NA | [29] |
| The effects of circumcision on the penis microbiome | To assess the penile microbiota in 12 HIV-negative Ugandan men before and after circumcision. | HIV-negative uncircumcised men aged 15-49 years. | NA | NA | NA | PLoS One | Open | Translational Genomics Research Institute, USA | Johns Hopskin Uinversity, USA | NA | [159] |
| The Effect of Antiretroviral Therapy Initiation on the Vaginal Microbiome in HIV-Infected Women | To characterize how antiretroviral therapy (ART) initiation and CD4 T-cell reconstitution affect the vaginal microbiome among women living with HIV in Rakai, Uganda. | Women aged 18 years and older who are coinfected with HIV and herpes simplex virus type 2 (HSV-2) with CD4 T-cell counts of 300–400 cells/μL (n = 440). | NA | Rakai | NA | Open Forum Infectious Diseases | Open | George Washington University, USA | Johns Hopkins School of Medicine & National Institutes of Health, USA | NA | [160] |
| Highly diverse anaerobe-predominant vaginal microbiota among HIV-infected pregnant women in Zambia | To investigate whether HIV and its treatment alter the microbiome in pregnancy. | Participants are enrolled prior to 24 weeks’ gestation. Of 261 vaginal swabs, 256 (98%) had evaluable sequences; 98 (38%) were from HIV+ participants, 55 (56%) of whom had preconceptional ART exposure. The median age of participants at enrolment was 27 (IQR 22,32). | NA | Lusaka | NA | PLoS One | Open | University of North Carolina, USA & University of Zambia School of Medicine & UNC Global Projects Zambia, Zambia | University of North Carolina at Chapel Hill School of Medicine, USA | Open Science Framework (OSF) DOI: 10.17605/OSF. IO/CJKDW | [161] |
| HIV infection is associated with preterm delivery independent of vaginal microbiota in pregnant African women | To explore the relationship between the composition of vaginal microbial communities, immune factors and pregnancy outcomes in pregnant women living with HIV (PWLH). | Pregnant African women who were HIV-uninfected (n=314) versus HIV-infected (n=42), between 15-35 weeks of gestation, above 18 years of age without sexually transmitted infection (STI) in the past month, or antibiotic use in the past month, excluding cotrimoxazole prophylaxis. | NA | Harare, Chitungwiza | NA | Journal of the Infectious Diseases | Not open | University of Cape Town, South Africa & National Microbiology Reference Laboratory & University of Zimbabwe, Zimbabwe | University of Cape Town, South Africa & Seattle Children’s Research Institute & University of Washington, USA | NA | [162] |

**Supplementary Table S2c. Additional summary of African Microbiome studies (Other body sites).**

| Title | | Aim | Participant Information | Ethnicity | Region, District or Town | Rural vs Urban | Name of Journal | Accessibility | First Author Affiliation (Country) | Last Author Affiliation (Country) | Data Availability | **Reference** |
| --- | --- | --- | --- | --- | --- | --- | --- | --- | --- | --- | --- | --- |
| EYE | | | | | | | | | | | |  |
| Conjunctival Microbiome-Host Responses Are Associated With Impaired Epithelial Cell Health in Both Early and Late Stages of Trachoma | | To investigate the relationship between the conjunctival microbiome and host conjunctival-associated lymphoid tissue responses. | Children with a normal, healthy conjunctiva (n=36) or active trachoma (n=49), and adults with a normal, healthy conjunctiva (n=121) or scarring trachoma (n=158). | Jola, Mandinka, Wolof, and Others | Basse, Brikama, Janjanbureh, Kanifing, Kerewan, Kuntaut, and Mansa Konko | NA | Frontiers in cellular and infection microbiology | Open | London School of Hygiene and Tropical Medicine, UK | London School of Hygiene and Tropical Medicine, UK & MRC Unit the Gambia at LSHTM, Gambia | NCBI SRA accession numbers: PRJNA248889 and PRNA515408 | [163] |
| The conjunctival microbiome in health and trachomatous disease: a case control study | | To characterize the microbiome of the conjunctiva of residents of The Gambia. | 115 patients with clinical signs of trachoma and 105 individuals with normal healthy conjunctiva matched for age, sex, ethnicity and location. Adults and children of both sex. | Wolof, Mandinka, Jola, Fula, Serere, Manjago, Balanta, and Bambara | Banju, Western Division, Lower River Division, Central River Division, Upper River Division, and North Bank Division | NA | BMC Genome Medicine | Open | Washington University, USA | London School of Hygiene and Tropical Medicine, UK & Medical Research Council Unit, The Gambia | NCBI SRA accession number: PRJNA248889 | [164] |
|  | ANTERIOR NARES, NASO- AND OROPHARYNX | | | | | | | | | | | |
| The nasopharyngeal microbiota of children with respiratory infections in Botswana | | To assess whether pneumonia and upper respiratory infection symptoms are associated with specific alterations of the nasopharyngeal microbiota and to determine the effect of HIV infection or exposure on the nasopharyngeal microbiota and to describe age-related differences in the nasopharyngeal microbiota of African children. | Children, 1-23 months of age, with pneumonia (n= 204), and without pneumonia (children with upper respiratory infection symptoms (n=55) and healthy children (n=60)). | NA | Gaborone | NA | The Pediatric Infectious Disease Journal | Not-open | Botswana-UPenn Partnership, Botswana & Duke University Medical Center, USA | Duke University Medical Center, USA | NA | [165] |
| Pneumococcal Colonization and the Nasopharyngeal Microbiota of Children in Botswan | | To understand the interactions between S. pneumoniae and the nasopharyngeal microbiota of children. | Control group comprised of children 1 to 23 months of age (mean age 8.3 months) receiving well child or acute care services at one of 18 public clinics in the Gaborone area. Controls were matched to pneumonia cases 1:1 by primary care clinic and date (≤2 weeks from the case enrollment). | NA | The Gaborone area | NA | The Pediatric Infectious Disease Journal | Open | Botswana-UPenn Partnership, Botswana & Duke University Medical Center, USA | Ann and Robert H. Lurie Children’s Hospital & Northwestern University, USA | NCBI SRA accession number: PRJNA423191 | [166] |
| Effects of vaccination with 10-Valent pneumococcal non-typeable Haemophilus influenza Protein D Conjugate Vaccine (PHiD-CV) on the nasopharyngeal microbiome of Kenyan toddlers | | To assess the nasopharyngeal microbiome of children who had been vaccinated with 10-valent pneumococcal non-typeable Haemophilus influenzae protein conjugate vaccine (PHiD-CV). | Children aged 12-59 months (25 in intervention group and 29 in control group), six subjects (five subjects in the intervention group and one in the control group) were excluded for further analysis. | NA | Malindi District | Rural | PLoS One | Open | University of Colorado, USA | Centre for Geographic Medicine-Coast, Kenya & Johns Hopkins Bloomberg School of Public Health, USA | NCBI SRA BioProject: PRJNA229922 | [167] |
| Human Nasal Microbiome as Characterized by Metagenomics Differs Markedly Between Rural and Industrial Communities in Egypt | | To explore the microbial communities and study the effect of occupational exposure on microbiome structure alterations, which, in turn, might help in prevention of allergic and autoimmune diseases in the future. | Healthy male subjects older than 18 years of age. Samples from 9 Egyptian farmers, living in a village setting in north-east Egypt (Nuba, Sharqyiah Governorate) and 10 samples from ceramic factory workers in a major industrial Egyptian city (Tenth of Ramadan City, Cairo). | NA | North-east Egypt (Nuba, Sharqyiah Governorate) and (Tenth of Ramadan City, Cairo) | Urban and Rural | OMICS A Journal of Integrative Biology | Not open | Ministry of Health, Egypt | Suez Canal University, Egypt | NCBI SRA accession number: PRJNA488874 | [168] |
| Comparing the anterior nare bacterial community of two discrete human populations using Illumina amplicon sequencing | | To assess the anterior nare bacterial diversity among and between the indigenous Pygmy population of Gabon, and a population within Germany. | 98 healthy volunteers from Gabon (adults and children aged 1 month-84 years) and 92 from Germany (adults only aged 19-65 years). | Babongo Pygmy tribe (Gabon) | Ikobé region, Lower Saxony and North Rhine-Westphalia regions (Germany) | Rural (Gabon) | Environmental Microbiology | Not-open | Helmholtz Centre for Infection Research, Germany | Helmholtz Centre for Infection Research, Germany | NA | [169] |
|  | LUNG | | | | | | | | | | | |
| Household air pollution and the lung microbiome of healthy adults in Malawi: a cross-sectional study | | To examined whether the presence of black carbon in alveolar macrophages was associated with alterations in the lung microbiome in a Malawi population. | Healthy, non-smoking, HIV-negative adult aged 18 to 50 years. | NA | Blantyre | Peri-urban | BMC Microbiology | Open | Liverpool School of Tropical Medicine, UK & Malawi-Liverpool-Wellcome Trust Clinical Research Programme, Malawi | Liverpool School of Tropical Medicine, UK & Malawi-Liverpool-Wellcome Trust Clinical Research Programme, Malawi | NCBI SRA accession number: SRP043676, LABARCHIVES: H4513W8T | [170] |
| The lung microbiome in children with HIVbronchiectasis: a cross-sectional pilot study | | To determine the lung microbiome in HIV-associated bronchiectasis and to assess its association with pulmonary exacerbations. | 22 children (68% male; mean age 10.8 years) with HIV-associated bronchiectasis and a control group of 5 children with cystic fibrosis (CF). All subjects had to have been on antiretroviral therapy for a minimum of 6 months prior to enrolment. All children at the clinic are screened routinely every 3 months for TB and none of the subjects had positive TB cultures. | NA | Tshwane Metropolitan region in Gauteng, Mpumalanga province | Rural (Mpumalanga province) and Urban/Pre-urban (Tshwane) | BMC Pulmonary Medicine | Open | University of Pretoria & University of KwaZulu-Natal, South Africa | University of Pretoria, South Africa | Available upon request | [171] |
| Immune response and mortality risk relate to distinct lung microbiomes in patients with HIV and pneumonia | | To investigate whether microbiologically and immunologically distinct subsets of patients with HIV and pneumonia exist and are related to mortality. | HIV-infected patients with acute pneumonia. | NA | Kampala | NA | American Journal of Respiratory and Critical Care Medicine | Open | San Francisco General Hospital, USA | San Francisco General Hospital, USA | NCBI SRA accession number: SRP077299 | [172] |
|  | MOUTH | | | | | | | | | | | |
| The saliva microbiome of Pan and Homo | | To analyze the saliva microbiomes of chimpanzees (Pan troglodytes) and bonobos (Pan paniscus) from two sanctuaries in Africa, and from human workers at each sanctuary. | Staff members of zoos, 20-40 years old from Sierra Leone (n=13) and Democratic Republic of the Congo (n=15) and n=22 Chimpanzees (Sierra Leone) n=23 Bonobos (Democratic Republic of the Congo). | NA | Kinshasa (Democratic Republic of the Congo) and Freetown (Sierra Leone) | NA | BMC Microbiology | Open | Max Planck Institute for Evolutionary Anthropology, Germany & Chinese Academy of Sciences, China | Max Planck Institute for Evolutionary Anthropology, Germany | NCBI SRA accession number: SRP015938 | [173] |
| High diversity of the saliva microbiome in Batwa Pygmies | | To describe the saliva microbiome diversity in Batwa Pygmies, a former hunter-gatherer group from Uganda, using next-generation sequencing of partial 16S rRNA sequences. | 39 participants from Uganda (former hunter-gatherer group), 20 from Democratic Republic of the Congo and 13 from Sierra Leone (agricultural group). | Batwa pygmy (Uganda) | Mpungo, Mukongo, Kitariro, Nyakatare, Bikuto communities of Buhoma (Uganda), Kinshasa (Democratic Republic of the Congo), and Freetown (Sierra Leone) | Rural (Uganda) | PLoS One | Open | Max Planck Institute for Evolutionary Anthropology, Germany | Max Planck Institute for Evolutionary Anthropology, Germany | NA | [174] |
| Illumina MiSeq Sequencing for Preliminary Analysis of Microbiome Causing Primary Endodontic Infections in Egypt | | To investigate the microbial diversity of primary endodontic infections using Illumina MiSeq sequencing platform in Egyptian patients | Adult patients (15 females and 4 males; aged 18 to 51 years; mean age 31 years) without systemic disease, cancer, diabetes, immunodeficiency disorder, and a history of using antibiotics or fluoride in the previous 3 months. Only teeth from adult patients with carious lesions, necrotic pulps, and radiographic evidence of apical periradicular disease were sampled in this study. | NA | Suez | NA | International Journal of Microbiology | Open | Suez Canal University, Egypt | Suez Canal University, Egypt | NCBI SRA accession numbers: PRJNA388365 and SRP108240 | [175] |
| Exome capture from saliva produces high quality genomic and metagenomic data | | To illustrate the utility of exome sequencing via saliva. | 2 KhoeSan families, both adults and children (four individuals from family 1, a quartet with two daughters, three individuals from family 2, an extended family of half-siblings and first cousins, and 8 purportedly unrelated individuals). | KhoSean | Upington | Rural | BMC Genomics | Open | Stanford University & University of Michigan, USA | Stanford University & Stony Brook University, USA | NCBI SRA accession number: SRP038015, SRP036155 | [176] |
| Comparison of the bacterial composition and structure in symptomatic and asymptomatic endodontic infections associated with root-filled teeth using pyrosequencing | | To assess and evaluate the bacterial composition and structure in previously root-filled canals in relation to different disease characteristics. | 17 symptomatic and 23 asymptomatic patients scheduled for endodontic retreatment, 31 females and 9 males. 10 samples were excluded (8 symptomatic and 2 asymptomatic). | NA | Khartoum | NA | PLoS One | Open | Albert-Ludwigs-University, Germany | Albert-Ludwigs-University, Germany | NCBI SRA accession number: SRP029320 | [177] |
|  | BREAST | | | | | | | | | | | |
| Microbiota network and mathematic microbe mutualism in colostrum and mature milk collected in two different geographic areas: Italy versus Burundi | | To evaluate the microbiota network, and differences, of colostrum and mature milk in mothers living in two completely different sites and environments, such as Italy and Burundi. | Healthy mothers who had healthy infants, 20 from Italy and 30 from Burundi. | NA | Hospital of Ngozi Burundi | Rural | The ISME Journal | Open | IRCCS Galeazzi Orthopaedic Institute & University of Milan, Italy | University of Pisa, Italy & International Inflammation (in-FLAME) Network of the World Universities Network, Australia | NA | [178] |
| Social networks, cooperative breeding, and the human milk microbiome | | To examine associations between the social environment and the human milk microbiome. | Hunter-gatherer and horticulturalist women and infants in the Central African Republic (hunter-gatherer n=27 [mean maternal age = 27.0 +- 5.3 years; mean time postpartum =14.4 +- 12.2 months]; horticulturalist n=14 [mean maternal age =29.9 +- 7.5 years; mean time postpartum = 7.8 +- 6.6 months]) in March-April (season 1 [end of dry/beginning of wet season] n=18) and July- August (season 2 [height of wet season] n=23) in the Central African Republic. | Aka hunter-gatherers | Congo Basin rainforest | Rural | American Journal of Human Biology | Not open | Washington State University, USA | Washington State University, USA | NA | [179] |
| Influence of Socio-Economic and Psychosocial Profiles on the Human Breast Milk Bacteriome of South African Women | | To describe the bacteriome of human breast milk from a cohort of South African women and the associated factors. | Human breast milk samples were collected from 554 women (median age =25.3 years) at 6–10 weeks postpartum. At enrolment, 53% practised exclusive breastfeeding, 81% had vaginal delivery and 23% delivered preterm infants. | Mixed ancestry and black African isiXhosa ancestry | Mbekweni and T. C. Newman in Drakenstein sub-district | Semi-rural | Nutrients | Open | University of Cape Town, South Africa | University of Cape Town, South Africa | NCBI SRA accession number: PRJNA520889 | [180] |
| Mycobiome Proﬁles in Breast Milk from Healthy Women Depend on Mode of Delivery, Geographic Location, and Interaction with Bacteria | | To determine whether the healthy human breast milk mycobiota is inﬂuenced by geographical location and mode of delivery, as well as to investigate its interaction with bacterial proﬁles in the same samples. | Healthy, lactating women from 4 different geographical locations (20 in each location), including China, South Africa, Finland, and Spain. Subjects were grouped according to mode of delivery: vaginal (n=10 per country) and Caesarean section (C-section) (n=10 per country). All women who delivered via C-section received prophylactic antibiotics, except Finnish women, for whom no prophylaxis is routinely used per the hospital policy. | NA | Beijing (China), Cape Town (South Africa), Southwestern area (Finland), and Valencia (Spain) | NA | Applied and Environmental Microbiology | Open | Institute of Agrochemistry and Food Technology–National Research Council (IATA-CSIC) & FISABIO Foundation, Spain | Institute of Agrochemistry and Food Technology–National Research Council (IATA-CSIC), Spain & University of Turku, Finland | EBI-ENA accession number: PRJEB25581 and Samples accession numbers: ERS2312706 to ERS2312785 | [181] |
| Human Breast Milk NMR Metabolomic Proﬁle across Speciﬁc Geographical Locations and Its Association with the Milk Microbiota | | To investigate the impact of geographical location and mode of delivery on the nuclear magnetic resonance spectroscopy (NMR) metabolic proﬁle of breast milk and its relationship with the milk microbiome. | Healthy mother-child pairs practicing exclusive breastfeeding at sampling time from different populations. No participants used antibiotics after birth or probiotic during perinatal period. Subjects from each country were grouped into two sub-groups according to the mode of delivery, namely either vaginal delivery (n=10 for each country) or caesarean section (n=10 for China, Finland, and Spain; n=9 for South Africa). | NA | Beijing (China), Cape Town (South Africa), Southwestern area (Finland), and Valencia (Spain) | NA | Nutrients | Open | University of Turku, Finland | University of Turku, Finland & Institute of Agrochemistry and Food Technology-National Research Council (IATA-CSIC), Spain | NCBI SRA accession number: SRP082263 and submission ID: SUB1772296 | [182] |
| Breast milk polyamines and microbiota interactions: impact of mode of delivery and geographical location | | To identify and quantify the polyamine levels in human milk obtained from different countries and through different modes of delivery, and to investigate their association with breast milk microbes. | Healthy women after one month of lactation, practicing exclusive breastfeeding with a different modes of delivery, from China (n=10 Cesarean deliveries, n=10 normal deliveries), from South Africa (n=10 Cesarean deliveries, n=8 normal deliveries), from Finland (n=10 Cesarean deliveries, n=10 normal deliveries), from Spain (n=10 Cesarean deliveries, n=10 normal deliveries). | NA | NA | NA | Annals of Nutrition and Metabolism | Not-open | University of Turku, Finland | Institute of Agrochemistry and Food Technology-National Research Council (IATA-CSIC), Spain | NCBI SRA accession number: SRP082263 and submission ID: SUB1772296 | [183] |
| Distinct patterns in human milk microbiota and fatty acid profiles across specific geographic locations | | To identify the impact of four different geographical locations: Asia, Africa, and North and South Europe on breast milk composition. | Healthy women, 20 each from South Africa, Spain, Finland and China grouped into mode of delivery vagina (n=10) and cesarean section (n=10). | NA | Cape Town (South Africa), Bejing area (China), Southern Western area (Finland), and Valencia (Spain) | Urban | Frontiers in Microbiology | Open | University of Turku, Finland | University of Turku, Finland | NCBI SRA accession number: SRP082263, submission ID: SUB1772296 | [184] |
|  | BLOOD AND PLASMA | | | | | | | | | | | |
| Diagnosis of bacterial bloodstream infections: a 16S metagenomics approach | | To identify bacteria in the blood of 75 children with severe febrile illness in Nanoro, Burkina Faso. | Children (median age 15 months) with severe febrile illness. | NA | Nanoro | NA | PLoS Neglected Tropical Diseases | Open | University of Western Australia, Australia | Institute of Tropical Medicine Antwerp, Belgium | NA | [185] |
| Utility of metagenomic next-generation sequencing for characterization of HIV and human pegivirus diversity | | To apply Next-Generation Sequencing (NGS) for full-genome viral sequencing and characterization of HIV-1 recombinant strains and HPgV from blood donors in Cameroon. | HIV-1-infected Cameroonian blood donor specimens. | NA | Daouala and Yaoundé | NA | PLoS One | Open | Infectious Disease Research, Abbott Park, USA | Infectious Disease Research, Abbott Park, USA | GenBank accession number: KP718914-32 | [186] |
| The plasma virome of febrile adult Kenyans shows frequent parvovirus B19 infections and a novel arbovirus (Kadipiro virus) | | To describe the plasma virome and identify emerging viruses in a population of adults with unexplained fever from East Africa. | Adults with unexplained fever 18 to 35 years with HIV-1-seronegative status or unknow HIV status. | NA | Mtwapa and Kilifi | Peri-urban | The Journal of general virology | Open | Blood Systems Research Institute, USA & Kenya Medical Research Insitute, Kenya | Blood Systems Research Institute & University of Carlifonia at San Franscisco, USA | NCBI SRA under GenBank accession number: SRP090133 | [187] |
| Discovery of novel rhabdoviruses in the blood of healthy individuals from West Africa | | To identify RNA viruses in the blood of patients with unexplained febrile illness and compared them with those found in apparently healthy (i.e., no overt signs of illness) control individuals, all from communities in South-Eastern Nigeria. | 195 participants with unexplained acute febrile illness and 328 healthy men and women from all age groups and socioeconomic backgrounds. | NA | Irrua (South Eastern Nigeria) | Peri-urban village | PLOS Neglected Tropical Diseases | Open | Harvard University & Broad Institute, USA | Irrua Specialist Teaching Hospital & Redeemer's University, Nigeria | NCBI SRA BioProject ID: PRJNA271229 | [188] |
| Investigation of the Plasma Virome from Cases of Unexplained Febrile Illness in Tanzania from 2013 to 2014: a Comparative Analysis between Unbiased and VirCapSeq-VERT HighThroughput Sequencing Approaches | | To characterize the plasma virome of Tanzanian patients with unexplained febrile illness by using two high-throughput sequencing methods: unbiased sequencing and VirCapSeq-VERT | Patients diagnosed with febrile illness. | NA | Dar es Salaam | NA | mSphere | Open | Columbia University, USA | Columbia University, USA | NA | [189] |
| AIDS alters the commensal plasma virome | | To analyze the DNA and RNA virome in the plasma of HIV positive subjects with low versus high CD4 T cell counts from USA and The Gambia. | 23 Ugandans, 32 years old on average (35% male, 65% Female and 43% were on standard antiretroviral therapy) and 12 male USA subjects (45 years old on average and 92% on ART). | NA | NA | NA | Journal of Virology | Open | Blood Systems Research Institute & University of California, USA | Blood Systems Research Institute & University of California, USA | DNA Data Bank of Japan accession number: SRA091349 | [190] |
|  | BRAIN AND SPINAL CORD | | | | | | | | | | | |
| Viral metagenomics revealed novel betatorquevirus species in pediatric inpatients with encephalitis/ meningoencephalitis from Ghana. | | To elucidate the virome composition of the cerebrospinal fluid in children with high fever and clinical signs of CNS infection in a malaria-holoendemic area of the Asante Akim North District, Ghana. | Children (n = 70) aged 1 month to 15 years with a tympanic temperature ≥38 °C and clinical suspicion for encephalitis/meningoencephalitis admitted to the pediatric ward were enrolled in the study. Healthy children (n = 120) from vaccination clinics from the study hospitals’ catchment area under 15 years of age with a tympanic temperature <37.5 °C and without clinical signs of infection were enrolled as a healthy cohort. | NA | Agogo, the Asante Akim North District | NA | Scientific Reports | Open | Bernhard Nocht Institute for Tropical Medicine & Hamburg-Borstel-Lübeck-Riems, Germany | Hamburg-Borstel-Lübeck-Riems & Bernhard Nocht Institute for Tropical Medicine, Germany | GenBank accession numbers: MH017546-MH017587 | [191] |
|  | HAND | | | | | | | | | | | |
| Hand bacterial communities vary across two different human populations | | To assess bacterial communities on the hands of women in Tanzania and compare them to those on the hands of US women. | 29 adult women from Tanzania (who were caregivers to children <5 years and lived in a low-income urban community) and 15 from USA (13 were white and of European ancestry, and two were Chinese American). | NA | NA | Urban | Microbiology | Open | Yale University, USA | Yale University, USA | MG-RAST database accession number: 4503472.3, 4503473.3, 4503474.3 and 4503475.3 | [192] |
|  | SKIN | | | | | | | | | | | |
| Bacterial diversity in Buruli ulcer skin lesions: challenges in the clinical microbiome analysis of a skin disease | | To compare the microbial population associated with Buruli ulcer and non-Buruli ulcer skin samples from patients with ulcers of different etiology, collected in Benin, and healthy skin samples from similar body sites, collected from the same Buruli ulcer endemic area and from public datasets in order to look for shifts in the microbial diversity associated with M. ulcerans disease. | 5 patients with confirmed Buruli ulcer lesions (Benin), 3 non-Buruli ulcer lesions (Benin) and 3 healthy skin samples sequences from publicly available data (1 Benin and 2 USA). | NA | Allada (Benin) | NA | PLoS One | Open | Institute of Tropical Medicine, Belgium | Institute of Tropical Medicine, Belgium | EBI-ENA project number: PRJEB14948 | [193] |
| Skin Microbiome Differences in Atopic Dermatitis and Healthy Controls in Egyptian Children and Adults, and Association with Serum Immunoglobulin E | | To describe patterns in composition, structure, and functional potential in bacterial communities of patients with Atopic Dermatitis in comparison to healthy subjects using 16S rRNA microbial analysis at different ages. | All subjects were either male or female between 2 and 60 years of age. 75 patients with Atopic Dermatitis and 20 healthy controls. | NA | Assiut | NA | OMICS A Journal of Integrative Biology | Not open | Al-Azhar University, Egypt | Suez Canal University, Egypt | NCBI SRA accession number: PRJNA481999 | [194] |
| Environmental influences on the skin microbiome of humans and cattle in rural Madagascar | | To assess the influence of environmental contact on the composition of skin microbial communities among individuals living in rural Madagascar, where people interact closely with an environment shared by cattle, chickens, pigs and other domesticated animals. | 20 adult males aged 18-75 years. 10 of them owned and regularly worked with zebu while 10 do not come into regular contact with zebu. | NA | Madena | Rural | Evolution, Medicine, and Public Health | Open | Duke University, USA | Duke University, USA | NA | [195] |
| Antibacterial soap use impacts skin microbial communities in rural Madagascar | | To assess the impact of antibacterial soap on skin bacterial communities in a population in rural Madagascar. | Adult males aged from 18 to 75 years without clinically elevated health measurements, open wounds, infirmity, or illness. Each individual was sampled at four skin sites (right ankle, right medial forearm, right outer hand, right armpit). | NA | Mandena located in SAVA region (Sambava, Antahala, Vohémar, and Andapa) | Rural | PLoS One | Open | Duke University, USA | Duke University & Duke Global Health Institute, USA | GenBank accession number: KBXC00000000 | [196] |
| Novel phages of healthy skin metaviromes from South Africa | | To better understand the composition of phage communities on skin from South African. | Male subjects between 18 and 30 years of age. Volunteers were given a standard bland shampoo to be used three times a week at home. Three skin areas were sampled: outer forearm (F; dry), scalp (S; sebaceous) and axilla (AX; moist). | NA | NA | NA | Scientific Reports | Open | University of the Western Cape, South Africa | University of the Western Cape, South Africa | GenBank accession numbers: MF417837-MF417995 | [197] |
|  | MORE THAN ONE BODY SITE | | | | | | | | | | | |
| Response of the human gut and saliva microbiome to urbanization in cameroon | | To investigate potential associations between a variety of dietary, medical, parasitological and socio-cultural factors and the gut and saliva microbiomes of individuals from three populations along an urbanization gradient. | Healthy participants, i.e. not taking medication for any infectious or metabolic disease or suffering from specific symptoms (e.g. fever). They were between 18 and 65 years old and were not related at the second degree. 81 individuals in Ngoantet (rural), 34 in Mbalmayo (semi-urban) and 32 in Yaoundé (urban). | NA | Ngoantet, Mbalmayo, and Yaoundé | Rural, Semi-urban, and Urban | Scientific Reports | Open | University of Paris, France | University of Paris, France | EBI-ENA accession number: PRJEB30836 | [46] |
| What’s Normal? Microbiomes in Human Milk and Infant Feces Are Related to Each Other but Vary Geographically: The INSPIRE Study | | To describe microbiomes of milk produced by relatively healthy women living at diverse international sites and compare these to the fecal microbiomes of their relatively healthy infants. | Milk and infant feces were collected from mother/infant dyads living in 11 international sites (rural Ethiopia (n=40), urban Ethiopia (n=26), rural Gambia (n=37), urban Gambia (n=36), Ghana (n=28), Kenya (n=42), Spain (n=36), Sweden (n=22), Peru (n=42), California (n=12), Washington (n=39) (USA)). Women had to be breastfeeding or pumping ≥ 5 times/d and be ≥ 18 y of age. To be included, infants had to be described as healthy by their mothers, have no signs and/or symptoms of acute illness (fever, vomiting, severe cough, diarrhea, or rapid breathing) in the previous 7 days, and have not taken antibiotics in the previous 30 days. | Nordic (Sweden), Unspecified ethinicity (Southeastern Washington), Hispanic, (Northwestern Idaho), Sidama (Ethiopian), Mandika (Gambia), Krobo ot Dangme (Ghana), multiethnic (Kenya) | Madrid, Zaragoza, Huesca, and Vizcaya (Spain), Helsingborg (Sweden), Lima (Peru), Southern California, Southeastern Washington and Northwestern Idaho (USA), Hawassa (Ethiopia), Bakauarea and West Kiang region (Gambia), Southeastern Ghana, and Nakuru (Kenya) | Rural and Urban (Ethiopia, The Gambia) | Frontiers in Nutrition | Open | University of Idaho, USA | University of Idaho, USA | NA | [198] |
| The human microbiota is associated with cardiometabolic risk across the epidemiologic transition. | | To determine the association between the gut (stool-derived) and oral (saliva-derived) microbiota and elevated Cardiometabolic risk. | Men and women aged 25–45 years, from Ghana (n=196), South Africa (n=176), Jamaica (n=92) and the US (n=191) who do not have an infectious diseases including HIV, not pregnant or lactating, and able to participate in normal physical activities. The average age among all the participants was 34.9 ± 6.4 years. | NA | NA | NA | PLoS One | Open | University of Chicago, USA | Loyola University Chicago, USA | QiiTA Database under study ID 11888 and in EBI-ENA under accession number: ERP115612 | [199] |
| Airway response to respiratory syncytial virus has incidental antibacterial effects | | To determine whether elements of the innate immune response to respiratory synctial virus (RSV) infection in the human airway has incidental antibacterial activity by the simultaneous analysis of the upper airway proteomes and microbiota of infants and children with and without RSV infection. | Children (RSV-positive (n=40) and RSV-negative (n=44)). In the RSV-positive group, only children who were positive for RSV (real-time cycle threshold values ≤ 27) and negative for all other targets on the multiplex panel were included. In the RSV negative group, only children who were negative for all pathogens in the real-time PCR panel were included. Children with positive blood culture results were excluded from the study. | NA | Kilifi County | Rural | Nature Communications | Open | KEMRI-Wellcome Trust Research Programme, Kenya & University of Oxford, UK | University of Oxford & the NIHR Oxford Biomedical Research Centre, UK | EBI-ENA accession numbers: PXD009403 and PRJEB28053 | [200] |
| Novel cyclovirus in human cerebrospinal fluid, Malawi, 2010-2011 | | To identify unknown human viruses in paraplegic patients. | Adults patients with unexplained paraplegia. | NA | Blantyre | NA | Emerging Infectious Diseases | Open | Center for Tropical Medicine, The Netherlands | ViroClinics BioSciences BV & Erasmus Medical Center, The Netherlands | NA | [201] |
| Bacterial communities found in placental tissues are associated with severe chorioamnionitis and adverse birth outcomes | | To describe the microbiota found in placental tissue and fetal membranes (chorion and amnion) from a cohort of women in rural southern Malawi. | Women less than 20 weeks’ gestation (294 participants excluded based on various reasons). | NA | Mangochi District (Southern Malawi) | Rural | PLoS One | Open | University College London & Great Ormond Street Hospital NHS Foundation Trust, UK | University College London, UK | EBI-ENA study accession number: PRJEB15035 | [202] |
| Evolution of the gut microbiome following acute HIV-1 infection | | To  investigate the evolution of the gut bacteriome and of several clinically relevant eukaryotic viruses following HIV-1 infection in Mozambique. | 49 Mozambican subjects diagnosed with recent HIV-1 infection (RHI) and 54 HIV-1-negative controls were followed for 9–18 months and compared them with 98 chronically HIV-1infected subjects treated with antiretrovirals (n=27) or not (n=71). | NA | Manhiça District (Southern Mozambique) | NA | Microbiome | Open | IrsiCaixa AIDS Research Institute & Universitat Autònoma de Barcelona, Spain | IrsiCaixa AIDS Research Institute & Universitat Autònoma de Barcelona & Universitat de Vic-Universitat Central de Catalunya & Hospital Universitari Germans Trias i Pujol, Spain | NCBI SRA accession number: PRJNA450025 | [203] |
| Metagenomic sequencing at the epicenter ofthe Nigeria 2018 Lassa fever outbreak | | To perform, for the first time, at the epicenter of an unfolding Lassa fever outbreak, metagenomic nanopore sequencing directly from patient samples, an approach dictated by the highly variable genome of the target pathogen. | Samples from suspected Lassa fever patients. | NA | Edo State, Ondo, Ebonyi | NA | Science | Open | National Infection Service & University of Liverpool, UK & Bernhard Nocht Institute for Tropical Medicine, Germany | Bernhard Nocht Institute for Tropical Medicine & German Center forI nfection Research (DZIF), Germany | GenBank under BioProjects: PRJNA482058, PRJNA482054 and PRJNA482058 | [204] |
| Metagenomic Sequencing of HIV-1 in the Blood and Female Genital Tract Reveals Little Quasispecies Diversity during Acute Infection | | To identify intrahost single nucleotide variants (iSNVs) and to characterize within-sample HIV-1 diversity. | Female, 18 to 23 years old, HIV-1 uninfected, and sexually active. Paired plasma and cervicovaginal lavage samples from three subjects diagnosed with HIV-1 infection. Women are screened for HIV-1 infection by fingerstick testing every 3 or 4 days. | NA | Durban (Kwazulu-Natal province) | NA | Journal of Virology | Open | Massachusetts General Hospital & Broad Institute & Harvard Medical School, USA | Massachusetts General Hospital & Harvard Medical School & Ragon Institute of MGH, MIT, and Harvard, USA | NCBI SRA accession number: PRJNA473698 | [205] |
| Links between environment, diet, and the hunter-gatherer microbiome | | To provide a view of human-associated microbes unperturbed by industrialization, as well as a window into the microbiota that co-evolved with humans. | Healthy men, women, and children who have historically subsisted on five groups of foraged and hunted foods: berries, honey, baobab, tubers, and meat. | Hadza hunter-gatherer | The Central Rift Valley (Tanzania) | Rural | Gut Microbes | Open | Stanford University School of Medicine, USA | Stanford University School of Medicine & Chan Zuckerberg Biohub, USA | QiiTA Database under study ID 11358, ID 4944, ID 3753, and ID 3755), EBI-ENA accession number: ERP109605, and NCBI SRA accession number: PRJNA392012 and PRJNA392180 | [206] |
| Microbiota at multiple body sites during pregnancy in a rural Tanzanian population and the effects of Moringa supplemented probiotic yoghurt | | To assess the influence of probiotic yoghurt containing Lactobacillus rhamnosus GR-1, supplemented with Moringa plant as a source of micronutrients, on the health and oral, gut, vaginal and milk microbiotas of pregnant Tanzanian women. | Healthy pregnant women, 18-40 years at gestational ages of 12-24 weeks. 26 of them received yoghurt daily and 30 were untreated during the last two trimesters and for one month after birth. | NA | Mwanza | Rural | Applied and Environmental Microbiology | Open | Lawson Health Research Institute & The University of Western Ontario, Canada | Lawson Health Research Institute & The University of Western Ontario, Canada | Qiita and EBI Database ID2024 | [22] |
| Gut microbiota in HIV–pneumonia patients is related to peripheral CD4 counts, lung microbiota, and in vitro macrophage dysfunction | | To determine whether large-scale perturbations of the respiratory and gut microbiome occur in parallel and are related to HIV co-morbidities or to disease severity as defined by CD4+ cell counts. | HIV-infected pneumonia patients who do not have pulmonary TB. | NA | Kampala | Urban | Microbiome | Open | University of California San Francisco & Fred Hutchinson Cancer Research Center, USA | University of California San Francisco, USA | EBI-ENA accession number: PRJEB29534 | [207] |
| Metagenomic next-generation sequencing of samples from pediatric febrile illness in Tororo, Uganda | | To conduct an exploratory retrospective mNGS analysis on samples available from a cohort of children hospitalized in rural Uganda with febrile illnesses to characterize potential pathogens associated with fever. | Children (aged 2–54 months) with febrile illness. Their mean age was 16.4 (IQR: 8.0–21.0) months, and 66 (70.2%) were female. Chief symptoms reported in addition to fever were cough (88.3%), vomiting (56.4%), diarrhea (47.9%), and convulsions (27.7%). | NA | Tororo | Rural | PLoS One | Open | University of California, USA | University of California & Chan Zuckerberg Biohub, USA | NCBI SRA accession number: PRJNA483304 and GenBank accession numbers: MH685676-MH685701, MH685703-MH685719, MH684286-MH684293, MH684298-MH684334. | [208] |
| Precision Surveillance for Viral Respiratory Pathogens: Virome Capture Sequencing for the Detection and Genomic Characterization of Severe Acute Respiratory Infection in Uganda | | To detect and characterize viral respiratory pathogens with epidemic potential. | Patients aged ≥2 months presenting to surveillance sites who met SARI case definitions. | NA | Koboko, Kampala, Wakiso, Tororo, Kabrarole, Arua, Mbarara | Rural and Urban | Clinical Infectious Diseases | Open | Columbia University Medical Center, USA | Columbia University Medical Center & Columbia University Mailman School of Public Health, USA | GenBank accession numbers: ARK08222.1 and AIY31287.1 | [209] |
| Rapid sequencing-based diagnosis of infectious bacterial species from meningitis patients in Zambia | | To examine the accuracy and time efficiency of nanopore technology-based sequencer, MinION for the detection of the causative bacteria of 11 meningitis patients in Zambia. | Meningitis patients from 14 days to 52 years. 3 are of unknown age. 7 males, 4 females. | NA | NA | NA | Clinical and Translational Immunology | Open | Tokai University School of Medicine & Tokai University, Japan | Tokai University School of Medicine, Japan | DNA Data Bank of Japan accession numbers: DRR172340-DRR172351 | **[210]** |
